# Quantitative assessment of insertion sequence impact on bacterial genome architecture

**DOI:** 10.1099/mgen.0.000062

**Published:** 2016-07-18

**Authors:** Mark D. Adams, Brian Bishop, Meredith S. Wright

**Affiliations:** J. Craig Venter Institute, 4120 Capricorn Lane, La Jolla, CA 92037, USA

**Keywords:** insertion sequence, mobile genetic element, genome-wide analysis, Acinetobacter baumannii, Klebsiella pneumoniae, antibiotic resistance

## Abstract

Insertion sequence (IS) elements are important mediators of genome plasticity and can lead to phenotypic changes with evolutionary significance. In multidrug-resistant *Acinetobacter baumannii* and *Klebsiella pneumoniae*, IS elements have contributed significantly to the mobilization of genes that encode resistance to antimicrobial drugs. A systematic analysis of IS elements is needed for a more comprehensive understanding of their evolutionary impact. We developed a computational approach (ISseeker) to annotate IS elements in draft genome assemblies and applied the method to analysis of IS elements in all publicly available *A. baumannii*(>1000) and *K. pneumoniae*(>800) genome sequences, in a phylogenetic context. Most IS elements in *A. baumannii*genomes are species-specific IS*Aba* elements, whereas *K. pneumoniae*genomes contain significant numbers of both IS*Kpn* elements and elements that are found throughout the Enterobacteriaceae. *A. baumannii*genomes have a higher density of IS elements than *K. pneumoniae,* averaging ~33 vs ~27 copies per genome. In *K. pneumoniae*, several insertion sites are shared by most genomes in the ST258 clade, whereas in *A. baumannii*, different IS elements are abundant in different phylogenetic groups, even among closely related Global Clone 2 strains. IS elements differ in the distribution of insertion locations relative to genes, with some more likely to disrupt genes and others predominantly in intergenic regions. Several genes and intergenic regions had multiple independent insertion events, suggesting that those events may confer a selective advantage. Genome- and taxon-wide characterization of insertion locations revealed that IS elements have been active contributors to genome diversity in both species.

## Data Summary

1. The ISseeker software has been deposited in Github at http://github.com/JCVI-VIRIFX/ISseeker
2. Tables S3 and S4 provide the GenBank accession numbers to all genome sequences analysed and a URL to retrieve each sequence from the GenBank database.

## Impact Statement

Mobile genetic elements are well recognized for the role they have played in the dissemination of antimicrobial resistance genes in Gram-negative bacteria and in the rise of multi-drug resistance in several human pathogens. With large collections of genome sequences available for many bacterial species, it is now possible to quantify the abundance and distribution of these elements and assess the role they have played in genome evolution. Genome-wide surveys of the locations of insertion sequence (IS) elements in *Acinetobacter baumannii* and *Klebsiella pneumoniae* showed that several different IS elements are common within each species, and that IS elements have made significant contributions to the evolution of genome structure and variation in both species.

## Introduction

Insertion sequences (IS) are mobile genetic elements smaller than ~2 kbp that encode only a transposase. Once acquired, IS elements can spread in a genome by transposition, creating genetic variation and playing important roles in adaptation (Bennett, 2004; Siguier *et al.*, 2014). The density of coding content in bacterial genomes means that most random insertions occur in functional genome regions. Intragenic insertions can cause loss-of-function mutations, while intergenic insertions may disrupt promoter function or can result in up-regulation of adjacent genes in cases where the IS element encodes an outward-facing promoter. Most insertions are presumed to be deleterious, but some may confer a selective advantage. For example, in *Acinetobacter baumannii* an IS*Aba*1 insertion upstream of the chromosomal *ampC* gene results in over-expression of the *Acinetobacter-*derived cephalosporinase (ADC) beta-lactamase and resistance to extended-spectrum cephalosporins (Corvec *et al.*, 2003; Heritier *et al.*, 2006; Turton *et al.*, 2006). In addition to disrupting a gene and modifying gene expression, pairs of IS elements can act as a transposon, mobilizing new genetic material via lateral gene transfer such as the IS*Aba*1-flanked *bla*_OXA-23_ termed Tn*2006* that confers carbapenem resistance (Mugnier *et al.*, 2009). The *bla*_KPC_ carbapenemase is bracketed by IS*Kpn*7 and IS*Kpn*6 in Tn*4401a* (Naas *et al.*, 2008) and the IS*Aba*125 element was involved in the emergence of *bla*_NDM-1_ (Poirel *et al.*, 2012,^ 2011^). Several IS elements have been reported that drive mobilization and expression of* bla*_OXA-58_ (Poirel & Nordmann, 2006) and *bla*_RTG_ (Potron *et al.*, 2009; Bonnin *et al.*, 2012).

Despite the importance of these elements in the evolution of antimicrobial resistance, few studies have addressed their genome-wide distribution across a diverse set of strains. Gaffé *et al*. (2011) found that IS elements contributed significantly to adaptive evolution of *Escherichia coli* under controlled growth conditions in continuous culture. They examined the distribution of eight IS elements in 120 *Escherichia coli* genomes following long-term growth in chemostats, and identified new IS locations that altered the global regulatory program. A study of eight clinical isolates of *E. coli* O157 found that IS629 and ISEc8 caused frequent small-size structural polymorphisms and suggested that IS elements may play a role in the inactivation of incoming phage and plasmids (Ooka *et al.*, 2009). Open questions remain regarding the genome-wide impact of IS elements, the relative abundance and diversity across evolutionary lineages, and the extent to which IS elements may be reshaping the genomes of clinically important pathogens.

**Fig. 1.**
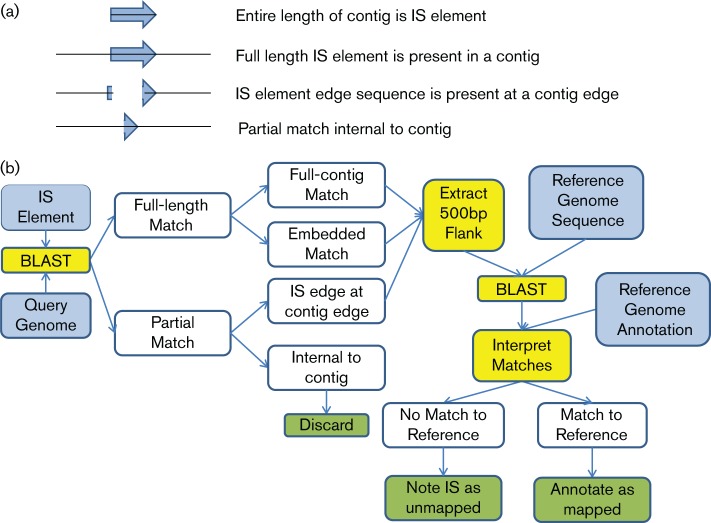
Outline of ISseeker process. (a) Illustration of four possible alignments between an IS element and a query genome. IS element sequences are represented by blue arrows and contig sequences as a solid line. (b) Process diagram for determining IS locations based on alignment of IS flanks with a reference genome. Inputs are in blue boxes and outputs in green boxes. ISseeker program steps are shown in yellow boxes. White boxes depict intermediate results that determine next steps in program execution. Arrows depict the flow of logic in the program.

In draft genome assemblies, multi-copy IS elements are typically collapsed into a single contig that represents the full-length IS element sequence. Each IS copy cannot be placed in its correct genome location during assembly unless long reads, mate pairs, or some other long-range linking strategy is employed. Typically, contigs are broken at IS locations, and sequence reads that span the junction from chromosomal sequence to IS element sequence extend several bases into the IS sequence (Fig. 1a). This extension or ‘stub’ is often approximately half the read length when using the Velvet (Zerbino & Birney, 2008) or SPAdes (Bankevich *et al.*, 2012) assemblers. Three software programs have been described for mapping transposable element locations: ISmapper (Hawkey *et al.*, 2015), TIF (Nakagome *et al.*, 2014), and breseq (Barrick *et al.*, 2014). Each of these programs relies on primary read data rather than sequence assemblies, making them effective at defining junctions, but difficult to apply to large surveys involving hundreds of genomes given the very large input datasets. We developed the ISseeker software to identify flanks of IS elements in genome assemblies – both full length copies in long contiguous sequences and stubs at contig edges – extract the flanking sequences, and align those flanks to a common reference to enable comparison of IS locations across many strains.

Thirty-six *Acinetobacter baumannii* species-specific IS*Aba* elements have been registered with the ISfinder database (https://www-is.biotoul.fr/; Siguier *et al.*, 2006). Twenty-five IS*Kpn* elements are in the ISfinder database. Several of these elements were initially described in genome sequencing projects, while others were identified based on their participation in antibiotic resistance gene mobilization (Tables 1 and 2). *Klebsiella pneumoniae* strains also have elements that are commonly found throughout the Enterobacteriaceae. Other IS elements that have been described in both genomes were included in the analysis as well. ISseeker was used to define the location of IS elements in over 1000 *A. baumannii* genome sequences and in over 800 *K. pneumoniae* genomes. The resulting patterns of IS distribution show that several elements are abundant in both species and that IS elements have played a significant role in genome evolution.

**Table 1.** IS Elements Surveyed in *A. baumannii* genomes

IS element	No. of genomes with:		Distinct sites*					Total sites†					Diversity ratio‡	Linked genes
	Annotated sites	Un-annotated sites	No. of sites	In genes	Between genes	% In genes	% Between genes	No. of sites	In genes	Between genes	% In genes	% Between genes		
IS*26*	776	30	265	208	57	78.5	21.5	3087	1612	1475	52.2	47.8	0.086	
IS*Aba*1	815	20	1229	735	497	59.8	40.4	14510	7383	7127	50.9	49.1	0.085	
IS*Aba*2	46	31	57	43	14	75.4	24.6	185	160	25	86.5	13.5	0.308	
IS*Aba*3	4	12	14	9	5	64.3	35.7	20	13	7	65.0	35.0	0.700	OXA-58
IS*Aba*4	0													
IS*Aba*5	19	31	41	25	16	61.0	39.0	59	36	23	61.0	39.0	0.695	
IS*Aba*6	1													
IS*Aba*7	1													
IS*Aba*8	1	4	4	3	1	75.0	25.0	4	3	1	75.0	25.0	1.000	
IS*Aba*9	2		2		2	0.0	100.0	2		2	0.0	100.0	1.000	RTG-4
IS*Aba*10	38	12	97	59	38	60.8	39.2	241	143	98	59.3	40.7	0.402	OXA-23
IS*Aba*11	12	19	69	39	30	56.5	43.5	85	48	37	56.5	43.5	0.812	LpxA/C
IS*Aba*12	142	122	357	209	148	58.5	41.5	990	592	398	59.8	40.2	0.361	
IS*Aba*13	393	80	572	309	263	54.0	46.0	3639	1865	1774	51.3	48.7	0.157	
IS*Aba*14	13	15	13	9	4	69.2	30.8	25	20	5	80.0	20.0	0.520	RTG-6
IS*Aba*15	0													LpxD
IS*Aba*16	140	15	258	172	86	66.7	33.3	1032	661	371	64.1	35.9	0.250	OXA-51
IS*Aba*17	276	12	98	60	38	61.2	38.8	2007	1119	888	55.8	44.2	0.049	
IS*Aba*18	46	25	60	46	14	76.7	23.3	199	172	27	86.4	13.6	0.302	
IS*Aba*19	62	25	163	132	31	81.0	19.0	387	328	59	84.8	15.2	0.421	
IS*Aba*20	3	1	5	4	1	80.0	20.0	5	4	1	80.0	20.0	1.000	
IS*Aba*21	3	20	12	10	2	83.3	16.7	12	10	2	83.3	16.7	1.000	RTG-5
IS*Aba*22	63	301	71	49	22	69.0	31.0	112	89	23	79.5	20.5	0.634	AadB
IS*Aba*23	0													
IS*Aba*24	200	1	9	7	2	77.8	22.2	240	49	191	20.4	79.6	0.038	
IS*Aba*25	157	19	340	219	121	64.4	35.6	1391	884	507	63.6	36.4	0.244	
IS*Aba*26	381	39	190	136	54	71.6	28.4	652	255	397	39.1	60.9	0.291	
IS*Aba*27	46	20	343	116	227	33.8	66.2	818	258	560	31.5	68.5	0.419	
IS*Aba*28	1	1												
IS*Aba*29	56	25	128	102	26	79.7	20.3	308	263	45	85.4	14.6	0.416	
IS*Aba*30	0													
IS*Aba*31	29	51	93	34	60	36.6	64.5	122	39	83	32.0	68.0	0.762	
IS*Aba*32	0	16												
IS*Aba*33	29	20	64	33	31	51.6	48.4	94	41	53	43.6	56.4	0.681	
IS*Aba*36	42	1	253	172	81	68.0	32.0	712	446	266	62.6	37.4	0.355	
IS*Aba*125	402	160	528	338	190	64.0	36.0	1595	1054	541	66.1	33.9	0.331	OXA-58
IS*Aba*825	3	2	6	3	3	50.0	50.0	6	3	3	50.0	50.0	1.000	OXA-58

*Distinct sites is the number of unique annotated locations on the reference genome.

†Total sites in the number of annotated and unannotated sites summed for all genomes.

‡(Number of distinct insertion sites)/(Total insertions in all genomes).

**Table 2.** IS Elements Surveyed in *K. pneumoniae* genomes

IS element	No. of genomes with:		Distinct sites*					Total sites†					Diversity ratio‡	Linked genes
	Annotated sites	Un-annotated sites	No. of sites	In genes	Between genes	% In genes	% Between genes	No. of sites	In genes	Between genes	% In genes	% Between genes		
IS*1294*	40	16	127	97	30	76.4	23.6	325	227	98	69.8	30.2	0.391	
IS*1F*	452	49	87	52	35	59.8	40.2	1030	75	955	7.3	92.7	0.084	
IS*26*	494	69	211	160	51	75.9	24.2	2064	1577	487	76.4	23.6	0.102	
IS*4321R*	159	33	21	13	8	61.9	38.1	376	141	235	37.5	62.5	0.056	
IS*5*	19	23	44	22	22	50.0	50.0	53	26	27	49.1	50.9	0.830	
IS*5075*	318	26	36	22	14	61.1	38.9	883	292	591	33.1	66.9	0.041	
IS*6100*	430	30	15	11	4	73.3	26.7	937	140	797	14.9	85.1	0.016	
IS*903B*	491	73	194	119	75	61.3	38.7	1403	467	936	33.3	66.7	0.138	
IS*Ecp*1B	90	16	51	35	16	68.6	31.4	136	99	37	72.8	27.2	0.375	
IS*Kpn*1	583	4	146	27	119	18.5	81.5	2967	57	2910	1.9	98.1	0.049	
IS*Kpn*2	0	35												
IS*Kpn*3	0													
IS*Kpn*4	0													
IS*Kpn*6	413	1	3		3	0.0	100.0	823		823	0.0	100.0	0.004	KPC
IS*Kpn*7	414	1	3		3	0.0	100.0	824		824	0.0	100.0	0.004	KPC
IS*Kpn*8	24	5	8	1	7	12.5	87.5	32	3	29	9.4	90.6	0.250	
IS*Kpn*9	0													
IS*Kpn*10	0													
IS*Kpn*11	0	6												
IS*Kpn*12	0	12												
IS*Kpn*13	0													
IS*Kpn*14	64	142	58	33	25	56.9	43.1	98	51	47	52.0	48.0	0.592	
IS*Kpn*15	0													
IS*Kpn*18	387	1	85	76	9	89.4	10.6	1188	1153	35	97.1	2.9	0.072	
IS*Kpn*19	12	16	11	9	2	81.8	18.2	12	10	2	83.3	16.7	0.917	
IS*Kpn*20	3	3												
IS*Kpn*21	47	36	19	9	10	47.4	52.6	80	43	37	53.8	46.3	0.238	
IS*Kpn*23	0													
IS*Kpn*24	235	19	29	20	9	69.0	31.0	458	37	421	8.1	91.9	0.063	
IS*Kpn*25	71	76	13	6	7	46.2	53.8	76	6	70	7.9	92.1	0.171	
IS*Kpn*26	486	33	621	379	242	61.0	39.0	4238	1627	2611	38.4	61.6	0.147	
IS*Kpn*27	0													
IS*Kpn*28	452	25	49	20	29	40.8	59.2	855	166	689	19.4	80.6	0.057	
IS*Kpn*31	0	10												

*Distinct sites is the number of unique annotated locations on the reference genome.

†Total sites in the number of annotated and unannotated sites summed for all genomes.

‡(Number of distinct insertion sites)/(Total insertions in all genomes).

## Methods

ISseeker was written in perl to annotate the locations of a range of IS elements in complete and draft genome sequences. Search results are output in a text file log, a comma-separated values file and as SQL statements that can be loaded to a MySQL database to facilitate complex queries. The outline of the program is illustrated in Fig. 1. ISseeker identifies complete and partial IS matches in a query genome using blastn, with a user-specifiable percent identity threshold (default 97 %). Using contig length information, matches are classified as embedded in a contig (and either full-length or partial), consisting of an entire contig (this is common in draft assemblies), or representing the edge of the IS element matching the edge of a contig. Full-length embedded matches and valid edge matches are selected for annotation. A 500 bp sequence region adjacent to the IS element is extracted from the contig and searched against the reference genome using blastn. Matches are evaluated using user-defined thresholds for percent identity (default 97 %) and length, and those passing the threshold are reported. The location relative to adjacent genes in the reference genome is reported. The program attempts to link matches into pairs representing the start and end of the IS element that map to equivalent sites in the reference genome and thus correspond to a single insertion event. In practice, this is incomplete because it appears that deletions are common near IS elements and it is not obvious whether a single event is represented. Flanks that do not match the reference are also included in the output as ‘unannotated’ flanks. Output is saved in a log file and as SQL statements for bulk import into a MySQL database, which facilitates complex queries. When evaluating the IS locations, we found many instances of IS sites clustered within a few bases of one another. These could represent independent insertion events or alignment artifacts. Manual review suggested that the latter was common, so for the purpose of reporting the number of distinct insertion sites, we bundled annotated locations within 10 bases of each other as a single event. Locations relative to genes were inferred based on the GenBank annotation for the reference genome, with location outside of annotated coding regions designated as intergenic and those inside coding regions designated intragenic. The ISseeker software and the MySQL schema are available at https://github.com/JCVI-VIRIFX/ISseeker.

A user-specified reference genome is required for ISseeker analysis. The TYTH-1 genome sequence [GenBank accession no. CP003856.1 (Liou *et al.*, 2012)] was selected as the reference *A. baumannii* genome after consideration of several completed genome sequences. TYTH-1 was isolated in Taiwan in 2008 and is a GC2 strain (Nemec *et al.*, 2004), as are a majority of strains with genome sequences in the GenBank database. NJST258_1 [GenBank accession no. CP006923.1 (Deleo *et al.*, 2014)] was selected as the *K. pneumoniae* reference genome. NJST258_1 is a KPC-positive ST258 strain isolated in New Jersey, USA, in 2010. All completed and draft *A. baumannii* and *K. pneumoniae* genomes available in the GenBank database as of 1 August 2015 were downloaded. Genome assemblies that were highly fragmented (>300 contigs), or were assembled with newbler, or represented non-*baumannii Acinetobacter* strains or non-*pneumoniae Klebsiella* strains were excluded. 1035 complete and draft *A. baumannii* genomes and 807 complete and draft *K. pneumoniae* genomes were analysed.

All species-specific IS elements cataloged in the ISfinder database (Siguier *et al.*, 2006) were downloaded and compared against the full genome set for each species. In addition, several complete genome sequences for each species were searched against the ISfinder database by BLAST to identify species non-specific elements. Eighteen additional (non-IS*Kpn*) IS elements found in *K. pneumoniae* genomes were analysed and seven additional (non-IS*Aba*) IS elements were analysed in *A. baumannii* genomes. Results are included in Tables 1 and 2 for those elements that were present in more than five genomes.

ISseeker was compared with ISmapper using a set of genomes for which both a finished genome sequence (e.g. a ‘gold standard’) and Illumina short reads were available. Illumina read sets were downloaded from NCBI’s Sequence Read Archive (SRA) using the SRA Toolkit utility fastq-dump. ISmapper was run on each read set using default parameters. Each Illumina read set was assembled using SPAdes (Bankevich *et al.*, 2012). ISseeker was run on the finished genome sequence and on the Illumina assembly. The performance of ISseeker was evaluated by performing runs against the full set of sequences for each species using varying values for percent identity of matches to the IS element and of IS-flanking sequences to the reference, and using an alternative reference genome. It should be noted that the newbler assembler (Miller *et al.*, 2010) suppresses these stubs so newbler assemblies cannot be used by ISseeker.

**Fig. 2.**
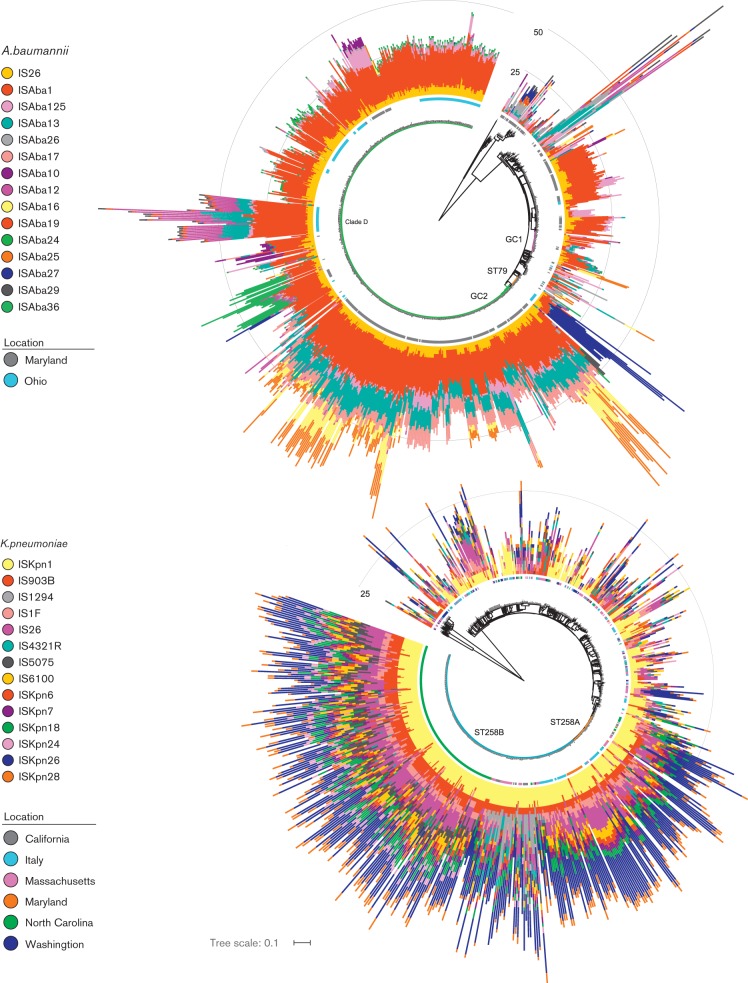
IS representation in a phylogenetic context. The most abundant IS elements (present in >100 genomes) are shown in the context of the *A. baumannii* (a) and *K. pneumoniae* (b) phylogeny based on SNP markers. Isolation locations for the strains from the largest collections have a color code in the inner circle. The height of each bar represents the number of copies of each element in each genome. Scale rings illustrate the height of the histograms on each tree diagram. In (a), strain groups are denoted with coloured branches: Global Clone 1 (pink), GC2 (green) and ST79 (orange). In (b), the two major sub-groups of ST258 are denoted as ST258A (orange) and ST258B (blue).

A core phylogeny based on single-nucleotide variants (SNVs; 278 322 SNVs for *A. baumannii*, 332 571 SNVs for *K. pneumoniae*) was inferred using SNVs identified by NASP (Sahl *et al.*, 2016) and constructed using FastTree 2 (Price *et al.*, 2010). Genome positions with allele calls in at least 80 % of strains were included in the analysis. Fig. 2 was prepared using the graphics tools available through the interactive Tree of Life (iTOL) web service (Letunic & Bork, 2011).

The statistical significance of comparisons of IS element composition between strain sets was assessed using Student’s t-test.

## Results

### Description and evaluation of the ISseeker program

Four classes of IS alignment are considered by the ISseeker program (Fig. 1a): contigs that are comprised entirely of IS sequence, IS element matches that are full-length and embedded in a long genomic contig, matches to the beginning or end of an IS element at the start of end of a contig, and partial matches internal to a contig. Contig sequences flanking each IS element are extracted and compared to a reference genome (Fig. 1b). By mapping all IS/genome junctions to a single reference, it is possible to compare IS locations across strains.

The performance of ISseeker was evaluated from two perspectives: 1) comparison with ISmapper, using IS locations in completely sequenced reference genomes as a gold standard, and 2) to determine the impact of alternative run parameters on the detection of IS element locations. Two other programs that can identify IS elements in short read data were not included in the evaluation because they are not strictly comparable. Breseq was designed for mutation-finding in long-term culture experiments and is best suited to comparing very closely related genomes to a sequenced reference. TIF uses the unix grep command to identify IS-matching reads and is thus unable to identify non-exact matches.

Results from ISseeker and ISmapper were compared on four *K. pneumoniae* genomes and four *A. baumannii* genomes for which Illumina reads, Illumina assemblies, and finished genome sequences were available (Table S1, available in the online Supplementary Material). Across these eight genomes, there were 74 insertion sites for the test IS elements IS*Aba*1 or IS*Kpn*26. ISseeker found all 74 sites when run using both the finished sequences and the draft genome assemblies, while ISmapper missed 20 sites for a sensitivity of 73 %. This is lower than the value reported by Hawkey *et al.* (2015). Further analysis showed that most missed sites were in genomes with low read coverage (<80x) or at locations with structural variation relative to the reference. ISseeker reports every IS-flanking sequence, including locations that cannot be annotated in the reference genome and those that are not in valid pairs matching both the IS element beginning and end sequences at a common reference location. ISmapper is more conservative in reporting only valid IS edge pairs in the primary output, with some additional information in ancillary output files. One interesting case identified by ISseeker, but not ISmapper, involved the creation and mobilization of a compound transposon comprised of inverted repeat copies of IS*Aba*1 in the ORAB01 genome (Fig. S1). ISseeker recognized that there were two copies and the correct location of both, but the details of the structure were only apparent in the finished genome sequence.

The sensitivity to alteration in run parameters was evaluated for an abundant IS element in each species - IS*Aba*1 and IS*Kpn*26 - across the full set of assemblies for each species (Table S2). Reduction of the minimum percent identity of the matches (IS edge detection and flank alignment to the reference) from the default of 97 % to 95 % resulted in annotation of 3–4 % more sites. Upon manual review, some of these were determined to be spurious, so the more conservative threshold was retained. Increasing the stringency of the flank alignment to require a full 500 bp match reduced the number of annotated sites by 13 % (*A. baumannii*) and 8 % (*K. pneumoniae*). Use of an alternative *A. baumannii* reference -a GC1 strain rather than a GC2 strain - also resulted in a loss of about 7 % of the annotated sites.

### Analysis of IS elements in *A. baumannii* and *K. pneumoniae*

Each IS*Aba* and IS*Kpn* element was compared against the corresponding full set of complete and draft genomes (Tables 1, 2, S3 and S4). Several non-species-specific IS elements were also included, based on elements present in a sampling of genomes from each species. 89 % of *A. baumannii* genomes and 94 % of *K. pneumoniae* genomes had at least one IS element detected and several elements were detected in hundreds of genomes (Fig. 2). The overall numbers of insertions and chromosomal locations of IS elements were greater in *A. baumannii* than in *K. pneumoniae.* In *K. pneumoniae*, 18 869 total IS copies were found across 782 genomes. In *A. baumannii*, 32 539 copies were found across 976 genomes. On average, *A. baumannii* genomes contained 33 copies of IS elements, while *K. pneumoniae* genomes contained 27 copies (*p*<0.001).

**Fig. 3.**
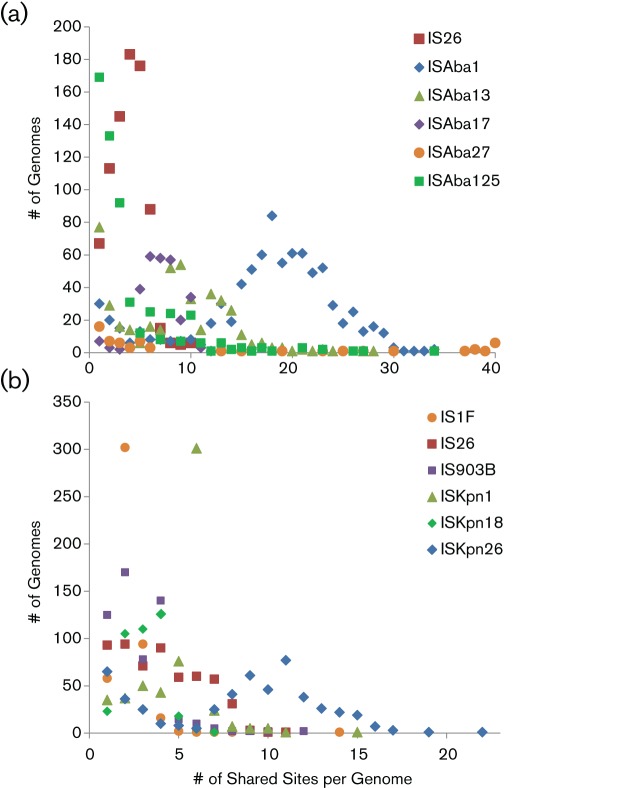
Distribution of conserved IS clusters for the most common IS elements. The number of strains sharing a set of IS element locations is plotted for the five IS elements with the largest number of copies (>1500 total copies for each element) in *A. baumannii* and for IS*Aba*27 that is greatly expanded in certain genomes (a), and for six *K. pneumoniae* IS elements with the largest number of copies (>1000 total copies) (b).

A strong pattern of similar IS content among phylogenetically related strains is apparent, suggesting that many insertions are conserved. The number of genomes that share a set of IS insertion locations for the most abundant elements is shown in Fig. 3. IS*Aba*1 and IS*Kpn*26 are the only elements with >10 shared sites in a substantial number of strains. Four additional IS elements have >10 copies per genome in some *A. baumannii* strains. IS*26* and IS*Kpn*1 have 2 and 6 copies in shared locations per genome, respectively, in 300 strains, corresponding to ST258 strains.

There are more distinct IS element insertions in the examined *A. baumannii* genomes compared to *K. pneumoniae*. With respect to distinct sites mapped to each reference genome (TYTH-1 for *A. baumannii* and NJST258_1 for *K. pneumoniae*), there were 1843 distinct *K. pneumoniae* genome locations with IS insertions and 5341 distinct *A. baumannii* locations. These distinct insertion sites represent the minimum number of insertion events that occurred over time because some insertion sites could not be mapped to the selected reference genomes, and because multiple independent insertions could have occurred at a given site. Twelve different IS elements have over 100 distinct insertion sites across the *A. baumannii* strain set, but only five IS elements have that many distinct insertion sites in *K. pneumoniae* genomes. There are many more IS insertion sites shared by up to 100 *A. baumannii* genomes than there are shared sites across similar numbers of *K. pneumoniae* genomes (Table 3). In contrast, *K. pneumoniae* genomes have more sites shared in >250 genomes reflecting the relatively homogenous IS patterns in the dominant ST258 clade in the dataset. In addition, there are many more strain-specific insertion events in *A. baumannii* (3194 vs 1234). Another view of the extent of shared insertion sites is given in Fig. S2 that depicts the number of genomes that share sites along the *A. baumannii* or *K. pneumoniae* reference chromosome. There are many more moderately abundant shared sites among *A. baumannii* strains than *K. pneumoniae* strains.

**Table 3.** Shared IS element sites

Number of genomes sharing a site	Total copies		Distinct sites	
	*A. baumannii*	*K. pneumoniae*	*A. baumannii*	*K. pneumoniae*
>500	736	0	1	0
250–499	6975	12789	25	36
150–249	3794	3385	18	18
100–149	2561	1136	21	9
50–99	1559	911	21	14
20–49	4985	950	172	32
10–19	2642	612	195	42
2–9	5590	1634	1694	471
1	3194	1234	3194	1234

A majority of genomes in the datasets for both species belong to multidrug-resistant clonal groups that have recently expanded: 62 % of *A. baumannii* genomes belong to GC2 [i.e. multi-locus sequence type (MLST) ST2] and 53 % of *K. pneumoniae* genomes belong to MLST ST258. In these subsets of strains, IS elements are also much more frequent and their locations are more diverse in *A. baumannii* compared to *K. pneumoniae* (see Fig. 2). In *K. pneumoniae*, the common location of IS insertion sites shared by large numbers of ST258 strains regardless of geographic origin supports a model of recent expansion with limited strain- or clade-specific IS gain or loss. Variability in IS element composition and abundance is higher in the *A. baumannii* GC2 set, with more clustering of IS patterns by geographic location and phylogenetic position. It has been hypothesized that the ST258 lineage of *K. pneumoniae* arose around 1995 (Bowers *et al.*, 2015), whereas the oldest known MDR GC2 strain of *A. baumannii* was isolated in 1982 (Diancourt *et al.*, 2010; Blackwell *et al.*, 2015).

### *A. baumannii* IS elements

IS*Aba*1 has had the largest impact on *A. baumannii* genomes, with copies detected in 815 of the *A. baumannii* genome assemblies and over 14 500 total insertions mapped in those strains. An IS*Aba*1 insertion site is present upstream of the *bla*_ADC_ (*ampC*) gene in most of the genomes that have copies of this element (736 genomes). The second most common insertion site for IS*Aba*1 is upstream of the other chromosomal β-lactamase gene, *bla*_OXA-51-like_ (369 genomes). This insertion results in over-expression of the OXA-51-like carbapenemase and resistance to imipenem and meropenem (Nemec *et al.*, 2008). The median number of IS*Aba*1 sites per genome was 19 and the maximum number in a single genome was 34.

Four other elements (IS*Aba*125, IS*Aba*13, IS*Aba*26 and IS*26*) were present in over 350 strains each, and five additional elements were present in more than 100 strains (Table 1). In a few cases, it seems that an IS element has run amok in a genome, such as the IS*Aba*6 and IS*Aba*7 elements in *A. baumannii* strain SDF (Vallenet *et al.*, 2008). Most ST79 strains have 50–100 copies of IS*Aba*27. Seven genomes have copies of 10 or more different IS elements and five genomes have more than 100 total IS copies.

IS*Aba*4, IS*Aba*15, IS*Aba*23, IS*Aba*30 and IS*Aba*32 were not found in any of the sequenced genomes. IS*Aba*6 and IS*Aba*7 were found only in the SDF genome (Vallenet *et al.*, 2008). IS*Aba*8 and IS*Aba*28 were also only found in one genome each. IS*Aba*2, IS*Aba*18, IS*Aba*19 and IS*Aba*29 are IS3-family elements and are 85–95 % identical to one another, making inference of their abundance and correct locations difficult in draft genomes. Likewise, IS*Aba*16 and IS*Aba*25 are 97 % identical to one another and many of their annotated sites overlap with one another and are thus ambiguous as to the specific element that is present at each location. IS*Aba*12 and IS*Aba*13 are 84.8 % identical, including regions of 100 % identity in the first 23 bases and last 21 bases, and are also difficult to discriminate in draft genomes. Of the non-IS*Aba* elements examined, only IS*26* was abundant enough to be included.

Genomes that are closely related to each other on the phylogenetic tree tended to have similar patterns of IS element composition (Fig. 2). There are a few large strain collections representing restricted geographic regions among the 1035 genomes, including 442 isolates from Maryland (NCBI BioProject PRJNA224116) and 174 from Ohio (Wright *et al.*, 2014, 2016). Many of these genomes are very similar, potentially representing clonal series, but differences in IS content are apparent within each group. Among the GC2 genomes, there are several interesting phylogenetic clusters, some of which correspond to geographically restricted strain collections. For example, some clusters of strains isolated in Maryland have copies of IS*Aba*16/IS*Aba*25 that are largely absent from other strains. Strains previously identified as Clade D (Wright *et al.*, 2014, 2016) are clearly distinct from other GC2 strains by having 7–31 copies of IS*Aba*12. Most Ohio strains have one or two copies of IS*Aba*22, IS*Aba*24, and IS*Aba*26 that are found in relatively few other strains. Most Maryland strains have 7–10 copies of IS*Aba*13 and of IS*Aba*17, elements that are not as abundant in other branches of the tree.

### *K. pneumoniae* IS elements

Overall, there are fewer IS element copies in *K. pneumoniae* genomes than in *A. baumannii*. This difference is reflected across both the number of distinct insertion sites (reflecting historical independent insertion events) and in the total number of copies across the genome set (reflecting the success of strains carrying those elements) (Fig. 2, Tables 1 and 2). In *K. pneumoniae*, about 350 ST258 genomes share IS insertion locations for IS*Kpn*1, IS*Kpn*26 and IS*1F*. This suggests that ST258 genomes have spread rapidly worldwide with a reasonably stable repertoire of IS elements and only limited new IS mobilization activity.

Thirteen IS elements were present in >100 *K. pneumoniae* genomes (Table 2, Fig. 2). IS*Kpn*6 and IS*Kpn*7 are present on Tn*4401* that carries the *bla*_KPC_ gene; the presence of those two elements corresponded closely with the presence of the *bla*_KPC_ gene. Both of those elements were only observed in the Tn*4401* context and so appear not to have mobilized to other sites in the *K. pneumoniae* genomes. IS*Kpn*24 is also present on the pNJST258N2 plasmid that carries Tn*4401*,and copies in most ST258 genomes map to that plasmid. Unlike IS*Kpn*6 and IS*Kpn*7, IS*Kpn*24 was observed at several other sites in a subset of genomes.

Eight additional IS elements were found in more than 200 genomes (IS*Kpn*1, IS*Kpn*18, IS*Kpn*26, IS*Kpn*28, IS26, IS*903B*, IS*1F*, and IS*6100*). Of these, ISKpn*18* is almost entirely restricted to ST258 strains, but the other IS elements are found throughout the phylogenetic tree. IS*Kpn*1 was the most broadly distributed, appearing in 583 genomes. Most ST258 genomes share five common IS*Kpn*1 insertion sites. The number of copies of IS*Kpn*26 is more variable across genomes, indicating more active mobilization than other elements. NJST258_1 has seven chromosomal copies of IS*Kpn*26 and one on pNJST258N1. Copies at equivalent positions are present in other ST258 strains. IS*Kpn*28 mapped to another large plasmid (pNJST258N1) in the reference genome, and one copy was present in the chromosome in most ST258 strains.

ST258 genomes have more than three times as many IS copies than non-ST258 genomes (average 34.5 vs 10; *p*<0.01). Unlike in *A. baumannii*, there are no large expansions in IS copy number in any of the *K. pneumoniae* genomes: the maximum number of copies of a single element was 22 copies of IS*Kpn*26 and only 18 genomes have ≥50 total IS insertions, compared to 189 *A. baumannii* genomes with ≥50 elements. Eight IS*Kpn* elements were not found in any sequenced genome.

### Distribution relative to genes

The examined IS elements vary in their insertion locations relative to coding regions. By definition, an element may insert within a gene or between genes. Intragenic insertions have the potential to act as gene knockouts. Intergenic insertions may have no effect on adjacent genes or could either positively or negatively affect expression, depending on the precise location relative to promoters. IS*Aba*1 and IS*Aba*125 have strong outward-facing promoters and can up-regulate the expression of adjacent genes (Lopes & Amyes, 2012); other elements have not been characterized for promoter activity. We considered the fraction of intragenic insertions for each element from two perspectives – the total number of sites across all genomes, and the number of distinct sites (Tables 1 and 2). The former measure incorporates the abundance (number of genomes carrying each insertion) while the latter more accurately reflects the number of insertion events and is not biased by repeated sampling of closely related genomes. The two measures are closely correlated for IS elements with more than 15 distinct insertion sites. In *A. baumannii*, the proportion of intragenic insertions varies from approximately 30 % (IS*Aba*31 and IS*Aba*27) to over 70 % (IS*Aba*19 and IS*Aba*22). In *K. pneumoniae*, the intragenic proportion ranges less than 20 % (IS*Kpn*1) to over 80 % (IS*Kpn*18). A low proportion of intragenic insertions could be due to the fact that gene-disrupting insertions are more likely to be selected against than intergenic insertions. Alternatively, there may have been strong positive selection for certain intergenic events that has resulted in their high frequency.

Another indirect measure of the potential adaptive effects of IS insertion is the diversity of sites, which we calculated as the ‘diversity ratio’: the number of distinct sites divided by the total number of observed insertions in all genomes for each IS element. A high ratio means that most insertions are strain-specific, while a low ratio means that a few IS locations are shared by most strains carrying that element, with few additional strain-specific insertions. The latter group is more likely to represent positively selected insertions. In *A. baumannii,* the diversity ratio ranged from <10 % (IS*Aba*1, IS*Aba*17, IS26) to >75 % (IS*Aba*11, IS*Aba*31) (Table 1). As an example of a low diversity ratio, 190 of the 200 genomes that contain IS*Aba*24 have an insertion between the genes encoding hypothetical proteins M3Q_2649 and M3Q_2651 in TYTH-1. On the other hand, among the 29 genomes with IS*Aba*31, 72 of the 93 insertion sites are strain-specific. In *K. pneumoniae*, the abundant IS elements have diversity ratios less than 0.4, except IS*Kpn*14 and IS5.

Multiple independent insertions by the same or different IS elements in the same genomic region may also indicate that those insertions convey a selective advantage. In *K. pneumoniae*, there are fewer than two dozen genes or intergeneic regions with insertions by more than two different IS elements. In *A. baumannii*, however, there are 320 genes with three or more *different* IS elements inserted in them across the strain set (Table S5). An additional 185 intergenic locations have three or more *different* IS elements (Table S6). Among these, there is a strong bias for insertions between genes that are oriented so as to be up-regulated by an IS-encoded promoter. Only 18 (10 %) of the intergenic insertions are between genes oriented toward the IS insertion site; the remaining 89 % of insertion sites, a gene is oriented so as to be up-regulated by the adjacent IS element. One genome segment with multiple insertions is the four-gene region M3Q_2685–M3Q_2688 encoding the type I pilus proteins CsuA/B and their regulators, which has dozens of independent insertions by thirteen different IS elements. Twelve different IS elements were found in the 176 bp region between M3Q_2382 and M3Q_2383 in 389 strains. The repeated insertions at this locus suggest that these genes may encode important functions, although each encodes a hypothetical protein with no functionally characterized domains.

## Discussion

After correcting for the larger number of *A. baumannii* genomes in the analysis, there were about 40 % more total insertions observed in *A. baumannii* genomes than in *K. pneumoniae* genomes, and more than twice as many distinct insertion sites. Considering that *A. baumannii* genomes (~4 Mbp) are about 30 % smaller than *K. pneumoniae* genomes (~5.6 Mbp), the IS element density is even greater, with about one IS element every ~109 kbp in *A. baumannii*, compared with every ~185 kbp in *K. pneumoniae.* As can be seen in Table 3, there are many more IS locations that are shared in up to 10 % of the *A. baumannii* strains, while *K. pneumoniae* genomes have more sites that are shared by about half of the genomes, reflecting the large proportion of very similar ST258 strains in the dataset and the greater diversity of IS location patterns among the *A. baumannii* genomes.

There are several potential explanations for the more diverse set of IS locations in *A. baumannii* than in *K. pneumoniae*. The most straightforward may be that the *A. baumannii* strains represent a more diverse evolutionary history than the *K. pneumoniae* strains, and thus more time for IS elements to move and accumulate. There are more genomes on long branches of the *A. baumannii* phylogenetic tree than the *K. pneumoniae* tree. Although difficult to discern in Fig. 2, this is also true for the most abundant MLST groups: the sum of the branch lengths of GC2 *A. baumannii* strains is about four times longer than the sum of the ST258 *K. pneumoniae* strains. However, in both species, IS elements are more abundant in the recently emerged lineages than in the more diverse strains, so divergence time alone cannot explain the abundance differences. It appears that strong selection for a founder ST258 strain carrying the *bla*_KPC_ gene resulted in a rapid expansion of the this lineage (Bowers *et al.*, 2015) that contains a shared set of IS insertion sites that were present in the founder. Only the IS*Aba*1 site upstream of the *bla*_ADC_ gene is common to most GC2 strains, and it has been argued that this is due to multiple independent insertions, rather than a single shared ancestor (Hamidian & Hall, 2013).

Hawkey *et al*. (2015) have described a program that uses primary reads to identify the locations of insertion sites in *A. baumannii*. Their approach has some advantages over ours: by relying on primary sequence reads rather than assemblies, the impact of variation in sequencing and assembly methods and efficacy are reduced. However, publicly available read sets are much more difficult to work with than assemblies, requiring generally 50–150 times as much disk space and computing time during analysis. For example, it takes 20–120 min to download the reads from a single genome from SRA using the SRA Toolkit program fastq-dump. In the same length of time, contig sequences can be downloaded from 1000 genomes from the WGS division of GenBank and searched for IS content using ISseeker.

Other limitations to mapping IS locations in draft genomes include sequence variation in the IS element, differences between the query genome and the reference, and assembly artifacts that tend to occur near repetitive genome regions. Use of alternative run parameters resulted in differences in detection rate (Table S2), and suggest that for any particular insertion site, additional computational analysis may be needed to determine the insertion status in strains of interest.

## Supplementary Data

893 Supplementary File 1
