# Supplementary material for: Quantitative assessment of insertion sequence impact on bacterial genome architecture
Source: Microb Genom. 2016 Jul 18;2(7):e000062. doi: 10.1099/mgen.0.000062 (PMC5343135; doi:10.1099/mgen.0.000062)
Supplement: Supplementary file 1 [file mgen-02-62-s001.pdf]

Supplemental Figure 1. Example of a complex transposition event

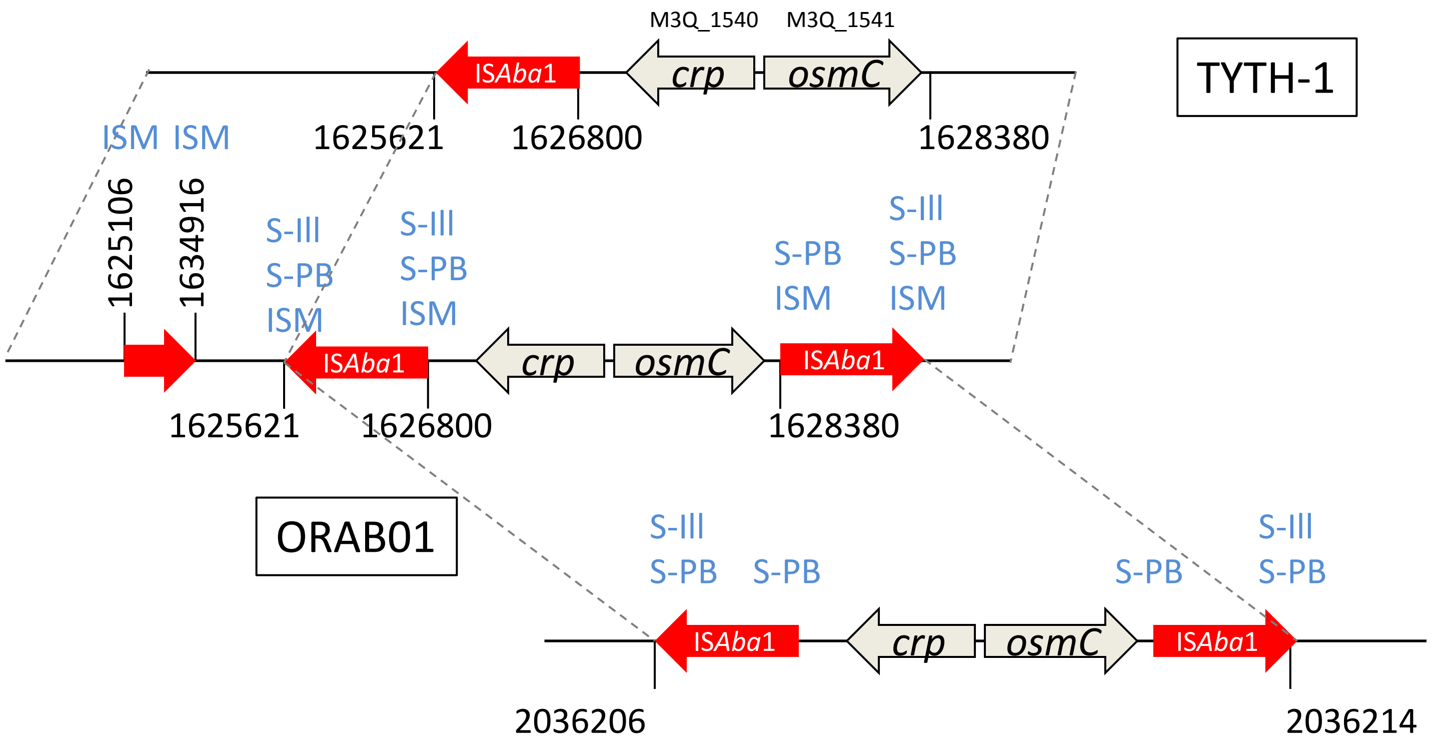

Supplemental Figure 2a

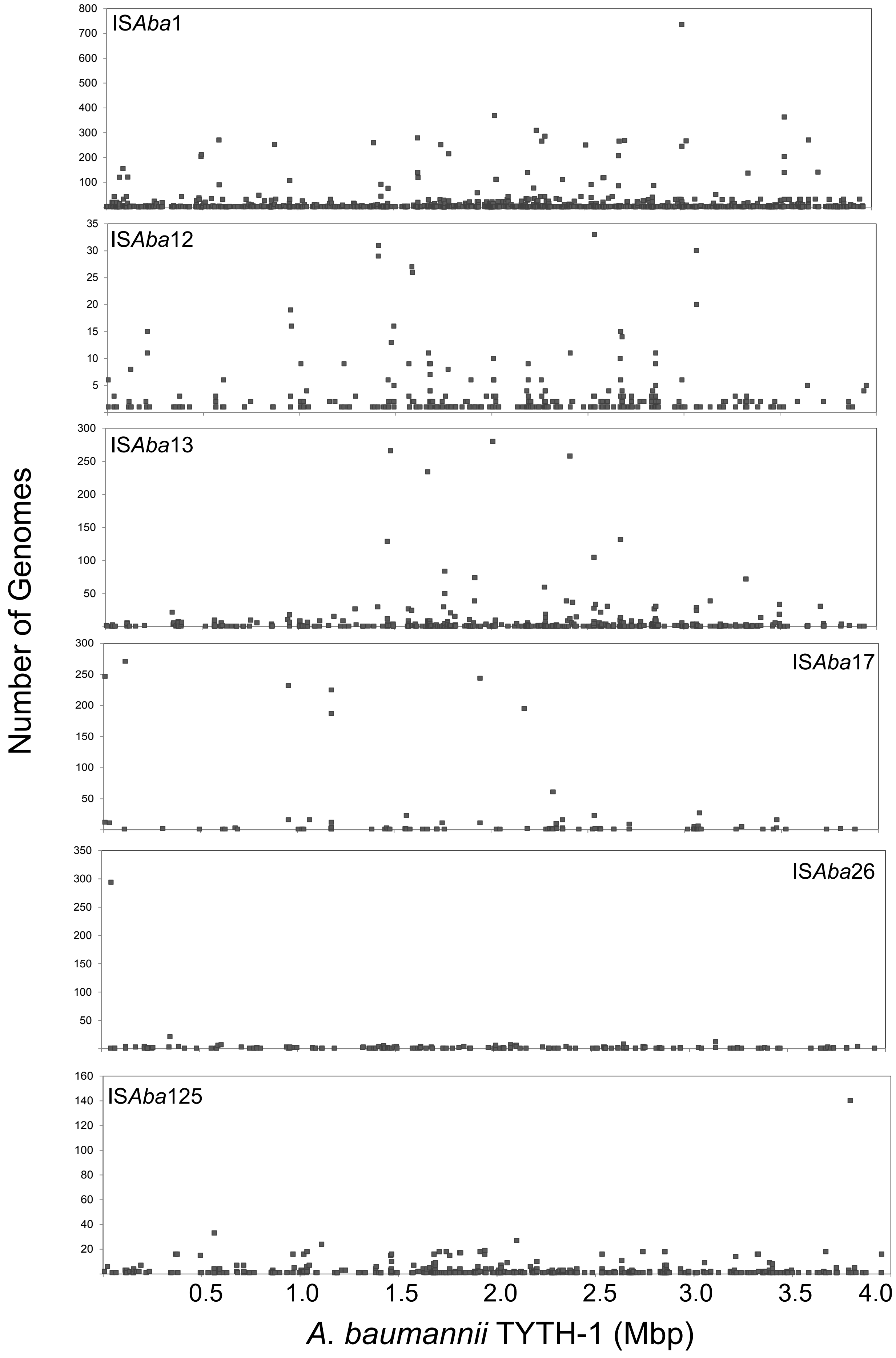

Supplemental Figure 2b

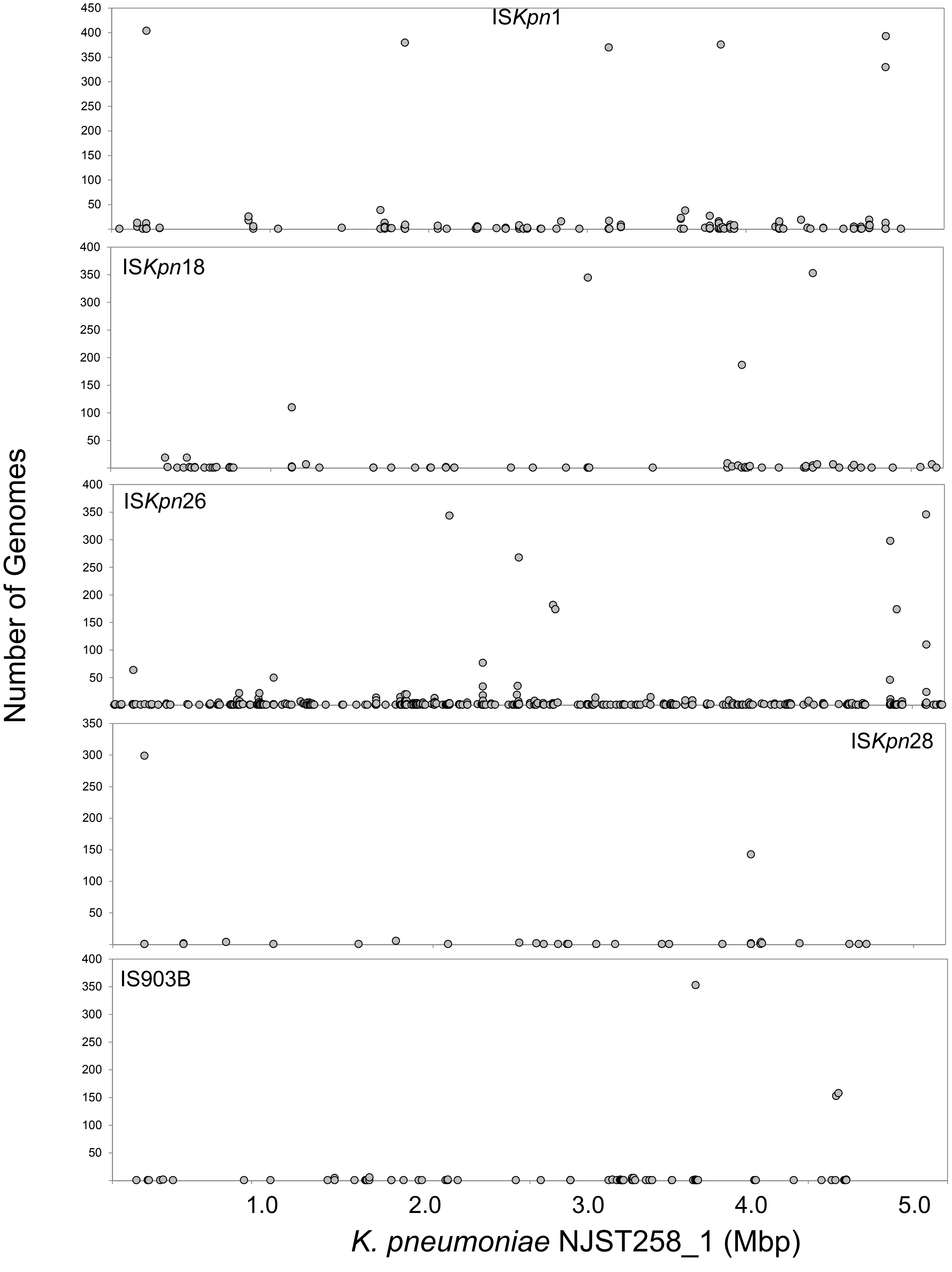

Supplemental Table 1. Comparison of ISseeker and ISmapper

Genome	IS element	Read lengtl	Mb in Reads
ORAB01	ISAba1	100	2754
AB5075-UW	ISAba1	250	7750
D36	ISAba1	100	1028
IOMTU 433	ISAba1	300	348
UHKPC05	ISKpn26	100	1187
KPR9028	ISKpn26	200	513
KPNIH10	ISKpn26	200	439
NJST258_1	ISKpn26	100	2829

		BLAST in Finished Genome	ISseeker - Finished Genome	ISseeker- Illumina Assembly	ISMapper - Reads	Present in reference	Non-standard structure	orien-tation	Reference Location
**ORAB01**	**ISAba1**	Y	Y	Y	Novel	N		R	34724
		Y	Y	Y	Novel	N		R	67290
		Y	Y	Y	Novel	N		R	110761
		Y	Y	Y	Known	Y		R	494100
		Y	Y	Y	Novel	N		F	796459
		Y	Y	Y	Novel	N		R	958283
		N	N	N	Novel?	N		F	1625106
		Y	Y	Y	Known	Y		R	1625620
		Y	Y	Y	Novel	N		F	1628371
		Y	Y	Y	Novel	N		R	1788677
		Y	Y	Y	None	N	Y	F/R	2036214
		Y	Y	Y	Known	Y		R	2247274
		Y	Y	Y	Known	Y		F	2291073
		Y	Y	Y	Novel	N		R	2384827
		Y	Y	Y	Novel	N		F	2595616
		Y	Y	Y	Novel	N		R	2600380
		Y	Y	Y	Known	Y		R	2675906
		Y	Y	Y	Novel	N		F	2837695
		Y	Y	Y	Known	Y		R	3007459
		Y	Y	Y	Novel	N		R	3543523
**AB5075-UW**	**ISAba1**								
		Y	Y	Y	Novel	N		R	1794598
		Y	Y	Y	Novel	N		F	3535712
**D36**	**ISAba1**								
		Y	Y	Y	Novel	N		R	226592
		Y	Y	Y	Novel	N		R	469972
		Y	Y	Y	Novel?	N	Y	F/R	482182
		Y	Y	Y	Novel	N		R	642099
		Y	Y	Y	Novel	N		F	766046
		Y	Y	Y	Novel	N		F	791110
		Y	Y	Y	Novel	N		F	1759986
		Y	Y	Y	Novel	N		R	1785142
		Y	Y	Y	Novel	N		R	1938677
		Y	Y	Y	Novel	N		F	2310208
		Y	Y	Y	Novel	N		R	2766531

		Partial	N	N	Novel?	N		F	2811080
		Y	Y	Y	Known	Y		R	3007457
		Y	Y	Y	Novel	N		F	3102099
		Y	Y	Y	Novel	N		F	3283542
		Y	Y	Y	Novel	N		R	3318119
**IOMTU 433**	**ISAba1**								
		Y	Y	Y	None	N		F	482460
		Y	Y	Y	None	N	Y	R	1447695
		Y	Y	Y	None	N		F	3479865
		Y	Y	Y	None	N		R	3723168
**UHKPC05**	**ISKpn26**								
		Y	Y	Y	Novel	N		R	775416
		Y	Y	Y	Known	Y		F	2102340
		Y	Y	Y	None	N	Y	R	2312687
		Y	Y	Y	None	N	Y	R	2530495
		Y	Y	Y	Novel	N		F	4597790
		Y	Y	Y	None	Y	Y	R	4861007
		Y	Y	Y	None	Y	Y	R	5085043
**KPR9028**	**ISKpn26**								
		Y	Y	Y	Novel	N		R	909160
		Y	Y	Y	Novel	N		R	1004339
		Y	Y	Y	None	N	Y	R	1227769
		Y	Y	Y	Novel*	Y		F	1898102
		Y	Y	Y	Known	Y		F	2102333
		Y	Y	Y	None	N	Y	R	2530495
		Y	Y	Y	None	N	Y	F	2752178
		Y	Y	Y	Novel	N		R	3335424
		Y	Y	Y	None	N	Y	F	4218305
		Y	Y	Y	Known	Y		R	4861020
		Y	Y	Y	None	Y	Y	R	5085043
**KPNIH10**	**ISKpn26**								
		Y	Y	Y	Novel*			F	126251
		Y	Y	Y	Novel*			R	787890
		Y	Y	Y	Novel*			F	1004411
		Y	Y	Y	None		Y	F	1825488
		Y	Y	Y	None	Y		F	2102327
		Y	Y	Y	None			R	2312684
		Y	Y	Y	None	Y		R	2537299
		Y	Y	Y	None	Y		R	4861007
		Y	Y	Y	None	Y	Y	R	5085043
**NJST258_1**	**ISKpn26**								
		Y	Y	Y	Known	Y		F	921273
		Y	Y	Y	Known	Y		F	2102345
		Y	Y	Y	Known	Y		R	2537321
		Y	Y	Y	Known	Y		F	2751005
		Y	Y	Y	Known	Y		R	4690392
		Y	Y	Y	Known	Y		R	4861029
		Y	Y	Y	Known	Y		R	5085064
		Total "Y"	74	74	56	Total Known + Novel			

20	None
2	False positive
54	True positive

	ISseeker	ISmapper
TP	74	54
FP	0	2
FN	0	20
Sensitivity	1	0.730

Supplemental Table 2. Results from ISseeker analysis using alternative run parameters

A. baumannii Reference	Parameters				Results				
	is_pct	flank_pct	flank_len	Location bundle	#distinct sites	#total	#genomes	%sites	%total
TYTH-1	97	97	100	1	1363	14526	823		
TYTH-1	95	95	100	1	1409	14765	825	103%	102%
TYTH-1	97	97	500	1	1209	12606	819	89%	87%
AB5075-UW	97	97	100	1	1206	13441	820	88%	93%
TYTH-1	97	97	100	5	1070	14526	823	79%	100%
TYTH-1	97	97	100	10	1038	14526	823	76%	100%

K. pneumoniae Reference	Parameters				Results				
	is_pct	flank_pct	flank_len	Location bundle	#distinct sites	#total	#genomes	%sites	%total
NJST258-1	97	97	100	1	687	4294	494		
NJST258-1	95	95	100	1	713	4373	497	104%	102%
NJST258-1	97	97	500	1	599	3940	480	87%	92%
NJST258-1	97	97	100	5	649	4294	494		
NJST258-1	97	97	100	10	622	4294	494		

Supplemental Table 3. IS copies in A. baumannii genomes

Genome	Accession*	Total	IS1396	IS26	ISAba1	ISAba2	ISAba5	ISAba6	ISAba7	ISAba8	ISAba9	ISAba10	ISAba11	ISAba12	ISAba13	ISAba14	ISAba16	ISAba17	ISAba18	ISAba19	ISAba20	ISAba21	ISAba22	ISAba24	ISAba25	ISAba26	ISAba27	ISAba28	ISAba29	ISAba3	ISAba31	ISAba32	ISAba33	ISAba36	ISAba125	ISAba825
1	JSAO00000000	3					1							1	1																					
269	JQNV00000000	22		1			2							2	1	1							1							1	1	4			8	
341	JQSD00000000	30		5	18										2								1	1		2							1			
1297	JEZJ00000000	13			4							7			1																				1	
1605	AUWL00000000	13		2	2		2							2	2											1							2			
2887	JFEP00000000	67		6	21										18		6	8							7										1	
3390	JFER00000000	11																							5	4					1				1	
3909	AEOZ00000000	4		1		1																			1					1						
3990	AEOY00000000	3		1																										1					1	
4190	AEPA00000000	4			1												2									1										
4749	JEXC00000000	28				2								1	2				2	2			1			5			1		12					
4857	AHAG00000000	17		2	3																		1												11	
5075	AHAH00000000	7			2										2											1									2	
5256	AHAI00000000	10			8								1	1																						
5711	AHAJ00000000	27		3	14															9			1													
6112	JMNY00000000	12												2	6											1	2				1					
6200	CP010397.1,CP0104	18														1																			17	
6935	JMHM00000000	31		4	19									1												1								5	1	
7893	JFDK00000000	74		5	23										18		1	11							1										15	
10519	JFFF00000000	83		2	28										15		14	5							17										2	
11126	JFWS00000000	39		3	20							4											1			1									10	
14216	JFXN00000000	15		4											1																			10		
15827	JMNX01	61		6	29									1	16			6															1		2	
17534	JEYQ00000000	45		2	22										9		1	7							2										2	
18689	JMNI00000000	49		4	19									1	8		4	7							4										2	
21072	JMOD00000000	54		1	1	1	1							3	13	1				2			4			15			1	1	10					
21871	JEWV00000000	13													1							3	1			6					2					
23037	JMOC00000000	50		3	21										11		3	6							4										2	
23671	JFEN00000000	59		4	26									5												1								22	1	
29280	JEZI00000000	19		4	13																														2	
34001	JEZK00000000	36		2	21										5			7																	1	
34654	JEZL00000000	44		5	23										6			10																		
42887	JEYG00000000	25		5	20																															
43926	JEXW00000000	29		4	20																				2										3	
48055	AOSP00000000	30		3	23																		1			1									2	
50595	JEXP00000000	18															5								5	4					1				3	
53264	ALPW00000000	30		3	23																		1			1									2	
58452	JEZV00000000	38		4	22									4												1								6	1	
70136	JFYK00000000	34		3	22																		1			1									7	
72431	JMNZ00000000	11			1		1							1						1			2						1		2				2	
73736	JFFE00000000	48		4	26										10			7																	1	
83444	JEYC00000000	144			4	8							21	31	16					12			2			10	2		6	1	6	1	1		23	
88816	JFYG00000000	13													2			1					5			2	1						1		1	
96512	JMNK00000000	99		4	27									2	13		19	10							22										2	
98826	JFYJ00000000	51		6	19										10			10																	6	
118362	JEWB00000000	3					3																													
121738	JFDB00000000	13																					7			5					1					
135867	JMNP00000000	58		3	23										13		3	9							5										2	
136706	JEZH00000000	52		5	23										11		1	7							4										1	
144107	JEYE00000000	3																								3										
145660	JFXU00000000	38		3	20							4											1			1									9	
146457	JEWS00000000																																			
213697	JFYE00000000	55		5	26										11			9							1										3	
214216	JFXR00000000	15		4											1																			10		
216872	JFEM00000000	14			3									2	1								5			1	2									
225588	JFXV00000000	24					1								1																1				21	
230853	JEWW00000000	9																													2	1			6	
232184	JEYI00000000	7			3																														4	
233846	JMOG00000000	7			2								1	3	1																					
259052	JEYJ00000000	9													1											4	3				1					
268680	JEYN00000000	4																																	4	
272263	JEWX00000000	4												1								1									1	1				
273929	JFEO00000000	72		4	18									3									1			1								45		
277047	JFXS00000000																																			
299505	JEWY00000000																																			
303002	JFXW00000000	54		4	26										14			8																	2	
318814	JFEQ00000000	1												1																						
323408	JFCZ00000000	42		3	21							3											1			1									13	
339786	JFXO00000000	40		3	20							3											1			1									12	
342950	JEWZ00000000																																			
348935	JEVW00000000	36											3	2		3				2							2		2		12				10	
397971	JEYD00000000	1											1																							
400834	JEXA00000000	84		2	24		1							1	15		16	5							18										2	
426863	JFYF00000000																																			

457946	JFET00000000	34	3						3														28	
458282	JFCY00000000	54	5	25					12	1	8				1									2
466215	JFXP00000000	69	4	28					13	5	5				10									4
466760	JEXB00000000	2														2								
470922	JFCX00000000	90	5	28					11	12	9				24									1
478810	JFFH00000000	33			1				1			2				2		1						26
479375	JEZN00000000	27			1	17		2	1	3	2								1					
480175	JFEU00000000	26	10	13																				3
496487	JMNR00000000	18	2						1														15	
532279	JEYH00000000																							
532413	JEZO00000000	38	4	19				5								1							8	1
541915	JFXQ00000000																							
554515	JEXT00000000	49	1	17				1	17		10											1		2
562700	JEZP00000000	19	3	16																				
564012	JEZQ00000000	50	5	22					11	2	7				2									1
573719	JFYA00000000	14						4	1	1	6			1	1									
607805	JFES00000000	24	3						1														19	1
625974	JEXD00000000	16		3					2			1		1		2				5	2			
628418	JFXX00000000	74	4	26					12	3	8				20									1
647609	JEXE00000000	51	3	21					14		8													5
653020	JEZU00000000	36	2	24					3		7													
655555	JFEW00000000	79	3	23				3	14	13	8				13									2
694762	JFEV00000000	39		2		1		1	1			3		4		4	1			3	3			16
722310	JFCV00000000	17	3						1														13	
723929	JFEX00000000	26			1				1											1				23
724909	JEXF00000000	49	3	24					12		8													2
730795	JFYB00000000	41	3	20		3								1		1								13
737393	JFEY00000000	52	4	23					13		6				5									1
742879	JFYC00000000																							
754286	JMNU00000000	15	2						1														12	
755829	JFXY00000000	88	6	24					14	13	8				22									1
756476	JMOR00000000	57	4	24				1	15	1	9				1									2
766875	JEXX00000000	5														1	3			1				
781407	JEZS00000000	1																		1				
809848	JEXG00000000																							
825610	JFEZ00000000	27	4	20										1		1								1
826659	JFYD00000000																							
831240	JEYO00000000	2												1						1				
836190	JFFA00000000	59	6	20					17	1	7				7									1
846928	JFFD00000000																							
855125	JMNT00000000	2						1						1										
869535	JEXH00000000	10						1					1											8
883425	JFXZ00000000	2			1				1															
907131	JEXS00000000	3							1				1	1										
916567	JMNJ00000000	61	5	26				1	14	3	7				4									1
923118	JFFB00000000	75	4	18					19	4	11				4									15
940793	JMNW00000000	26	9	14																				3
942133	JEZX00000000	57	6	21					9	6	8				6									1
942194	JFAC00000000	20	3	17																				
947299	JEZY00000000	53	5	21					10	4	7				4									2
951631	JEXI00000000	16	3						1														12	
959073	JEZZ00000000	47	6	20					10	1	8				1									1
972082	JFAA00000000	6	1	1					1															3
981176	JFYH00000000	114	2	26					13	30	6				36									1
983759	JEXO00000000	9		1			1	1																6
984213	JEVX00000000	24			2			8	1															13
987421	JMNL00000000	48	2	25				1	9	1	7				2									1
993520	JFAB00000000	51	5	22				1	9	2	7				4									1
1007214	JFWQ00000000	18								4					7	4				1				2
1022959	JFDQ00000000	102	7	26					13	20	8				27									1
1031433	JEYR00000000	54	6	21					8	3	7				7									2
1032241	JEYS00000000	19	3	15		1																		
1032359	MHK01	61	6	25				1	15	2	7				4									1
1035119	JEVY00000000																							
1036938	JEYT00000000	34	4	18				3								1							7	1
1039621	JEYU00000000	23	3	20																				
1040094	JEYV00000000	23	3	20																				
1043794	JEYX00000000	3																						3
1043903	JEYY00000000	54	3	27				1	10	1	8				2							1		1
1045092	JFFC00000000	64	4	27					13	6	6				7									1
1046051	JEYZ00000000	32	4	19				1								1							6	1
1046674	JEZA00000000	49	4	22					11	1	7				3									1
1051176	JEZB00000000	27	1	24										1		1								
1051830	JEZC00000000	38	5	18					6		7													2
1058283	JFWR00000000	43	2	18		11				5					6									1
1062314	JFDR00000000	5																	2	3				
1075025	JEZD00000000	25	5	20																				
1095464	JFDT00000000	47	3	22					9		7													6

1096934	JEXM00000000																								
1106579	JEXN00000000	117		1	1			27	17				1	10				9	2	9	6				34
1117819	JFWW00000000	46	3	22					9			6													6
1130196	JFWX00000000	6				2			1									1	1						1
1146103	JMNS00000000	35	5	23												1		1						4	1
1159076	JEWA00000000	96	5	25				2	19		16	8					19						1		1
1170863	JFWT00000000	17	4						1															12	
1178044	JFDU00000000	45	2	26					9			7													1
1179249	JFXC00000000	21							1					5				2	6	3	4				
1188188	JFDV00000000	25	6	15																					4
1202252	JEWC00000000	36	4	18			8										4								2
1207552	JFEC00000000	51	3	23				3										1						20	1
1232509	JFWY00000000	46	6	21					11			5													3
1237202	JFWU00000000	50	6	24				1	8			9													2
1237893	JFEA00000000	26	9	14																					3
1239920	JEXZ00000000	1																				1			
1245249	JFDY00000000	8							1						2	1		2			1				1
1245593	JFDW00000000	8						2	1							2		2					1		
1247182	JEZF00000000	37	4	21				2										1						8	1
1264765	JMNG00000000	15																14			1				
1264936	JFED00000000	47	4	24					10			6													3
1266220	JFXA00000000	70	6	24				1	11		7	8					12								1
1267820	JEWD00000000	6		5								1													
1271213	JFDX00000000	24	5	16																					3
1271651	JFDZ00000000	49	4	27					9			7													2
1277411	JEXL00000000	30	4	22												1		2					1		
1281984	JFXD00000000	2																1			1				
1284800	JMOB00000000	86	4	27				1	15		13	7					16								3
1287985	JFFG00000000	51	6	23					9			7													6
1289546	JEZG00000000	88	6	23					13		17	8					20								1
1289694	JEWE00000000	31		6				2							1	3					1				18
1291820	JEXK00000000	28	5	20												1		2							
1293320	JFEE00000000	1								1															
1294217	JEWF00000000	83	7	26					16		13	6					14								1
1294222	JFXF00000000	104	6	24					14		23	8					28								1
1294243	JFXG00000000	1							1																
1294596	JFCC00000000	14																				1			13
1295259	JEWG00000000	39		7			1		1							4		3	1				1		21
1295549	JFXB00000000																								
1295743	JEWH00000000	39	1						16									15			7				
1296252	JFFU00000000	96	6	22					13		20	7					27								1
1297549	JEVV00000000	28							1																27
1391434	JFXH00000000	14							14																
1392509	JFXJ00000000	47	6	23					10			5													3
1396970	JEWI00000000	25		1	2	1	1		1			4		1	3			2	1		6		2		
1397084	JEWJ00000000	94	4	24				1	14		19	7					24								1
1397513	JFXI00000000	45	6	21					9			5													4
1406182	JFXK00000000	45	2	22					6			8													7
1406589	JFYI00000000	32	8	17					2																5
1406750	JEWK00000000	107	6	24				2	14		23	8					29								1
1412924	JFEF00000000																								
1413735	JFWZ00000000	13	3						1															9	
1417041	JEYP00000000																								
1419130	JEWL00000000	10			3			2										1		3	1				
1424608	JFXL00000000	14							1						3	2		6			2				
1426993	JFCU00000000	67	1	25					16		2	5					15								3
1428368	JFXM00000000	37	10	22												1		1							3
1429530	JEWM00000000	3				2		1																	
1437282	JEWN00000000																								
1440422	JEWO00000000	113			3	1		1	23				3	18		6		14	15	17	7				5
1440750	JEXQ00000000	31	1	28												1		1							
1457504	JFEG00000000	47	10	21					9			6													1
1461402	JEWP00000000	11																				1			10
1461963	JEWQ00000000	6			1			1	1				1	1						1					
1462234	JEWR00000000	59	5	33					7			6											1		7
1465485	JEWT00000000	116	6	23					15		29	8					33								2
1471012	JFEH00000000	53	4	26					13			8													2
1475718	JEWU00000000	27			1	1		1	1					1							1				21
1475764	JFEJ00000000	50	10	23				1	9			6													1
1482820	JFEK00000000	50	10	24					9			6													1
1488685	JFCT00000000	38	3	20			3									1		1							10
1494580	JFXT00000000	75	5	22					18			11					4								15
1499986	JMOA00000000	19	2						1															16	
1505311	JMOL00000000	61	5	21					14			5					1				3			9	3
1525283	JEXR00000000	3				1		1													1				
1526966	JEXV00000000	40	4	20				2										2						11	1
1533268	JFEI00000000																								
1539026	JMNH00000000	28	4	22												1		1							

1542444	JEYA00000000																												
1546444	JEXU00000000																												
1552389	JFCS00000000	39	3	20				3											1			1							11
1552818	JFCD00000000	109	2	25							13		29	7							32								1
1552865	JEXY00000000	39	3	21				3											1			1							10
1564232	JFEL00000000	8									1							2	1			2			1				1
1566109	JEYB00000000	40			4					2				1	2	8		3			2	11	2	5					
1571545	JMOM00000000	13								2								4	1				4		2				
1575710	JEYM00000000	85	2	26							14		16	5							19								3
1578804	JEYF00000000	25						8			1								1			14							1
1593273	JEYL00000000	58	2	24						1	15			7													1		8
1598530	JMOE00000000																												
6013113	ACYR00000000	32	1	12		1	1		1	1		3							1								11		
6013150	ACYQ00000000	33	1	12		1	1		1	1		3							1							1	11		
6014059	ACYS00000000	46	2	15									5						2		5	1					14		2
6870155	JRWO00000000	7	1	3							2											1							
1042969-11	JMOH00000000	39	2	17						1	9			8															2
1042969-12	JEYW00000000	34	2	17							8			7															
1064293_45	JFDS00000000	4		1																									3
1064293_46	JMHL00000000	47	7	21							10			7															2
107m	CBSG00000000			18	2											8							2						
1195185_80	JMOI00000000	18	3								1																	14	
1262761-10	JMOJ00000000	11																			1	5			1				4
1262761-97	JFWV00000000	42	6	19							9			5															3
1276470-13	JMOK00000000	40	3	20				4											1			1							11
1276470-86	JFXE00000000	22	5	15																									2
16553_1	JHPM00000000	42	5	18							7			10															2
16553_10	JHPF00000000	26	5	16				4			1																		
16553_2	JHPL00000000	29	6	17				5			1																		
16553_3	JHPK00000000	26	2	19				4			1																		
16553_4	JHPJ00000000	28	7	16				4			1																		
16553_6	JHPI00000000	28	6	17				4			1																		
16553_8	JHPH00000000	28	7	16				4			1																		
16553_9	JHPG00000000	29	5	19				4			1																		
1656-2	CP001921,CP00192	45	7	15				21				2																	
1999BJAB11	JSDB00000000	18	2	9						1		4							1						1				
2003BJAB12	JSCK00000000	22	7	12															1	1		1							
2004BJAB10	JSCJ00000000	23	8	12															1	1		1							
2004BJAB13	JSCL00000000	23	5	15															1	1		1							
2004BJAB14	JSCM00000000	21	6	12															1	1		1							
2004ZJAB5	JSCG00000000	19	5	13																1									
2004ZJAB6	JSCX00000000	14		10													1												3
2005JSAB1	JSCO00000000	12	2	8															1	1									
2005LNAB4	JSCG00000000	19	5	13																1									
2011BJAB1	JSCG00000000	19	5	13																1									
2011BJAB2	JRHW00000000	25	6	17															1			1							
2011BJAB3	JSCG00000000	19	5	13																1									
2011BJAB4	JSCH00000000	18	5	10															1	1		1							
2011BJAB5	JSCY00000000	25	6	17															1			1							
2011BJAB6	JSDM00000000	26	6	17															1			2							
2011BJAB7	JSCI00000000	24	5	16															1	1		1							
2011BJAB8	JSCZ00000000	32	8	20															1	1		2							
2011BJAB9	JSDA00000000	4									2											1	1						
2011GDAB1	JSDC00000000	32	6	18							6									1		1							
2011GDAB2	JSDD00000000																												
2011GDAB3	JSCN00000000	32	6	18							5								1	1		1							
2011GDAB4	JSDE00000000	15	1	10															1	1		2							
2011HNAB1	JSDF00000000	19	3	13															1	1		1							
2011LNAB1	JSCT00000000	16	4	10															1			1							
2011LNAB2	JSCG00000000	19	5	13																1									
2011LNAB3	JSDI00000000	21	5	12														1	1	1		1							
2011SDAB1	JSCG00000000	19	5	13																1									
2011SDAB2	JSCG00000000	19	5	13																1									
2011SDAB3	JSCS00000000	26	6	17															1	1		1							
2011SHAB1	JSCP00000000	32	8	21															1	1		1							
2011TJAB1	JSDG00000000	57			6	1				3	4				4	6		10	15			3		4	1				
2011ZJAB1	JSCV00000000	30	3	13								2	3				2		1	2	3	1							
2011ZJAB2	JSDJ00000000	45	4	12								2	8						1	2	7	1					8		
2011ZJAB3	JSDK00000000	27	4	21															1			1							
2011ZJAB4	JSDL00000000	11		5																		5			1				
24812_10	JFDJ00000000	35	2	16							8			8															1
24812_2	JMNN00000000	37	2	18							9			8															
24812_4	JFDI00000000	38	2	16							9			9															2
24812_6	JFDH00000000	39	2	19							9			8															1
24812_7	JMOU00000000	36	2	17							9			8															
24812_8	JMON00000000	35	1	19							8			7															
24845_1	JFWM00000000	46	4	22							9			9															2
24845_10	JFWN00000000	41	4	17							9			9															2

24845_3	JFWL00000000	47	4	23			9		9			2
24845_4	JFWK00000000	44	4	21			9		8			2
24845_5	JFWJ00000000	43	4	19			9		9			2
24845_6	JFWI00000000	42	2	20			9		9			2
24845_7	JFWH00000000	44	4	20			9		9			2
24845_8	JFWG00000000	43	4	19			9		9			2
24845_9	JFWF00000000	50	3	20	1	2	12		9		1	2
24860_1	JIDG00000000	36	2	17			9		8			
24860_10	JFDG00000000	40	1	17		4	10		8			
24860_2	JFDF00000000	37	2	18			9		8			
24860_3	JMOO00000000	34	2	15			9		8			
24860_5	JMOP00000000	36	2	17			9		8			
24860_6	JIDF00000000	36	2	17			9		8			
24860_7	JFDD00000000	36	2	16			9		9			
24860_8	JFDC00000000	38	2	20			8		8			
24860_9	JMOQ00000000	35	2	16			9		8			
24975_1	JGAI00000000	39	1	18		1	10		8	1		
24975_10	JIDE00000000	36	2	17			9		8			
24975_4	JGAH00000000	39	2	20			9		8			
24975_5	JGAG00000000	37	1	16		1	11		7			1
24975_7	JGAF00000000	33	2	15			8		8			
24975_9	JGAE00000000	35	2	17			8		8			
25253_1	JFWD00000000	36	2	18			8		8			
25253_10	JFWE00000000	36	2	18			8		8			
25253_3	JFWC00000000	34	2	16			8		8			
25253_4	JFWB00000000	36	2	18			8		8			
25253_5	JFWA00000000	34	1	17			8		8			
25253_6	JFVZ00000000	35	2	18			8		7			
25253_7	JFVY00000000	37	2	16			8		8	1		2
25253_8	JFVX00000000	36	2	18			8		8			
25307_1	JFWO00000000	35	2	17			8		8			
25307_2	JFVW00000000	33	1	14		1	9		8			
25307_3	JFVV00000000	35	2	17			8		8			
25307_4	JFVU00000000	34	2	16			8		8			
25307_5	JFVT00000000	36	2	18			8		8			
25307_6	JFVS00000000	35	2	17			8		8			
25307_8	JFVR00000000	33	2	15			8		8			
25442_1	JMHI00000000	50	5	23			8	3	7	3		1
25442_5	JICS00000000	42	4	18			9		9			2
25442_7	JICR00000000	43	5	22			8		7			1
25442_9	JICQ00000000	50	5	23			8	2	7	4		1
25493_1	JHDD00000000	18	5	13								
25493_10	JHDE00000000	17	4	13								
25493_2	JHDC00000000	18	5	13								
25493_3	JHDB00000000	21	3	18								
25493_4	JHDA00000000	18	5	13								
25493_5	JHCZ00000000	18	5	13								
25493_6	JHCY00000000	22	4	17					1			
25493_8	JHCX00000000	20	3	17								
25493_9	JHCW00000000	19	5	14								
25561_1	JGAD00000000	52	5	25			8	2	7	4		1
25561_10	JGBG00000000	52	5	23			8	3	7	5		1
25561_2	JGAC00000000	56	5	25			8	5	7	5		1
25561_7	JGAB00000000	55	5	25			8	4	7	5		1
25569_1	JHPE00000000	19	3	16								
25569_10	JMNO00000000	20	4	16								
25569_2	JHPD00000000	24	6	18								
25569_3	JHOW00000000	22	4	18								
25569_4	JHPC00000000	23	6	17								
25569_5	JHOR00000000	21	5	16								
25569_6	JHOT00000000	56	5	23			12	3	6	6		1
25569_7	JHOV00000000	21	6	15								
25569_8	JICU00000000	19	3	16								
25569_9	JICT00000000	20	4	16								
25681_2	JGCI00000000	57	5	22			12	5	5	7		1
25681_3	JGCH00000000	59	4	22			12	7	6	7		1
25681_5	JGCG00000000	59	4	22			12	7	6	7		1
25681_6	JGCF00000000	60	5	24			12	6	6	6		1
25681_8	JGCE00000000	56	4	22			12	5	6	6		1
25681_9	JGCD00000000	57	5	21			12	5	6	7		1
25691_1	JGAW00000000	51	6	22			8	3	7	4		1
25691_10	JGAS00000000	50	5	22			8	2	7	5		1
25691_2	JGAV00000000	44	4	18			8	3	6	4		1
25691_5	JMOV00000000	48	2	23			9	3	6	4		1
25691_6	JGAU00000000	49	5	25			8		7	3		1
25691_7	JMPH00000000	51	5	22		2	8	3	6	3	1	1
25691_8	JMOW01	54	6	25			8	4	5	4	1	1
25691_9	JGAT00000000	51	5	22			8	3	7	5		1

25750_1	JMOX00000000	52	4	23				1	8	4	6					5							1
25750_6	JGCC00000000	48	4	23					8	2	7					3							1
25750_7	JGCB00000000	52	5	24					8	3	7					4							1
25750_8	JGCA00000000	51	4	23					9	3	7					4							1
25766_1	JGBF00000000	56	3	22					14	4	5					7							1
25766_10	JGAX00000000	56	5	22					12	3	6					7							1
25766_2	JGBE00000000	61	5	24					12	6	6					6							2
25766_3	JGBD00000000	49	5	21					12		4					5							2
25766_4	JGBC00000000	61	5	24					12	5	6					7							2
25766_5	JGBB00000000	58	4	22					12	6	6					7							1
25766_6	JGBA00000000	61	5	24					12	5	6					7							2
25766_7	JGAZ00000000	60	5	21					12	7	6					7							2
25766_9	JGAY00000000	55	5	22					12	3	5					7							1
25878_1	JHCV00000000	48	5	22					12		6					2							1
25878_2	JHCU00000000	45	5	21					11		6					1							1
25878_3	JHCT00000000	47	5	21					12		6					2							1
25935_1	JGAR00000000	50	5	22					8	3	7					4							1
25935_10	JGAJ00000000	54	5	23					9	4	7					5							1
25935_2	JGAQ00000000	52	5	23					8	3	7					5							1
25935_4	JGAP00000000	47	5	22					8	2	6					3							1
25935_5	JGAO00000000	49	4	22					8	3	7					4							1
25935_6	JGAN00000000	48	5	23					8	2	5					4							1
25935_7	JGAM00000000	51	5	23					8	3	7					4							1
25935_8	JGAL00000000	45	5	21					8	2	6					2							1
25935_9	JGAK00000000	50	4	23					8	3	7					4							1
25977_1	JFVP00000000	23					2		1				1		1	2	6	5	1	1		1	2
25977_10	JFVQ00000000	27			2		2		2			1	2		1		5	6	2	1		1	2
25977_2	JFVO00000000	26					2		2				2		1	2	6	5	2	1		1	2
25977_3	JFVN00000000	30			1		2		3			1	2		3	1	6	5	2	1		1	2
25977_4	JFVM00000000	25			1		2		1	1			1		1	1	6	6	1	1		1	2
25977_6	JFVL00000000	26					2		2				2		1		6	5	2	1		3	2
25977_7	JFVK00000000	27			1		2		1			1	2		1	2	6	5	2	1		1	2
25977_8	JFVJ00000000	26					2		1	1			2		1	2	6	5	2	1		1	2
25977_9	JMPF00000000	27			4		2		1			3	2		1		6	3	1	1		1	2
26016_1	JHOY00000000	56	5	23					14	1	6					6							1
26016_10	JHOU00000000	56	5	24					12	4	5					5							1
26016_2	JMOY00000000	58	3	26				2	14	1	7					3						1	1
26016_3	JMOZ00000000	49	3	21					12	1	6					5							1
26016_4	JHPB00000000	53	5	24					12		6					5							1
26016_5	JHOS00000000	52	5	23					12		6					5							1
26016_6	JHOX00000000	52	5	22					12	1	6					5							1
26016_7	JMPA00000000	61	3	26				1	13	4	7					6							1
26016_8	JHOO00000000	54	5	21					12	1	6					8							1
26016_9	JIDC00000000	55	5	25					12		6					6							1
263903-1	JMNM00000000	23								2				1		2					1		17
263903-2	JFDA00000000	11				1			1								6			1			2
42057_2	JICP00000000	48	3	21					10		8												6
42057_3	JMPB00000000	24	3	19																			2
42057_4	JMHJ00000000	23	5	16																			2
42057_5	JMPC00000000	23	3	17																			3
42057_6	JICO00000000	23	5	16																			2
43557_1	JHCS00000000	66	4	25				1	14	11	6					2							3
44298_1	JIDB00000000	53	6	19					10		10												8
44298_10	JICV00000000	54	6	20					10		10												8
44298_2	JIDA00000000	56	6	22					10		10												8
44298_3	JMHA00000000	53	6	20					10		9												8
44298_4	JICZ00000000	54	5	21					10		10												8
44298_5	JMHB00000000	55	6	21					10		10												8
44298_6	JICY00000000	52	6	18					10		10												8
44298_7	JICX00000000	50	4	18					10		10												8
44298_8	JMHC00000000	53	5	19					10		10												9
44298_9	JICW00000000	55	6	21					10		10												8
44327_1	JGBZ00000000	70	3	23					12	12	5					12							3
44327_2	JGBY00000000	79	3	26					12	14	7					14							3
44327_4	JGBX00000000	73	3	22				1	12	13	5					14							3
44327_5	JGBW00000000	74	4	23					14	12	5					13							3
44327_6	JGBH00000000	73	4	28				1	13	9	4					12							2
44327_7	JGBV00000000	75	3	23					13	14	5					15							2
44327_8	JGBU00000000	75	4	23					13	12	6					13							4
44362_1	JHCQ00000000	81	4	26				1	15	12	5					14							4
44362_10	JHCR00000000	56	4	27					13	1	5					2							4
44362_2	JHCP00000000	79	4	24					13	14	5					15							4
44362_3	JHCO00000000	72	3	23					13	11	6					12						1	3
44362_4	JHCN00000000	77	4	26					13	12	5					13							4
44362_5	JHCM00000000	78	4	26					13	13	5					14							3
44362_6	JHCL00000000	84	3	30					13	13	6					15							4
44362_7	JHCK00000000	76	4	24					14	12	5					13							4
44362_8	JHCJ00000000	50	4	24					14		5												3

44362_9	JHCI00000000	77	4	24							13	13	5					14								4	
44437_3	JHCH00000000	52	6	18							10		10													8	
44437_4	JHCG00000000	53	6	19							10		10													8	
44437_5	JHCF00000000	55	6	21							10		10													8	
44437_7	JHCE00000000	54	6	20							10		10													8	
44437_8	JHCD00000000	53	6	19							10		10													8	
44437_9	JHCC00000000	53	6	17							11		10													9	
44467_1	JGBS00000000	80	4	26							13	13	6					14								4	
44467_10	JGBT00000000	81	4	27							13	13	6					14								4	
44467_2	JGBR00000000	80	4	25							13	14	5					15								4	
44467_3	JGBQ00000000	79	3	28							12	13	6					13								4	
44467_4	JGBP00000000	80	4	27							13	13	5					14								4	
44467_5	JGBO00000000	75	4	26						1	13	10	5					12								4	
44467_6	JGBN00000000	78	4	24							13	13	6					14								4	
44467_7	JGBM00000000	77	4	25							13	12	6					13								4	
44467_8	JGBL00000000	76	3	25							13	12	6					13								4	
44467_9	JGBK00000000	82	4	26							13	14	5					16								4	
44839_10	JIDD00000000	49	5	19							10		9													6	
44839_8	JMHD00000000	49	5	18							10		10													6	
44839_9	JMHE00000000	53	5	23							9		10													6	
44857_10	JGBJ00000000	50	6	19							9		10													6	
44857_9	JGBI00000000	49	5	19							9		10													6	
44895_1	JFVH00000000	47	4	18							9		10													6	
44895_10	JFVI00000000	49	6	18							9		10													6	
44895_2	JFVG00000000	52	6	20							9		10													7	
44895_3	JFVF00000000	50	5	20							9		10													6	
44895_4	JFVE00000000	48	2	20							10		10													6	
44895_5	JFVD00000000	50	6	18							9		10													7	
44895_6	JFVC00000000	50	6	18							10		10													6	
44895_8	JFVB00000000	49	6	19							10		8													6	
44895_9	JFVA00000000	52	6	19							9		10													8	
45002_10	JFUZ00000000	39	1	20				5								1			1							11	
45002_3	JFUY00000000	40	3	20				5								1			1							10	
45002_4	JFUX00000000	40	3	20				5								1			1							10	
45002_5	JFUW00000000	40	3	20				5								1			1							10	
45002_6	JFUV00000000	40	3	20				5								1			1							10	
45002_7	JFUU00000000	40	3	20				5								1			1							10	
45002_8	JFUT00000000	42	3	22				5								1			1							10	
45002_9	JMPG00000000	48	3	24				5			1		3			1			1							10	
45052_1	JHOQ00000000	20	5	15																							
45052_2	JMPD00000000	20	5	15																							
45052_3	JHPA00000000	20	5	15																							
45052_4	JICN00000000	20	5	15																							
45052_5	JHOZ00000000	20	4	16																							
45057_1	JMPE00000000	38	1	20				5								1			1							10	
45057_4	JHOP00000000	49	5	17							9		10													8	
45075_1	JMHF00000000	21	5	16																							
45075_10	JMNV00000000	19	4	15																							
45075_2	JMHG00000000	20	4	16																							
45075_3	JMHH00000000	19	4	15																							
45075_4	JMND00000000	24	5	16																						3	
45075_5	JMHN00000000	21	5	16																							
45075_6	JMHO00000000	20	5	15																							
45075_8	JMNE00000000	22	5	17																							
45075_9	JMNF00000000	21	5	16																							
472237-1196	JEZM00000000	43	3	19							10		9													2	
472237-120	JFCW00000000	25	6	16																						3	
662545-1347	JEZR00000000	43	5	18							7		12													1	
796380-1375	JMOS00000000	20							2							1		2	6	5			1	1		2	
796380-2102	JEZT00000000	61	5	21						1	12	7	6					8								1	
929679-2095	EZW00000000	58	6	21							12	4	6					8								1	
929679-598	JMOT01	54	9	23				5		1	5		8													3	
A1	CP010781.1,CP0107	1	1																								
A144	JQSF00000000	9	3																							6	
A33405	JPXZ00000000	18	1	8							2												1			6	
AA-014	AMGA00000000																										
AB_1536-8	AMHA00000000																										
AB_1582-8	AMHB00000000	25	3	15												1		1	1						4		
AB_1583-8	AMHC00000000	21	3	2			1			3	3					1							1			6	1
AB_1594-8	AMHD00000000	29	3	16																						10	
AB_1595-8	AMHE00000000	34	4	26												1			2							1	
AB_1649-8	AMHF00000000																										
AB_1650-8	AMHG00000000																										
AB_1766_8	AMJO00000000	24	3	18												1	1		1								
AB_2007-09-	AMHH00000000	221		5	19	20			26	31	28		1	19	19	1		3	12	3	19	7	2	5		1	
AB_2007-16-	AMHI00000000	20			5									5	5						5						
AB_2007-16-	AMHJ00000000	20			5									5	5						5						
AB_2008-15-	AMHK00000000	48	4	18						7	7	3	5					3								1	

AB_2008-15-	AMHL00000000	43	6	15				20		1													1	
AB_2008-15-	AMHM00000000	19	2	17																				
AB_2008-15-	AMHN00000000	17	3																				14	
AB_2008-15-	AMHO00000000	43	6	15				20		1													1	
AB_2008-15-	AMHP00000000	21	2	19																				
AB_2008-23-	AMHQ00000000	29	3	16																			10	
AB_2008-23-	AMHR00000000	25	4	19												1	1							
AB_2009-04-	AMHS00000000	28	3	15				4															6	
AB_2009-04-	AMHT00000000	22	2	16											1		1						2	
AB_515-8	AMHU00000000	35	5	26											1		2						1	
AB_908-12	AMHV00000000	23	3	16											1		1						2	
AB_908-13	AMHW00000000	15		9					2	3							1							
AB_908-14-7	AMHX00000000	34	5	13				15			1													
AB_909-01-7	AMHY00000000	33	3	19																	1		10	
AB_909-02-7	AMHZ00000000	17		10					2	4							1							
AB_909-05	AMIA00000000	27		17	1								1	1	1		2		1				3	
AB_TG19617	AMID00000000																							
AB_TG2018	AMIE00000000	28	3	15																			10	
AB_TG2022	AMIF00000000	24	2	21													1							
AB_TG2023	AMIG00000000	24	2	21													1							
AB_TG2026	AMIH01	23	1	20												1	1							
AB_TG2028	AMII00000000	3	1	1											1									
AB_TG2030	AMIJ00000000	4	2	1											1									
AB_TG2031	AMIK00000000	4	1	2											1									
AB_TG2032	AMIL00000000	4	2	1											1									
AB_TG2631	AMIM00000000	22	2	17											1	1	1							
AB_TG27323	AMIN00000000	23	3	18												1	1							
AB_TG27327	AMIO00000000	31	5	23											1	1	1							
AB_TG27331	AMIP00000000	25	3	19											1	1	1							
AB_TG27335	AMIQ00000000	23	2	18											1	1	1							
AB_TG27339	AMIR00000000	9			2								2	3					2					
AB_TG27343	AMIS00000000	21	3	2			1		4	6					2					1			1	1
AB_TG5064	AMIU00000000	22	2	19													1							
AB0057	CP001182,CP00118	12	3	6						2							1							
AB030	CP009257.1	73	2	18														52		1				
AB031	CP009256.1	10		8					1								1							
Ab04-mff	CP012006.1	27		5						4	2						7			1			8	
AB07	CP006963.1,CP0069	10	4												1	1	4							
AB10I9	ATHY00000000	50	5	9	1			1					1	16		1	3		13					
Ab11111	AKAQ00000000	28	3	18											1		1						5	
AB18G7	ATHZ00000000	57	5	13	1								3	17		1	3		14					
AB1H8	ANNC00000000	15	2	12													1							
AB210	AEOX00000000	26	7	17											1	1								
AB307-0294	CP001172																							
Ab33333	AKAS00000000																							
AB405E4	ANND00000000	36			3	1				8					1		11			8				4
Ab44444	AKAT00000000	40	1	21						12		6												
AB4A3	AOLU00000000	16		15						1														
AB5075-UW	CP008706.1	5		2						2							1							
AB5711	AHAJ01	27	3	14										9	1									
AB900	ABXK00000000																							
AbH12O-A2	CP009534.1	13	1	6														3					3	
AB-HKU3-08	ATHY01	50	5	9	1			1					1	16		1	3		13					
AB-HKU3-10	THZ01	57	5	13	1								3	17		1	3		14					
ABIsac_ColiR	CAKB00000000			2																			4	
ABIsac_ColiS	CAKA00000000			3								1											2	
ABNIH1	AFSZ00000000	1		1																				
ABNIH11	APBA00000000	15	1	12																			2	
ABNIH13	APBB00000000	46	3	15					1						1		1					25		
ABNIH14	APBC00000000	45	4	15					1						1		1					23		
ABNIH15	APBD00000000	43	3	14					1						1		1					23		
ABNIH16	APBE00000000	54	4	19					1						1		1					28		
ABNIH17	APBF00000000	44	3	13					1						1		1					25		
ABNIH18	APBG00000000	30	2	17						6		5												
ABNIH19	APBH00000000	16	2	12																			2	
ABNIH2	AFTA00000000	2							1								1							
ABNIH20	APBI00000000	46	3	13					1						1		1					27		
ABNIH22	APBJ00000000	53	3	15					1						1		1					32		
ABNIH23	APBK00000000	49	4	14					1						1		1					28		
ABNIH24	APBL00000000	44	3	13					1						1		1					25		
ABNIH25	APBM00000000	2		1								1												
ABNIH26	AOGD00000000	3							1						1		1							
ABNIH3	AFTB00000000	2	1							1														
ABNIH4	AFTC00000000	39	2	20						10		7												
ABNIH5	APAW00000000	31	4	18						4		5												
ABNIH6	APAX00000000	15	1	12																			2	
ABNIH7	APAY00000000	17	4	11																			2	
ABUH30435(	JWSF00000000	29	4	19											1	1	1						3	

ABUH30435	JWSH00000000	29		5	18																	1	1		1									3
ABUH31911	JWRW00000000	29		3	21																	1	1		1									2
ABUH40457	JWSJ00000000	40		3	21								3	7			5																	1
ABUH42783	JWRU00000000	28		4	18																	1	1		1									3
ABUH46334	JWSE00000000	21		4	13																	1	1		1									1
ABUH4837	JWRT00000000	23		5	13																	1	3		1									
ABUH49714	JWRY00000000	23		2	15																	1	2		1									2
ABUH50422	JWRZ00000000	29		5	18																	1	1		1									3
ABUH50423	JWSA00000000	28		5	17																	1	1		1									3
ABUH51830	JWSC00000000	28		4	18																	1	1		1									3
ABUH52435	JWSI00000000	21		1	17																	1			1									1
ABUH60486	JWRV00000000	26		5	17																	1	2		1									
ABUH80286	JWSB00000000	27		4	18																	1	1		1									2
AC29	CP007535.2	8		2	1																	1	2		2									
ACICU	CP000863,CP00086	12		1		1																							2					8
ANC_4097	APRF00000000	14		3	1							2			4	1									1							1		1
ATCC_17978	CP000521,CP00052	6			1								2	2					1															
ATCC_17978	CP012004.1	4											2	1					1															
ATCC_19606	ACQB00000000												1																					
AYE	NC_010410.1			3	21																													
BIDMC_56	JMUH00000000	38		6	21																	1	1		1									8
BIDMC_57	JMUI00000000	28			9									4	4							9			1	1								
BJ1	JPLF00000000	19		4	13																	1			1									
BJ2	JPLG00000000	21		5	14																	1			1									
BJ3	JPLH00000000	15		3	11																		1											
BJ4	JPLI00000000	14					1			4				2	4							1			2									
BJ5	JPLJ00000000	18		4	11																	1	1		1									
BJ6	JPLK00000000	26		6	18																	1			1									
BJ7	JPLL00000000	26		6	18																	1			1									
BJ8	JPLM00000000	21		5	14																		1		1									
BJAB07104	CP003846.1,CP0039	22		4	15																	1	1		1									
BJAB0715	CP003847.1,CP0038	22			13									1						2									1					5
BJAB0868	CP003849.1,CP0038	18		5	12																		1											
BZICU-2	ALOH00000000	21		1		2	2							3	1				2	2				1			3			2	1	1				
Canada_BC1	AMSZ00000000	18		1	12										4										1									
Canada_BC-	AFDN00000000	18		1	12										4										1									
CI77	AVOC00000000	24		3	19																	1			1									
CI78	AVOE00000000																																	
CI79	AVOD00000000	30		2	5												8					1		12										2
CI86	AVOB00000000	29		2	5												8							12										2
CIP_70		2											2																					
CR17	CCCV000000000			4	13																	1			1									
CS01	CCCU000000000			5	13																	1			1									
D1279779	AERZ00000000	15													15																			
D36	CP012952.1	20		1	15									1		3																		
DU202	AVGF00000000	39		3	14							21				1																		
EGD-HP18	AVST00000000	4													1										3									
GTC_03324	BBNH01																																	
GTC_03329	BBNJ01																																	
IOMTU_433	AP014649.1,AP014650.1				6															1				1							1					2
IS-116	AMGF00000000	4			4																													
IS-123	ALII00000000	14		2	1																	1												10
IS-143	AMGE00000000	16		5	1													10																
IS-235	AMEI00000000	7			2										2										1									2
IS-251	AMEJ00000000	7			2										2										1									2
IS-58	AMGH00000000	7			2										2										1									2
JCM_6841	BALA00000000																																	
LAC-4	JICJ00000000	64			19										11		7							11	6									10
LY1	JDSV00000000	2													1										1									
LY2	JDSW00000000	1																							1									
LY3	JDSX00000000	27		5	18																	1	1		2									
LY4	JDSY00000000	24		4	18																	1			1									
LY5	JDSZ00000000	24		4	18																	1			1									
LY6	JDTA01	35		6	20										6							1	1		1									
LY7	JDTB01	36		6	20										6		1					1	1		1									
LY8	DTC01	34		5	20										6							1	1		1									
LY9	JDTD00000000	26		4	19																	1			2									
MAR002	JRHB00000000																																	
MDR_MMC4	AZNQ00000000	40		4	14									1	2			6														12		1
MDR-TJ	NC_017847.1	19		4	12																	1	1		1									
MDR-ZJ06	CP001937,CP00193	23		5	11											2	1					1	2		1									
MRSN_3405	JNOU00000000	2																1				1												
MRSN_57	JABT00000000	2	1	1																														
MRY09-0642	BASA00000000			3	6																	1	1		1									
MRY10-0558	BASB00000000			4	8																	1	1		1									
MRY12-0277	BASC00000000			4	6																	1	1		1									
MSP4-16	AODW00000000	2											2																					
Naval-113	AMZU00000000	25		4	19																	1			1									

Naval-13	AMDR00000000	16	2	3																														11	
Naval-17	AFDO00000000	24	3	17																		2			1									1	
Naval-18	AFDA00000000	12	2	7																				1										2	
Naval-2	AMSX00000000	36	3	15										9			9																		
Naval-21	AMSY00000000	26	4	21																					1										
Naval-57	AMFP00000000	3											1																	1					1
Naval-72	AMFI00000000																																		
Naval-78	AMFZ00000000	33	2	17							1			7			6																		
Naval-81	AFDB00000000	15	2	2																		1												10	
Naval-82	AMSW00000000	85		1		1							15	17		1		2	11						8	1		7	3	1				17	
Naval-83	AMFK00000000	32	2	14		1							8															1	1					5	
NCGM_237	AP013357.1		3	19																		1	1											26	
NIPH_1362	APOR00000000	7	1	3																														3	
NIPH_146	APOU00000000																																		
NIPH_1669	APOQ00000000	7	2																															5	
NIPH_1734	APOX00000000	8	1															1											1					5	
NIPH_190	APPL00000000	68											17	20			2		8						4	1		1		1		1		13	
NIPH_201	APQV00000000	1																							1										
NIPH_2061	APOW00000000	13		8																		1			2									2	
NIPH_24	APOF00000000	7	4	3																															
NIPH_290	APRD00000000	12	2	1									2																					7	
NIPH_329	APQY00000000																																		
NIPH_335	APQX00000000	21	1	7	1							1	1	4											6										
NIPH_410	ATGJ00000000	33											1	2								4			10					16					
NIPH_527	APQW00000000	4	3	1																															
NIPH_528	APRB00000000	4	3	1																															
NIPH_60	APPM00000000	11											1	1											5					4					
NIPH_601	APQZ00000000	9							1					4								1			3										
NIPH_615	APOV00000000	33			1							1		2				1	3			6			13			3		3					
NIPH_67	APRA00000000																																		
NIPH_70	APRC00000000	15			1									2				1	1			1			8			1							
NIPH_80	APRE00000000	59											4	23								2			18	1				3				8	
OIFC0162	AMFH00000000	10		2	1													1	1							4		1							
OIFC021	AMFR00000000	6																							1	4				1					
OIFC032	AFCZ00000000	6												2								3			1										
OIFC035	AMTB00000000	1											1																						
OIFC047	AMFW00000000	3																							2					1					
OIFC065	AMFV00000000	1		1																															
OIFC074	AMDE00000000	19	1	17																					1										
OIFC087	AMFS00000000	18												3								1			5									9	
OIFC098	AMDF00000000	12		3										3											6										
OIFC099	AMFT00000000	13		1										2								6			4										
OIFC109	ALAL00000000	14	2	2																														10	
OIFC110	AMFL00000000																																		
OIFC111	AMFY00000000	1											1																						
OIFC137	AFDK00000000	14	2	2																														10	
OIFC143	AFDL00000000	8	2	3												1								1			1								
OIFC180	AMDQ00000000	22	2	16																		1		1	1									1	
OIFC189	AFDM00000000	25	6	17																		1			1										
OIFC338	AMFX00000000	27	4	21														1																1	
ORAB01	JZCP00000000	30	6	21																		1	1		1										
ORAB02	JZCO00000000	29	4	22																		1	1		1										
ORAB03	JZCN00000000	28	3	22																		1	1		1										
ORAB08	JZCM00000000	29	4	22																		1	1		1										
ORAB09	JZCL00000000	30	4	23																		1	1		1										
ORAB10	JZCK00000000	58	1	25									10	11			5																	6	
ORAB13	JZCJ00000000	31	5	23																		1	1		1										
ORAB14	JZCI00000000	29	3	23																		1	1		1										
ORAB15	JZCH00000000	29	4	23																		1			1										
ORAB16	JZCG00000000	30	5	22																		1	1		1										
ORAB17	JZCF00000000	30	5	22																		1	1		1										
ORAB18	JZCE00000000	31	5	23																		1	1		1										
ORAB19	JZCD00000000	31	5	23																		1	1		1										
ORAB21	LRME00000000	31	4	25																		1			1										
ORAB23	LRMF00000000	28	4	22																		1			1										
ORAB6B	LRMJ00000000	34	4	28																		1			1										
ORABASHR	LRMG00000000	30	1	6																						23									
ORABMAYT	LRMI00000000	37	7	28																		1	1												
ORABRICT	LRMH00000000	31	1	5																						25									
ORABSC	LRMK00000000	30	4	23																		1	1		1										
Pulsotype_2	CBXD00000000																1					1													
R1B	JICK00000000	11	2	2									2	3											1									1	
SDF	CU468230,CU468231,CU468232,CU468233						179	180																											
T122	JRTX00000000	2	1																				1												
T173	JRTY00000000	13	5	3																		1	1		2							1			
T185	JRQU00000000	12	1			1								3								1			3					1		1	1		
T188	JRQV00000000	9		1									1	3								1								1		1	1		

T214	JRTZ00000000	1																								1									
T229	JRQW00000000	7				1								2								3			1									
T25	JPKX00000000	18	5	5																		1	1		2							4		
T258	JRQX00000000	7		1										2								1		1	1								1	
T271	JRQY00000000	10		1	1						2			2				1	1						1			1						
T7	JRQT00000000	11	5	2																		1	1		1							1		
T87	JRQS00000000	22	2	4		1							5	5								1	1		1							2		
TCDC-AB071	CP002522,CP00252	3	2																															1
TG00314	ASER00000000	4				4																												
TG02011	ASES00000000	23	1	9																						13								
TG02017	ASET00000000	42		4	1						1			3				5	4			4			12			3		5				
TG07725	ASEU00000000	14	1	12																		1												
TG15233	ASEV00000000	58	3	16									4									1			1								33	
TG15234	ASEW00000000	65	5	20									6	6		9	7							11										1
TG15236	ASEX00000000	54	3	18									2									2			1								28	
TG15237	ASEY00000000	48	4	15									1	1								1			1								25	
TG15238	ASEZ00000000	54	4	15		1							2	1								1			1								29	
TG15239	ASFA00000000	55	4	16									5									1											29	
TG15240	ASFB00000000	36	5	16										5			6																	4
TG15241	ASFC00000000	50	7	19									6	6			8																	4
TG15242	ASFD00000000	38	4	21									3	3			7																	
TG19582	AMIV00000000	2		1								1																						
TG2012	ASFE00000000	24	2	21																					1									
TG2013	ASFF00000000	30	5	21																		1	1		1									1
TG2014	ASFG00000000	25	5	17																		1	1		1									
TG20277	ASFH00000000	16	3	10										2											1									
TG20546	ASFI00000000	38	5	18										6			5																	4
TG22110	ASFJ00000000	24	1	22																					1									
TG22112	ASFK00000000	24	2	15									1	4									1		1									
TG22142	ASFL00000000	27	2	7									1			1					1			15										
TG22146	ASFM00000000	41	3	8									1		1	12								16										
TG22148	ASFN00000000	24	5	11										6									1		1									
TG22150	ASFO00000000	41	2	8									1		1	11					1			17										
TG22190	ASFP00000000	21	2	15										3											1									
TG22192	ASFQ00000000	25	1	23																					1									
TG22194	ASFR00000000	21	4	10										4									1		1	1								
TG22196	ASFS00000000	20	5	10										3									1		1									
TG22198	ASFT00000000	4											4																					
TG22202	ASFU00000000	30	2	23																		1	3		1									
TG22204	ASFV00000000																																	
TG22212	ASFW00000000	30	1	22									6												1									
TG22214	ASFX00000000	23	3	12									2	4									1		1									
TG22332	ASFY00000000	8	3	4																		1												
TG22336	ASFZ00000000	7	3	3																		1												
TG27295	ASGA00000000	9	1	6																		2												
TG27299	ASGB00000000	37	6	26																		1			2									2
TG27307	ASGC00000000	34	3	18										3			6					1	1		1									1
TG27311	ASGD00000000	31	4	18										1			2					1			2									3
TG27315	ASGE00000000	24	3	18																		1	1		1									
TG27319	ASGF00000000	25	3	19																		1	1		1									
TG27371	ASGG00000000	25	2	19																		1	1		1									1
TG27379	ASGH00000000	31	5	23																		1	1		1									
TG27383	ASGI00000000	28	5	20																		1	1		1									
TG27387	ASGJ00000000	3												1																		2		
TG27391	ASGK00000000	166		9	9	16						22	24	24				12	13			1			9	3		7	9	2		3		3
TG27395	ASGL00000000	34	2	19																														13
TG27399	ASGM00000000	30	1	19																														10
TG27407	ASGN00000000	32	5	23																		1	1		1							1		
TG27411	ASGO00000000	31	5	23																		1	1		1									
TYTH-1	CP003856.1	11	3	6																			1		1									
TYTH-2	AGSQ00000000	21	4	15																			1		1									
TYTH-3	AGSR00000000	20	4	14																			1		1									
TYTH-4	AGSS00000000	24	5	15																		2	1		1									
TYTH-5	AGST00000000	22	5	13																		2	1		1									
TYTH-6	AGSU00000000	17	1	13																		1	1		1									
TYTH-7	AGSV00000000	4									1														3									
UH0207	AYGS00000000	23	4	16																		1	1		1									
UH0707	AYGR00000000	77	3	28	3								20	15				2	2									2						2
UH0807	AYGQ00000000	76	3	29	3								19	14				2	2									2						2
UH10007	AYGO00000000	23	2	16																		1			1									3
UH1007	AYGP00000000	25	3	18																		1			1									2
UH10107	AYGN00000000	28	5	18																		1	1		1									2
UH106_429	JWVB00000000	84	1	7										1												75								
UH106_440	JWUZ00000000	86	1	7										1												77								
UH106_447	JWUW00000000	32	3	22																		1												6
UH10707	AYOI00000000	46	4	31									11																					
UH11608	AYGL00000000	30	5	18																		1	3		1									2
UH12208	AYGK00000000	46	4	5																						37								

UH12308	AYGJ00000000	26	3	20								1		1			1
UH12408	AYGI00000000	26	3	20								1		1			1
UH126_102	JWXL00000000	17	3	12								1		1			
UH126_106	JWXJ00000000	21	3	16								1		1			
UH126_137	JWXF00000000	24	5	13								1	3	1			1
UH12808	AYGH00000000	28	2	21								1		1			3
UH129_404	JWVJ00000000	29	5	18			1					1	1	1			2
UH129_408	JWVI00000000	27	5	17								1	1	1			2
UH129_409	JWVH00000000	29	6	18								1	1	1			2
UH129_415	JWVF00000000	30	6	19								1	1	1			2
UH134_167	JWXA00000000	26	4	16								1	1	1			3
UH134_168	JWWZ00000000	26	3	17								1	1	1			3
UH134_192	JWWW00000000	29	5	18								1	1	1			3
UH13908	AYGG00000000	25	4	18								1	1	1			
UH14508	AYGF00000000	31	5	20								1	1	1			3
UH15208	AYGE00000000	32	5	21								1	1	1			3
UH16008	AYGD00000000	32	5	21								1	1	1			3
UH16108	AYGC00000000	28	2	21								1		1			3
UH16208	AYGB00000000	47	4	5											38		
UH17_151	JWXB00000000	27	2	18								1		2			4
UH17_182	JWWY00000000	26	2	18								1		2			3
UH17_202	JWWT00000000	29	3	19								1		2			4
UH17_465	JWUR00000000	61	3	29	3		12	6		2	2					2	2
UH17_52	JWYK00000000	1		1													
UH175_677	JWTP00000000	123	4	28	5		46	27		4	4					4	1
UH175_679	JWTO00000000	56	1	20			11	11	6								7
UH184_295	JWWE00000000	19		16								1		1			1
UH184_309	JWWA00000000	27	7	14								2	2	1			1
UH184_310	JWVZ00000000	27	8	13								2	2	1			1
UH18608	AYGA00000000	27	5	17								1	3	1			
UH189_348	JWVU00000000	96	3	30	4		29	21		3	3					3	
UH189_349	JWVT00000000	84	3	28	1		31	17		1	1					1	1
UH189_355	JWVS00000000	99	3	28	4		32	22		3	3				1	3	
UH195_560	JWTX00000000	29	7	17								1	2	1			1
UH195_568	JWTV00000000	28	6	15								1	3	1	1		1
UH195_573	JWTT00000000	32	7	15	1					1	1	1	3	1		1	1
UH19608	AYFZ00000000	47	4	5											38		
UH19908	AYFY00000000	31	5	19								1	1	1			4
UH20108	AYFX00000000	28	5	19								1	2	1			
UH225_433	JWVA00000000	29	3	20			1					1	1	1	1		1
UH225_472	JWUO00000000	29	5	20								1	1	1			1
UH225_492	JWUF00000000	28	5	19								1	1	1			1
UH225_493	JWUE00000000	30	6	20								1	1	1			1
UH22908	AYFV00000000	40	5	5											30		
UH2307	AYFU00000000	25	3	17								1		1			3
UH236_84	JWXV00000000	24	6	16								1		1			
UH236_85	JWXU00000000	25	6	17								1		1			
UH236_87	JWXT00000000	22	5	15								1		1			
UH243_14	JWYS00000000	29	4	19								1	1	1			3
UH243_33	JWYP00000000	29	4	19								1	1	1			3
UH243_43	JWYL00000000	30	5	19								1	1	1			3
UH243_54	JWYJ00000000	27	5	18								1	1	1			1
UH243_55	JWYI00000000	30	4	21								1	1	1			2
UH243_56	JWYH00000000	28	4	18								1	1	1			3
UH243_57	JWYG00000000	30	4	20								1	1	1			3
UH243_66	JWYA00000000	27	4	18								1	1	1			2
UH243_68	JWXZ00000000	31	5	20								1	1	1			3
UH243_73	JWXY00000000	30	4	20								1	1	1			3
UH2707	AYFT00000000	77	3	29	3		20	14		2	2					2	2
UH280_81	JWXW00000000	29	4	19								1	1	1			3
UH280_92	JWXS00000000	32	6	20								1	1	1			3
UH280_93	JWXR00000000	31	4	21								1	1	1			3
UH280_99	JWXO03000000	29	4	20								1	1	1			2
UH2907	AYFS00000000	76	3	29	3		19	14		2	2					2	2
UH293_373	JWVP00000000	22	2	17								1		1			1
UH293_377	JWVO00000000	28	4	18								1	1	1			3
UH293_412	JWVG00000000	26	3	18								1	1	1			2
UH299_338	JWVX00000000	29	4	18								1	1	1	1		3
UH299_341	JWVW00000000	31	2	17	1	1	1	1		1	1	1	1	1		1	2
UH299_342	JWVV00000000	31	2	18	1		1			1	1	1	1	1		1	3
UH315_100	JWXN00000000	26	4	18								1		1			2
UH315_101	JWXM00000000	27	4	17								1	1	1			3
UH315_39	JWYN00000000	76	3	29	3		20	14		2	2					2	1
UH315_58	JWYF00000000	79	4	29	4		18	14		3	3					3	1
UH326_445	JWUX00000000	56	3	28	2		11	5		2	2					2	1
UH326_461	JWUS00000000	61	3	27	2		14	7		2	2					2	2
UH326_495	JWUD00000000	65	4	28	3		15	7		2	2					2	2
UH348_13	JWYT00000000	78	4	28	3		20	14		2	2					2	3

UH348_25	JWYR00000000	26	4	18								1	2	1				
UH348_27	JWYQ00000000	76	3	29	3		19	14		2	2					2		2
UH355_545	JWUB00000000	100	3	34	5		27	15		5	5					5		1
UH355_569	JWTU00000000	102	3	34	2		35	21		2	2					2		1
UH367_548	JWTZ00000000	46	10	24								1	1	1				9
UH367_558	JWTY00000000	25	1	18								1						5
UH367_565	JWTW00000000	45	9	24								1	1	1				9
UH370_269	JWWM00000000	65	3	32	3		12	7		2	2					2		2
UH370_270	JWYA00000000	27	4	18								1	1	1				2
UH370_301	JWWB00000000	60	3	30	1		13	8		1	1					1		2
UH3807	AYFR00000000	21	4	14								1	1	1				
UH381_467	JWUP00000000	72	3	26	3		20	12		2	2					2		2
UH381_482	JWUM00000000	36	7	17	1		3	1		1	1	1	1	1		1		1
UH384_444	JWUY00000000	24	1	18								1						4
UH384_450	JWUV00000000	27	1	19								1						6
UH392_536	JWUC00000000	10		3										4			1	2
UH392_659	JWTR00000000	19	3	7														9
UH392_684	JWTN00000000	64	3	21			14	14	5									7
UH41_486	JWUL00000000	61	3	27	3		13	7		2	2					2		2
UH41_487	JWUK00000000	60	4	27	3		12	6		2	2					2		2
UH41_488	JWUJ00000000	68	4	29	4		13	7		3	3					3		2
UH41_489	JWUI00000000	62	4	28	3		13	7		2	2					2		1
UH41_490	JWUH00000000	63	3	29	3		13	7		2	2					2		2
UH41_491	JWUG00000000	68	4	27	4		14	8		3	3					3		2
UH410_103	JWXK00000000	29	2	20								1		1				5
UH410_108	JWXI00000000	27	2	18								1		1				5
UH410_128	JWXH00000000	28	2	21								1		1				3
UH410_96	JWXP00000000	24	2	17								1		1				3
UH430_459	JWUT00000000	63	3	27	1		16	9		1	1				2	1		2
UH430_466	JWUT00000000	69	4	29	3		17	8		2	2					2		2
UH455_38	JWYO00000000	25	4	16								1	3	1				
UH455_6	JWUT00000000	26	5	16								1	3	1				
UH455_61	JWYE00000000	27	4	17								1	1	1				3
UH455_62	JWYD00000000	27	4	16								1	3	1				2
UH475_197	JWUT00000000	30	6	20								1	1	1				1
UH475_239	JWUT00000000	32	6	21								1	1	1				2
UH484_95	JWUT00000000	27	4	18								1	1	1				2
UH487_41	JWUT00000000	30	4	20								1	1	1				3
UH487_63	JWUT00000000	29	4	20								1	1	1				2
UH487_64	JWUT00000000	31	5	20								1	1	1				3
UH487_78	JWXX00000000	29	6	19								1	1	1				1
UH507_247	JWUT00000000	30	5	19								1	1	1				3
UH507_263	JWUT00000000	30	5	19								1	1	1				3
UH507_300	JWUT00000000	31	6	18			1					1	1	1				3
UH5107	AYFQ00000000	3	2									1						
UH514_287	JWUT00000000	5	1	1								1	1	1				
UH514_288	JWUT00000000	27	4	16								1	3	1				2
UH5207	JWUT00000000																	
UH5307	JWUT00000000	30	5	19								1	1	1				3
UH532_386	JWUT00000000	35	6	22			2	1				1		1				2
UH532_387	JWUT00000000	34	6	21			2	1				2		1				1
UH532_392	JWUT00000000	34	6	22			2					1		1				2
UH535_418	JWUT00000000	28	8	14								2	2	1				1
UH535_419	JWUT00000000	29	9	14								2	2	1				1
UH535_423	JWVC00000000	98	1	7				1							88			1
UH5707	AYFN00000000	32	4	22								1	1	1				3
UH588_656	JWTS00000000	49	4	21			1							1	19			3
UH588_663	JWTQ00000000	63	5	23			2	2						1	27			3
UH6107	AYFM00000000	28	4	18								1	1	1				3
UH6207	AYFL00000000	25	4	16								1	1	1				2
UH6507	AYFK00000000																	
UH66_241	JWWQ00000000	28	5	16								1	3	1				2
UH66_268	JWWN00000000	28	5	16								1	3	1				2
UH66_276	JWWK00000000	30	5	18								1	2	1				3
UH6907	AYFJ00000000	48	4	5											39			
UH7007	AYFI00000000	34	4	15				7	7									1
UH71_277	JWWJ00000000	28	5	17								1	1	1				3
UH71_278	JWWI00000000	27	6	16								1	1	1				2
UH71_279	JWWH00000000	26	4	17								1	1	1				2
UH71_296	JWWD00000000	29	4	18			1					1	1	1				3
UH7607	AYFH00000000	50	3	6											41			
UH7707	AYFG00000000	86	3	29	3	4	22	17		2	2					2		2
UH7807	AYFF00000000	29	4	21								1	1	1				1
UH7907	AYFE00000000	59	1	6				1							51			
UH81_364	JWVR00000000	27	1	21								1		1				3
UH81_366	JWVQ00000000	27	4	18								1	1	1				2
UH81_389	JWVL00000000	31	6	19								1	1	1				3
UH81_452	JWUU00000000	26	4	16								1	1	1				3

UH8107	AYFD00000000	33			5	22																		1	1		1									3
UH8407	AYFC00000000	25			6	17																		1			1									
UH8707	AYFB00000000	25			6	17																		1			1									
UH8807	AYFA00000000	27			4	18																		1	1		1									2
UH8907	AYEZ00000000	26			4	17																		1	1		1									2
UH9007	AYEY00000000	29			4	19																		1	1		1									3
UH9907	AYOH00000000	34			6	21																		2	1		1									3
UMB001	AEPK00000000	51			5	21											9		4	7						4										1
UMB002	AEPL00000000	3																																		3
UMB003	AEPM00000000	17			3																															14
VEB	CU459141.1,CU459139.1,CU459				3	21		1									1																			
W6976	AIEG01	19			4	14																			1											
W7282	AIEH00000000	23			7	15																			1											
WC-136	AMST00000000	23				3		1						6	1			3						1			6					1				1
WC-141	AMSS00000000	13				3																	4	1												5
WC-323	AMZS00000000	17						1							1								5				10									
WC-348	AMZT00000000	9				2							1	1		1				1			1							1		1				
WC-487	AMZR00000000	34				2						1		2	3		3			1				2			7	1		1		1		2		8
WC-692	AMGG00000000																																			
WC-A-694	AMTA00000000	15			2	2																														11
WC-A-92	AMFU00000000	101					1	3					2	3	19				1	32				3			10			27						
WM99c	AERY00000000	4			1																			1			1									1
XH386	CP010779.1	24			6	15																		1	1		1									
ZW85-1	CP006768.1,CP0067		1			1																														
ZWS1122	AMGR00000000	27			4	20																		1	1		1									
ZWS1219	AMGS00000000	29			5	21																		1	1		1									

*GenBank records are available at http://www.ncbi.nlm.nih.gov/nuccore/[Accession]

Supplemental Table 4. IS copies in K. pneumoniae genomes

Genome	Accession*	Total	IS1294	IS1F	IS26	IS4321R	IS5	IS5075	IS6100	IS903B	ISEc21	ISEcp1B	ISHe3	ISKpn1	ISKpn6	ISKpn7	ISKpn8	ISKpn14	ISKpn18	ISKpn19	ISKpn2	ISKpn20	ISKpn21	ISKpn24	ISKpn25	ISKpn26	ISKpn28	ISKpn31
325	JQGN01	6												5							1							
342	NC_011283.1	0																										
349	JQSE01	9			1				2					5							1							
1084	NC_018522.1	5												5														
1158	NZ_CP006722.1	13												11													2	
4006	JDWP01	11								2				6				1								2		
4640	JSZL01	10	1	3										5							1							
5422	JGYH01	13			2	1		2	2					4				1		1								
7699	JDWM01	16		2	2			1	2	5										1			1		1	1		
32192	NZ_CP010361.1	18								2				6					2							7	1	
34618	NZ_CP010392.1	28			2				2	3				7					2							11	1	
101488	JSZK01	14			2			1				1		5				1	1					2			1	
101712	JSZJ01	12		2	2	1		2	2			1						1									1	
12-3578	AQOC01	15			1	1	3	1		1		2								1			1	1		3		
700603	AOGO01	2			1				1																			
909957	AXUL01	14		3	3	1		2	1	2		1													1			
1162281	AFQL01	2		1																			1					
1191100241	AFXH01	28		3	4				2	3		1		6					3	1						3	2	
100SGR	CCEX01	12		2	2				1	1	1	1						1							1	2		
1010_KPNE	JWGK01	0																										
1012_KPNE	JWGI01	0																										
101BO	CCEY01	36		1	4				1	1				6	2	2		1	4						1	11	2	
102BO	CCFM01	37		1	2				3	2				6	2	2		1	4					1	1	11	1	
1035_KPNE	JWFI01	25		1	3	1		2		7				3				2			1		1	2		1	1	
103BO	CCFN01	36		2	4				1	3				6	2	1			4						1	10	2	
1041_KPNE	JWFB01	28		3		2	2	3		9				1				1					1	1		3	2	
104BO	CCFO01	37		3	4				2	2				6	2	2		1	2						1	10	2	
1098_KPNE	JWDA01	12		3									1	7							1							
10PV	CCFP01	26		4	6	1		4	2			1		8														
11227-1	JDWN01	22			2	4		1	2	4				3	2	2										1	1	
1183_KPNE	JVZX01	3		1						1																1		
11PV	CCFQ01	40		2	5					2		2		5	2	2			3						1	14	2	
120_1020	ARSO01	7							1						2	2		1							1			
1239_KPNE	JVXM01	46		2		2	9	2		6				2			1	2				2	2	2		11	3	
1263_KPNE	JVWL01	8								1				3										1		3		
1294_KPNE	JVVC01	0																										
12PV	CCFR01	39		3	4					2		2		5	2	2			3							14	2	
1331_KPNE	JVTI01	17		2	2					2			3	4				1						2			1	
1335_KPNE	JVTE01	14				2		3		1												3	1	1		3		
13PV	CCFT01	41		3	5					2		2		5	2	2			3							15	2	
140_1040	ARSP01	8							1	1					2	2		1							1			
14PV	CCFS01	32		1	2	1				7				10			2									7	2	
15PV	CCFV01	1												1														
160_1080	JMMY01	37			4					2				6	2	2			2						1	16	2	
16BO	CCFU01	12			2			1	2					5				1					1					
17PV	CCFW01	21		3	5			1	2			1		8											1			
187_KPNE	JVQU01	2								1							1											
18PV	CCFX01	13		2	2	1		2	2			1						1							1		1	
194_KPNE	JVQM01	1						1																				
19PV	CCGA01	19		3	5	1		2	2	2		1						1								2		
1kgm	JXBI01	1																1										
20PV	CCFZ01	31		10	1	2				3											1		1			11	2	
21PV	CCGB01	35		2	5	5	1	1		5													1			14	1	
221_KPNE	JVPK01	12		1	1		4			1													1			4		
223_14	JRTV01	10			1	1			2			1			2	2				1								
22PV	CCFY01	4												2				2										

234-12	NZ_CP011313.1	11	1										4									1			3	2	
23PV	CCGC01	36	2	4					2		2		5	2	2			3							12	2	
24PV	CCGH01	32	3	3					2				6	2	2			3							9	2	
25BO	CCGD01	10											10														
26BO	CCGE01	8											8														
27BO	CCJA01	8	2	1					1				2												1	1	
280_1220	ARSQ01	5												2	2									1			
28BO	CCGG01	16	2	2	1		1	2					5				1					2					
29BO	CCGF01	36		3				2	2				6	2	2		1	3						1	12	2	
303K	AVAN01	16	2	4	1		1	2	1													2		1	2		
30BO	CCGK01	41	3	7				3	2				6	2	2		1	2						1	9	3	
31AVR	CCGI01	41	3	5				2	3				6	2	2		1	3						1	11	2	
32AVR	CCGJ01	11	2	2									7														
34AVR	CCGL01	2											2														
361_1301	APWF01	6												2	2		1									1	
36AVR	CCGM01	12	3	2					2				3			1									1		
37AVR	CCGN01	16	4	5	1		3	2	1																		
39AVR	CCGP01	18		1				2	1		1		5					5							2	1	
40AVR	CCGO01	23	4	4	1		3	2			1		7									1					
41AVR	CCGQ01	13	2	4				1			1						1								4		
42AVR	CCGR01	40	3	4				2	3				6	2	2		1	3						1	11	2	
436_KPNE	JVHD01	4	1	2					1																		
43AVR	CCGS01	43	2	4				2	3				6	2	2		1	3						1	14	3	
440_1540	APWG01	9		1				1	1					2	2		1										1
44AVR	CCGY01	34	2	3				2	2				6		1			3						1	11	3	
4541-2	JDWO01	22	2	3	2		1	1	3				5			2	1								1	1	
45AVR	CCGT01	5											5														
45T1-2A	CDMU01	0																									
46AVR	CCGV01	21		2				2	1		1		6					6							2	1	
47AVR	CCGU01	13	3	4	1		3	2																			
48AVR	CCGW01	34		6				1	2				6	2	2		1	3						1	9	1	
49BG	CCGX01	4											4														
500_1420	NZ_CP011980.1	24							2				6					2							13	1	
500_KPNE	JVER01	4							1				3														
501_KPNE	JVEQ01	4							1				3														
50BG	CCHA01	34	3	2					2				6	2	2			3							12	2	
51BG	CCGZ01	14		3	1		3		2											1		1	2		1		
52BG	CCHC01	14		1					5				3							1		3			1		
53BG	CCHB01	8	2										4			1									1		
540_1460	APWI01	6						1						2	2											1	
54BG	CCHD01	39	1	2	3		2		3				6	2	2		1	3						2	10	2	
55BG	CCHG01	13	3	2					1				7														
56BG	CCHE01	35	3	3	2		1		3				5	2	2			3							9	2	
570_KPNE	JVBQ01	25		1	1		3		2				7			1					6	2			1	1	
57BG	CCHF01	41	2	3	3		2		3				6	2	2		1	3						2	9	3	
58BG	CCHI01	41	3	4				1	3				6	2	2		2	2						1	13	2	
60BG	CCHH01	15		1	1		2	2			1		4				2							1		1	
62BG	CCHJ01	10	1	1					1		1		3				3										
63BG	CCHK01	12		1	2		1		3		1						3					1					
646_1568	APWJ01	5												2	2									1			
648_KPNE	JUYR01	28	2	1	1	1	2		10							3	1					1	2		3	1	
65BO	CCHM01	18	3		1		3		1				5				1					2				2	
66BO	CCHL01	18		1	1		1		2				7				4					1		1			
67BO	CCHN01	4											4														
68BO	CCHO01	22	2	3	2		3	2					5				1					3		1			
69BO	CCHQ01	8		1	1								4												1	1	
70BO	CCHP01	24	2	3	1		3	2					5				2					2		1	2	1	
71RE	CCHS01	35	2	5				1	3				6	2	2		1	3						1	7	2	
72RE	CCHU01	39	4	5				1	4				6	2	2		1	3						1	9	1	

731_KPNE	JUVI01	3												1			1						1				
733_KPNE	JUVG01	40			1	2	10	2		3				4					1							12	5
73RE	CCHT01	38		4	4				1	2				6	2	2		1	3						1	10	2
741_KPNE	JUUX01	2			1																					1	
743_KPNE	JUUV01	2			1																					1	
74RE	CCHR01	38		3	2				2	2				6	2	2		1	3							13	2
75RE	CCHW01	40		4	5				1	2				6	2	2		1	3						1	11	2
76RE	CCHX01	6		2										2											1		1
77RE	CCHV01	7												7													
780_KPNE	JUTF01	1								1																	
78RE	CCHY01	2												2													
79RE	CCHZ01	8		1										5				2									
813_KPNE	JURV01	0																									
81RE	CCIA01	4		1	1									2													
82RE	CCIC01	22		4	4	3		3	2			1		4						1							
833_KPNE	JURI01	1								1																	
834_KPNE	JURH01	1								1																	
835_KPNE	JURG01	1								1																	
836_KPNE	JURF01	1								1																	
837_KPNE	JURE01	1								1																	
83RE	CCID01	8		1	2				1			1		2				1									
845_KPNE	JUQV01	1												1													
84RE	CCIB01	8			1									6						1							
85RE	CCIE01	8		1	1									5									1				
863_KPNE	JUQD01	9		1	2					2				3												1	
865_KPNE	JUQB01	20		1		1		2		12			1					2			1						
86SGR	CCIF01	38		2	4				2	2				6	2	1		1	3						1	12	2
87SGR	CCIH01	44		2	7				3	2				6	2	2		1	4						1	12	2
887_KPNE	JUPH01	7								4				3													
88SGR	CCIG01	38		2	4				2	2				6	2	2		1	3						1	11	2
89SGR	CCII01	41		3	5				2	2				6	2	2		1	3						1	12	2
90SGR	CCIJ01	42		3	5				3	2				5	2	2		1	3						1	12	3
912_KPNE	JUOG01	1																			1						
913_KPNE	JUOE01	1																			1						
91SGR	CCJB01	12		3						1								1					2			5	
92SGR	CCIK01	10		2	2	2		1		1													1			1	
930_KPNE	JUND01	1																			1						
93SGR	CCJC01	9												9													
94SGR	CCIL01	15		2	4					3				2												2	2
95SGR	CCIM01	9		3	2									4													
96SGR	CCIN01	4			1									1												2	
97SGR	CCJD01	34		1	3				4	2		4		6					3							9	2
98_KPNE	JULE01	4								4																	
98SGR	CCIO01	8		2	2					1														1	1		1
99SGR	CCIQ01	14		2	1	1		2	2	1		2						1							1		1
9PV	CCIP01	23		4	4	1		3	2			1		8													
ATCC_11296	CDJH01	11		7										1				3									
ATCC_13883	JSZI01	7		2										4							1						
ATCC_13884	CDOT01	1	1																								
ATCC_25955	AQQH01	24		3	1		6			2								2								9	1
ATCC_BAA-1705	AOGQ01	23		3	3				1	5					2	2		1	3						1		2
ATCC_BAA-2146	NZ_CP006659.1	13			2				2			2		4					2				1				
B1	JSWX01	0																									
B199	LJCB01	12		1	4	2						2											2				1
B5055	AQCG01	6												5							1						
B86	LJCD01	4				1								3													
BAMC_07-18	JRQE01	3			3																						
BIDMC_1	JCNK01	33		2	1			1	1	2				6	2	2		2	2	1						10	1
BIDMC_10	JNVG01	34	2	2	1	4			1	2				6	2	2			3							8	1

BIDMC_11	JCNF01	16	2	2	4				2				2	2										2	
BIDMC_12A	JCNE01	44	4	2	6	2		2	2	2		6	2	2			2					2		8	2
BIDMC_12B	JCND01	47	4	2	7	4		2	2	2		6	2	2			1					2		9	2
BIDMC_12C	AXLG01	46	5	2	7	2		2	2	2		6	2	2			2					2		8	2
BIDMC_13	JCNC01	35	2	2	3	2			1	2		6	2	2			2							10	1
BIDMC_14	JCNB01	32		2	2				1	3	1	5	2	2			2							11	1
BIDMC_16	AXLF01	53	7	2	8	7			1	3		6	2	2			3							11	1
BIDMC_18A	JCNA01	43		2	4				3	2		6	2	2			3							17	2
BIDMC_18B	JMVX01	39		2	4				1	3		6	2	2			3							14	2
BIDMC_18C	AXLE01	43		2	5				3	3		6	2	2			3							15	2
BIDMC_18D	JCMZ01	18		2	1							1				1							1	11	1
BIDMC_21	AYIB01	14		2								1				1								9	1
BIDMC_22	AYIA01	14		2						3		7										2			
BIDMC_23	AYHZ01	5			2					1		1										1			
BIDMC_24	AYHY01	15			1					5		9													
BIDMC_25	AYHX01	18		2								15							1						
BIDMC_2A	JCNJ01	33	3		2	5				2		6	2	2			2							9	
BIDMC_31	JAPW01	12								2		6				1			2					1	
BIDMC_32	JCMY01	50	8	2	3	6			2	3		6	2	2			1	1						13	1
BIDMC_33B	JNVK01	20		2	6				1	2			2	2		1						2		2	
BIDMC_34	JCMX01	42		2	4				3	2		6	2	2			2							17	2
BIDMC_35	JCMW01	27			3	8		1		2						1					1	3		8	
BIDMC_36	AYHW01	3										3													
BIDMC_4	JCNI01	30	3		2	3				2		6	2	2			3							7	
BIDMC_40	AYHS01	31		14	1					4		1				1					1			8	1
BIDMC_41	AYHR01	17		2	1							1				1							1	10	1
BIDMC_42a	JCMV01	35	10		1	2				1		6	2	2			2							9	
BIDMC_42b	JCMU01	36	11		2	2				1		6	2	2			2							8	
BIDMC_45	JCMM01	45		2	5				1	2		6	2	2			3							19	3
BIDMC_46a	JCMT01	21		2	3							1				1							1	12	1
BIDMC_46b	JCMS01	4										4													
BIDMC_47	JCMR01	12			1	2		2	2			4													1
BIDMC_48	JCMQ01	9		1	3	2						2												1	
BIDMC_5	JCNH01	36	2	2	4	5			1	2		6	2	2			2							7	1
BIDMC_51	JCMP01	13				1						9			1	1								1	
BIDMC_52	JCMO01	3										2				1									
BIDMC_53	JCMN01	22		2	4							1				1							1	12	1
BIDMC_54	JMVY01	46	15	2	2	3				2		6	2	2			2							10	
BIDMC_55	JMVZ01	7			1	3						2												1	
BIDMC_60	JMWA01	39		2	6				1	2		6	2	2		1	3						1	11	2
BIDMC_61	JMWB01	0																							
BIDMC_68	JMWC01	41		2	5				1	2		6	2	2		1	3						1	14	2
BIDMC_69	JMWD01	7			2	2				1									2						
BIDMC_7A	JAPO01	31			1	4		1		2		6	2	2			3							10	
BIDMC_7B	JCNG01	30			1	4				2		6	2	2			3							10	
BIDMC85	LFAY01	7								1		6													
BIDMC86	LFAZ01	8								1		7													
BIDMC88	LFBA01	5			1			2																	2
BIDMC89	LFBB01	33	1			5		1	2	3		7	2			2					3	2		3	2
BIDMC90	LFBC01	17			1	1				4					2	2				1	1	1		3	1
BIDMC91	LFBD01	10		1	1			1		2		1				1		1	1					1	
BIDMC95	LFBE01	9			1	2	1	1		2											1			1	
BIDMC96	LFBF01	15			2			2				8						1	1					1	
BJ1-GA	CBTU01	1																	1						
blaNDM-1	NZ_CP009114.1	14			1						1	6					1							4	1
BWH_15	JCNP01	20	1		4	1			2			7	2	2										1	
BWH_2	JCNQ01	12			4				2		1	3				2									
BWH_22	JCNO01	34		2	5				1	2		6	2	2		1	1							10	2
BWH_28	AYIS01	10										8									2				

BWH_30	AYIQ01	15		2	3							10											
BWH_36	JCNN01	48	18		2	4				2		6	2	2			2					10	
BWH_41	JCNM01	35		2	1	1		4	1	1		6				1	2					15	1
BWH_45	JMWE01	36		2	3	1		3	1	3		6	2	1			2					11	1
BWH_46	JMWF01	3								1													2
BWH_47	JMWG01	36		2	2				1	3		5	2	2			4					14	1
BWH_48	JMWH01	10		2	2					3					2							1	
BWH53	LEZU01	29			2					3	3	5	2	2			3					9	
BWH58	LEZV01	14		2	3				1		1		2	2		1						2	
BWH62	LEZW01	7										7											
CAV1193	CP013322.1	16								1		3							2			8	2
CAV1344	NZ_CP011624.1	18								2		3							2			9	2
CAV1392	NZ_CP011578.1	19		1	1						2	4	2	2			4					3	
CAV1596	NZ_CP011647.1	30	2			1				2		6					4					15	
CCBH13327	JSER01	15		2	2	3		1										3	4				
CDPE01	CDPE01	24		6	4			2	2	4	2	3										1	
CDPH3020	LAFV01	10			3					2		2			1				1			1	
CDPH3707	LAFX01	10			4					2		2			1				1				
CDPH3823	LAFW01	8			3					1		2			1				1				
CDPH5262	LAFU01	10			4					2		2			1				1				
CG43	NC_022566.1	3										1											2
CH1034	CXPD01	8		2								6											
CH4	JSXA01	0																					
CHS_02	JMWI01	42		2	6			3	2	4		6	2	2			4			2		8	1
CHS_03	JMWJ01	47		3	8			4	2	2		6	2	2			4			2		10	2
CHS_05	JMWK01	46		2	7			3	2	4		6	2	2			4			2		11	1
CHS_06	JMWL01	41		2	5			3	2	4		6	2	2			3			2		9	1
CHS_07	JMWM01	41		2				3	4	4		6	2	2			3			2		11	2
CHS_08	JJNH01	32	3	1	1				1	3		5	1				2			1		13	1
CHS_09	JMWN01	42		2	5			3	3	4		6	2	2			3			2		7	3
CHS_11	JMWO01	30			6			2	2	3		6	2	2			4			2			1
CHS_12	JMWP01	46		2	8			4	1	4		3	2	2			4			2		11	3
CHS_13	JMWQ01	42		2	6			3	2	4		6	2	2			3			2		8	2
CHS_14	JMWR01	37		2	4			2	2	4		6	2	2			3			2		7	1
CHS_16	JJNG01	40		3	3	1	1	4		7		4	2	2			3			2		7	1
CHS_17	JMWS01	31			4			1	2	2		5	2	2			3			2		7	1
CHS_18	JMWT01	34			5			2	2	3		6	2	2			4			2		5	1
CHS_19	JMWU01	32		2	4			3	2	4		6	2	2			3			2	1		1
CHS_20	JMWV01	23						2		3		3	2	2			2			2		6	1
CHS_21	JJNF01	18		3	4					5		2										2	2
CHS_22	JMWW01	42		3	6			1	1	4		6	2	2			4			2		9	2
CHS_23	JMWX01	43		4	6			3	2	2		6	2	2			4			2		7	3
CHS_24	JMWY01	17		1							1	4	2	2			5					2	
CHS_25	JMWZ01	36		2				2	2	4		6	2	2			3			2		8	3
CHS_26	JMXA01	39		2		1		2	3	4		6	2	2			4			2		9	2
CHS_27	JMXB01	41		2	5			2	1	4		6	2	2			4			2		8	3
CHS_28	JMXC01	35		2	6			3	1	3		6	2	2			6			2			2
CHS_29	JMXD01	45		2	7			3	2	5		6	2	2			3			2		9	2
CHS_30	JMXE01	39		3				2	3	4		4	2	2			4			2		10	3
CHS_31	JMXF01	36		3	1				1	4		6	2	2			2					13	2
CHS_32	JMXG01	37		2		1		3	2	4		6	2	2			3			2		8	2
CHS_33	JMXH01	43		2	5			3	2	3		6	2	2			4			2		11	1
CHS_34	JMXI01	36		3	8			2	2	2		6	2	2			4			2			3
CHS_35	JMXJ01	40		2	7			2	1	4		6	2	2			3			2		8	1
CHS_36	JMXK01	50		2	8			3	4	5		6	2	2			4			2		9	3
CHS_37	JMXL01	45		2	8	1		4	2	2		6	2	2			4			2		7	3
CHS_38	JMXM01	39		2	5			1	2	2		6	2	2			4			2		9	2
CHS_39	JMXN01	45		2	6			3	2	4		6	2	2			4			2		9	3
CHS_40	JMXO01	38			4			2		4		5	2	2			4			2		10	3

CHS_41	JMXP01	42		2	5		2	2	4		6	2	2	4			2		9	2
CHS_42	JJNE01	40		4	2	1	2	1	7		4	2	2	3			1		9	2
CHS_43	JMXQ01	13			1		1	2	1		3	2	2		1					
CHS_44	JMXR01	40		1		1	3	2	4		6	2	2	5			2		9	3
CHS_45	JMXS01	33		2	6		3	2	4		6	2	2	3			2			1
CHS_46	JMXT01	45		3	6		4	2	5		6	2	2	3			2		8	2
CHS_47	JJND01	42		3	3	2	3	1	6		4	2	2	4			1		10	1
CHS_48	JMXU01	14			1	1	1	2	1		3	2	2		1					
CHS_49	JMXV01	32		2	3			1	3		6	2	2	3					7	3
CHS_50	JMXW01	38			6		2	2	4		6	2	2	3			2		8	1
CHS_51	JMXX01	48		2	7		4	2	6		5	2	2	4			2		11	1
CHS_52	JMXY01	48		2	7	1	3	2	4		7	2	2	3			2		11	2
CHS_53	JMXZ01	46		2	6	1	4	2	5		6	2	2	2			2		9	3
CHS_54	JMYA01	47		2	7		3	4	4		6	2	2	3			2		9	3
CHS_55	JMYB01	59	14	2	5	2	4	2	2		6	2	2	1			2		12	3
CHS_56	JMYC01	46		2	6	1	3	2	6		6	2	2	4			2		8	2
CHS_57	JMYD01	48		2	7	1	3	2	6		6	2	2	4			2		8	3
CHS_58	JMYE01	42		2	6	1	3	2	1		6	2	2	4			2		8	3
CHS_59	JMYF01	41		2		1	3	2	4		6	2	2	6			2	1	8	2
CHS_60	JJNC01	32			3		2	1	4		5	2	2	2			2		8	1
CHS_61	JMYG01	36			4		2	2	4		6	2	2	3			2		8	1
CHS_62	JMYH01	37		2			2	2	4		6	2	2	4			2		9	2
CHS_63	JMYI01	37			5		2	2	4		6	2	2	3			2		8	1
CHS_64	JMYJ01	39		2	6		2	1	1		6	2	2	4			2		8	3
CHS_65	JMYK01	47		2	7		3	2	4		6	2	2	4			2		10	3
CHS_66	JMYL01	8			3		1	2	1						1					
CHS_67	JMYM01	47		2	7	1	7	1	4		6	2	2	3			2		9	1
CHS_70	JMYN01	44			7		3	2	4		6	2	2	4			2		11	1
CHS_71	JMYO01	43		2			2	3	4		6	2	2	4			2		13	3
CHS_72	JMYP01	36			4		2	2	4		6	2	2	4			2		7	1
CHS_73	JMYQ01	47		2	7		3	2	4		6	2	2	4			2		10	3
CHS_74	JMYR01	47		2	8		2	3	4		6	2	2	3			2		12	1
CHS_75	JMYS01	31		2	3		1	1	6		6	2	2	4			2			2
CHS_76	JMYT01	30		2			4	3	4		6	2	2	3			2			2
CHS_80	JMYU01	40		2			3	2	4		6	2	2	3			2		13	1
CHS100	LEVL01	46		2	6		3	4	2		6	2	2	5			2		9	3
CHS101	LEVM01	47		2	7		5	4	2		6	2	2	4			2		8	3
CHS102	LEVN01	48		2	7		4	4	2		6	2	2	4			2		10	3
CHS103	LGKA01	28		2			2	2	4		2	2	2	4			2		5	1
CHS104	LEVO01	44		2	3		2	2	4		6	2	2	4			3		11	3
CHS105	LEVP01	45		2	4		2	2	4		6	2	2	5			2		11	3
CHS106	LEVQ01	36		2			4	1	4		6	2	2	2			2		9	2
CHS107	LEVR01	12		2	2		3	2		1	1				1					
CHS108	LGJZ01	48		2	5		4	2	4		6	2	2	4			2		13	2
CHS109	LEVS01	45		2	7		4	2	4		6	2	2	3			2		9	2
CHS110	LEVT01	43		2	4		2	2	4		5	2	2	4			2		11	3
CHS111	LEVU01	47		2	5		3	5	4		6	2	2	4			2		10	2
CHS112	LEVV01	9			4		2	2											1	
CHS113	LEVW01	49		2	7		2	5	4		6	2	2	4			2		10	3
CHS114	LEVX01	55		2	9		3	4	4		6	2	2	3			2		15	3
CHS115	LEVY01	50		2	8		2	4	4		6	2	2	4			2		11	3
CHS116	LEVZ01	43		2	4		3	1	4		6	2	2	5			2		9	3
CHS117	LEWA01	42		2	4		5		4		5	2	2	4			2		11	1
CHS118	LEWB01	50		2	7	1	4	4	4		6	2	2	4			2		9	3
CHS119	LGJY01	52		2	8		4	4	4		6	2	2	4			2		12	2
CHS120	LGJX01	45		2	5		2	2	4		6	2	2	4			2		11	3
CHS121	LEWC01	18		3	2		4	2	1	1	4					1				
CHS122	LEWD01	45			7		2	4	4		6	2	2	3			2		12	1
CHS123	LEWE01	39		2	1	1	7	1	3		5	2	2	3			2		9	1

CHS124	LGJW01	46	2	3		5	4	4		6	2	2		4		2		11	1
CHS125	LEWF01	43	2	6		4	4	1		6	2	2		4		2		8	2
CHS126	LEWG01	40	2	3		3	4	5		6	2	2		4		2		4	3
CHS127	LEWH01	38	2	3		4		1		6	2	2		5		2		8	3
CHS128	LEWI01	50	2	8		2	4	4		6	2	2		4		2		11	3
CHS129	LEWJ01	51	2	7		5	4	2		6	2	2		4		2		12	3
CHS130	LGJV01	42	2	4		2	4	4		6	2	2		3		2		9	2
CHS131	LEWK01	48	2	6		4	4	4		6	2	2		3		2		11	2
CHS132	LEWL01	38	2	3		3	2	1		6	2	2		4		2		8	3
CHS133	LEWM01	48	2	5		5	4	4		6	2	2		4		2		11	1
CHS134	LEWN01	51	2	8		5	4	4		6	2	2		3		2		11	2
CHS135	LEWO01	36	2			5	2	4		6	2	2		4		2		6	1
CHS136	LGJU01	11		1	2		2			1	2	2			1				
CHS137	LEWP01	42	2	2		3	2	3		5	2	2		3		2		13	3
CHS138	LEWQ01	38	2	2		3	4	2		6	2	2		2		2		9	2
CHS139	LEWR01	44	2	8		3		4		6	2	2		3		2		10	2
CHS140	LEWS01	9	2	3														2	2
CHS141	LEWT01	47	3	6		1	3	4		6	2	2		4		2		11	3
CHS142	LEWU01	50	2	8		5	3	5		5	2	2		4		2		10	2
CHS144	LEWV01	44	2	3				3		6	2	2		2				22	2
CHS145	LEWW01	13		1				3		9									
CHS146	LEWX01	6		2		1	2		1										
CHS147	LEWY01	40	2	3		5	1	4		3	2	2		3		2		12	1
CHS148	LEWZ01	40	2	4		2	2			6	2	2		4		2		12	2
CHS149	LEXA01	43		6		4	4	4		6	2	2		3		2		9	1
CHS150	LEXB01	47	2	5		2	4	4		6	2	2		4		2		11	3
CHS151	LEXC01	34		3			2	2		6	2	2	1	2			1	12	1
CHS152	LEXD01	50	2	7		5	4	4		5	2	2		4		2		10	3
CHS154	LEXE01	49	2	6		5	4	4		4	2	2		4		2		11	3
CHS155	LEXF01	38	2	2		4	4	4		6	2	2		3		2		5	2
CHS156	LEXG01	25	2	2		3	1	1		3	2	2		4		2		1	2
CHS157	LEXH01	41	2	4	1	2		3		5	2	2		3		2		12	3
CHS158	LEXI01	48	2	7		2	4	4		5	2	2		4		2		11	3
CHS159	LEXJ01	3													3				
CHS160	LEXK01	38	3	1	1	3	1	2		6	2	2		3		2		9	3
CHS161	LEXL01	15		4		3	2		2				2		2				
CHS162	LEXM01	10	2	1				1		2	2	2							
CHS163	LEXN01	33	2	1		4		3		4	2	2		3		2		9	1
CHS164	LEXO01	47	2	6		1	4	4		6	2	2		4		2		11	3
CHS165	LEXP01	41	2	2	1	1		4		6	2	2		4		2		13	2
CHS166	LEXQ01	49	3	7		4	4	4		5	2	2		6		2		8	2
CHS167	LEXR01	47	2	6		3	4	4		6	2	2		3		2		11	2
CHS168	LEXS01	43	2	4		4	4	3		6	2	2		3		2		9	2
CHS169	LEXT01	47	2	6	1	2	2	4		6	2	2		4		2		12	2
CHS170	LEXU01	46	1	7		3	4	3		6	2	2		4		2		9	3
CHS171	LGJT01	42	2	3		4	2	1		6	2	2		4		2		11	3
CHS172	LEXV01	47	2	6		3	4	4		6	2	2		4		2		10	2
CHS173	LEXW01	44	3	3		4	4	3		6	2	2		3		2		11	1
CHS174	LEXX01	1								1									
CHS175	LGJS01	26	2	5	3	1									1	3		7	4
CHS176	LEXY01	38	2	3	1	2	2	4		3	2	2		2		2		11	2
CHS177	LEXZ01	49	2	6		2	4	4		6	2	2		4		2		12	3
CHS178	LGJR01	43	2	3		3	2	4		6	2	2		3		3		12	1
CHS179	LEYA01	39	2	3		2		4		5	2	2		3		2		12	2
CHS181	LEYB01	51	2	8		5	2	4		6	2	2		5		2		11	2
CHS182	LEYC01	51	2	8	1	1	3	4		6	2	2		4		2		13	3
CHS183	LEYD01	42	2			2	4	4		6	2	2		4		2		11	3
CHS184	LEYE01	50	2	5		4	4	5		6	2	2		4		2		11	3
CHS185	LEYF01	45	2	6		4	4	2		6	2	2		4		2		8	3

CHS186	LEYG01	36	2	1		4	1	3		2	2	2		4		2	10	3
CHS187	LGJQ01	47	2	5		5	4	2		6	2	2		4		2	10	3
CHS189	LEYH01	39		6		2	2	4		6	2	2		3		2	9	1
CHS191	LEYI01	35	2	4		1		4		3	2	2		3		2	11	1
CHS192	LEYJ01	48	2	6		3	2	2		6	2	2		7		2	11	3
CHS194	LEYK01	24	2				1	2		3				2			12	2
CHS195	LEYL01	52	2	6		5	4	4		7	2	2		4		3	10	3
CHS196	LGJP01	42		6		2	4	4		6	2	2		3		2	10	1
CHS197	LEYM01	40	2	2	1	2	1	4		5	2	2		5		2	10	2
CHS198	LGJO01	56	2	9		5	4	4		6	2	2		4		2	12	4
CHS200	LEYO01	40		5		3	2	4		6	2	2		3		2	9	2
CHS201	LEYP01	12					2	1		1	2	2	3				1	
CHS202	LEYQ01	40	2	3		3	2	4		6	2	2		3		2	9	2
CHS205	LEYR01	35		4		2	2	1		4	2	2		3		2	12	1
CHS206	LEYS01	17	1	2						5	2	2			2		3	
CHS207	LEYT01	46	2	7		3		4		6	2	2		4		2	11	3
CHS208	LEYU01	25	3	4			2		2	4				5			4	1
CHS209	LEYV01	51	2	7	1	5	4	4		6	2	2		4		2	11	1
CHS210	LEYW01	49	2	6		4	4	4		6	2	2		4		2	11	2
CHS211	LEYX01	21	3				2		2	4				5			4	1
CHS212	LGJN01	46	2	3		5	2	4		6	2	2		4		2	12	2
CHS213	LEYY01	46	2	5		4	2	4		6	2	2		4		2	11	2
CHS214	LEYZ01	53	2	11		3	2	4		5	2	2		4		2	13	3
CHS215	LEZA01	43	2	5	1	2		4		4	2	2		4		2	12	3
CHS216	LEZB01	40	3	4		2	1	4		2	2	2		4		2	12	2
CHS217	LEZC01	50	2	7		5	4	3		6	2	2		4		2	11	2
CHS218	LEZD01	41	2	3		1	1	3		5	2	2		5		2	12	3
CHS219	LEZE01	37	2	3		3	1	3		1	2	2		3		2	12	3
CHS220	LEZF01	48	2	4		3	4	4		6	2	2		5		2	11	3
CHS221	LEZG01	38		4		2	3	4		6	2	2		4		2	7	2
CHS223	LEZH01	34		6		2	2	3		2	2	2		4		2	8	1
CHS224	LEZI01	44	2	7		1	3	4		6	2	2		4		3	7	3
CHS225	LEZJ01	46	2	6		3	2	3		6	2	2		4		2	12	2
CHS226	LEZK01	35				2	3	4		6	2	2		3		2	9	2
CHS228	LEZL01	53	2	8		6	4	4		6	2	2		4		2	11	2
CHS229	LEZM01	22	3			1	1		1	4				5			6	1
CHS230	LEZN01	48	2	6		5	2	4		6	2	2		4		2	11	2
CHS231	LEZO01	44	2	6		4	4	1		6	2	2		4		2	8	3
CHS232	LEZP01	48	2	6		3	4	4		6	2	2		5		2	9	3
CHS234	LEZQ01	50	2	6		7	4	4		6	2	2		2		2	11	2
CHS235	LEZR01	44		7		2	4	4		6	2	2		3		2	11	1
CHS236	LEZS01	44	2	5		2	4	4		6	2	2		3		2	9	3
CHS237	LEZT01	46	2	6		2	4	4		6	2	2		3		2	10	3
CHS238	LGJM01	50	2	7		5	3	4		6	2	2		4		2	11	2
CHS239	LGJL01	44	2	3		3	4	3		6	2	2		4		2	10	3
CHS240	LGJK01	49	2	7		2	4	4		6	2	2		4		2	11	3
CHS81	LEUU01	48	2	6		3	4	4		6	2	2		4		2	10	3
CHS82	LEUV01	51	2	8		4	4	4		6	2	2		4		2	10	3
CHS83	LEUW01	38	2	4	1	3	1	4		4	2	2		2		2	9	2
CHS84	LEUX01	45	2	4		5	2	4		6	2	2		3		2	12	1
CHS85	LEUY01	40	2	4		5	2	2		4	2	2		4		2	8	3
CHS86	LGKC01	29	3	6		1	1	1	1	4	2	2		4			3	1
CHS87	LGKB01	51	2	8		3	4	4		6	2	2		4		2	11	3
CHS88	LEUZ01	54	2	10		4	4	4		6	2	2		4		2	11	3
CHS89	LEVA01	40	2	4		3	2	4		6	2	2		3		2	9	1
CHS90	LEVB01	49	3	7	1	2	3	4		6	2	2		4		2	11	2
CHS91	LEVC01	33	2	3			2	5		5				4			9	3
CHS92	LEVD01	48	2	7		1	4	4		6	2	2		4		2	12	2
CHS93	LEVE01	24	1	5		1	1		2	4				5			4	1

CHS94	LEVF01	48		2	8			2	4	4				6	2	2			4					2		9	3
CHS95	LEVG01	47		2	6			4	4	2				6	2	2			4					2		10	3
CHS96	LEVH01	47		2	7			2	3	4				6	2	2			4					2		10	3
CHS97	LEVI01	31		2	5			2	2					3	2	2			3					2		5	3
CHS98	LEVJ01	45		2	4			3	2	4				4	2	2			4					2		14	2
CHS99	LEVK01	35		2				3	3	2				4	2	2			4					2		9	2
CICC10011	LBCM01	0																									
DMC0526	ARPO01	12		3		2			1	1					2	2											1
DMC0799	ARRZ01	7		1											2	2				1						1	
DMC1097	NZ_CP011976.1	23	1							2				6					3							11	
DMC1316	ARSB01	3													1	2											
DSM_2026	JYBC01	3								1				2													
DSM_30104	AJJI01	3		1										1							1						
Ecl8	CANH01	7								1				5													1
EGD-HP19-C	AUTW02	3					1																	2			
FDAARGOS_84	JTAX01	11			2			4		1													2			2	
G5-2	AQQI01	23		3	1		6			1								2								9	1
GN8	JWPY01	3		1						1														1			
HE12	LBLV01	12			2					1		1			2	2		1								3	
HK787	NZ_CP006738.1	10												6													4
HKUOPLC	NZ_CP012300.1	0																									
HS11286	NC_016845.1	13		1								4		5					1					1		1	
HSL4	APFG01	0																									
hvKP1	AOIZ01	2								1																	1
IA565	JPIQ01	8				1		1		1				2			1							2			
IS33	CBWI01	26		3	5				2	1		1		5				3	3						1	1	1
JCM_1662	BAKZ01	3	1	1																	1						
JCM_20051	BBEV01	0																									
JH1	AFQK01	4				1				1																2	
JHCK1	ANGH02	11		3	2	2				2							1										1
JS86C16	LDZO01	12				1				9																	2
JT4	JAPZ01	13		2	2			2	1	3		1								1			1				
Kb140	AQRD01	38			4				1	2				6	2	2		1	2						1	15	2
Kb677	AQPG01	36	3	2	1				2	3				6	2	2			5							9	1
KCTC_2242	NC_017540.1	16												10													6
KK207_1	LJOO01	35		2	4				1	3				6	2	2		1	3						1	8	2
KKBO-1	AVFC01	38		2	4				2	4				5	2	2		1	2						1	11	2
KKBO-4	AVFD01	43		3	4				2	3				6	2	2		1	2						1	15	2
KP-11	APWE01	8		1											2	2	1	1									1
Kp13	NZ_CP003999.1	13				3	1					1		6									1			1	
KP1-I	AQCJ01	7							1						2	2		1							1		
KP2-R	AQCI01	7							1						2	2		1							1		
KP3-S	AOSJ01	10		3					1						2	2		1							1		
KP4-R	APMF01	6							1						2	2									1		
KP5-1	CP008700.1	0																									
Kp52	NZ_FO834906.1	5												5													
KP5-R	APMG01	7							1						2	2		1							1		
KP617	CP012753.1	10			1	3		1																		3	2
KP-7	AQQD01	8		1		1		1							2	2		1									
KPB-1	AYOV01	39		3	3				2	4				6	2	2			2							13	2
KPB-2	AYOW01	39		4	3				2	3				6	2	2			2							13	2
KPLM21	CCVM01	10								8														1	1		
KPM_nasey	CDNA01	23		2	2	1		2	2			3		5				1	1				2	1			1
KpMDU1	AMWO01	32		2	4		1		1	3				5	2	2			2						1	7	2
KpN01	CP012987.1	5								1				4													
KpN06	CP012992.1	6					1			1				4													
KPNIH1	NZ_CP008827.1	25			2				2	2				6					2							10	1
KPNIH10	NZ_CP007727.1	26			2				2	2				6					2							11	1
KPNIH11	AKAD01	43		2	7				3	2				5	2	2		1	2						1	14	2

KPNIH12	AKAE01	46	3	7				3	2				6	2	2		1	2						1	15	2
KPNIH14	AKAF01	44	3	7				3	2				6	2	2		1	2						1	13	2
KPNIH16	AKAG01	45	2	8				3	2				6	2	2		1	2						1	14	2
KPNIH17	AKAH01	46	3	8				3	2				6	2	2		1	2						1	14	2
KPNIH18	AKAI01	45	3	7				3	2				6	2	2		1	2						1	14	2
KPNIH19	AKAJ01	47	3	8		1		3	3				5	2	2		1	2						1	14	2
KPNIH2	AJZV01	44	2	6				3	2				6	2	2		1	2						1	15	2
KPNIH20	AKAK01	45	2	7				3	2				6	2	2		1	2						1	15	2
KPNIH21	AKAL01	46	2	8				3	2				6	2	2		1	2						1	15	2
KPNIH22	AKAM01	47	2	8				3	2				6	2	2		1	2						1	16	2
KPNIH23	AKAN01	47	3	6				3	3				6	2	2		1	2						1	16	2
KPNIH24	NZ_CP008797.1	29		4				2	2				6					3							10	2
KPNIH27	NZ_CP007731.1	7					1		2								1					1			1	1
KPNIH29	NZ_CP009863.1	10							1				6				2						1			
KPNIH30	NZ_CP009872.1	23							2				6					2							12	1
KPNIH31	NZ_CP009876.1	12		3							1		3	2	2				1							
KPNIH32	NZ_CP009775.1	24		2					3				6					3							10	
KPNIH33	NZ_CP009771.1	26		1					2				6	2	2			4							9	
KPNIH4	AJZW01	44	2	7				3	2				6	2	2		1	2						1	14	2
KPNIH5	AJZX01	44	2	7				3	2				6	2	2		1	2						1	14	2
KPNIH6	AJZY01	45	2	7				3	2				6	2	2		1	2						1	15	2
KPNIH7	AJZZ01	45	2	7				3	2				6	2	2		1	2						1	15	2
KPNIH8	AKAA01	43	2	8				3	2				5	2	2		1	2						1	13	2
KPNIH9	AKAB01	45	2	7				3	2				6	2	2		1	2						1	15	2
KPPR1	NZ_CP009208.1	7											7													
KpQ15	AWOM01	5		2				1	1				1													
KpQ24	AWON01	6		2				1	1		1		1													
KpQ3	AMSU01	4		2				1	1																	
KPR0928	NZ_CP008831.1	25							2				6					2							14	1
KpVA-1	CDFM01	46	3	8				3	2				6	2	2		1	2						1	13	3
KpVA-10	CDFT01	45	2	5				4	2				6	2	2		1	4						1	14	2
KpVA-11	CDFU01	45	3	5				3	3				6	2	2		1	4						1	13	2
KpVA-12	CDFV01	37		4				2	2				6	2	2		1	4						1	11	2
KpVA-13	CDFY01	43	3	4				3	2				6	2	2		1	4						1	13	2
KpVA-14	CDFW01	43	3	5				3	2				6		2		1	4						1	14	2
KpVA-15	CDFZ01	43	2	7				2	2				6	2	2		1	2						1	13	3
KpVA-16	CDFX01	39	2	6				2	2				6	2	2		1	2						1	11	2
KpVA-2	CDFP01	34	2	4					2				6	2	2			3							11	2
KpVA-3	CDFR01	41	4	5	1		2	2	3				6	1	2			2	1						10	2
KpVA-4	CDFQ01	32	2	3					3				6	2	2			2						2	8	2
KpVA-5	CDFO01	47	3	7				3	3				6	2	2		1	2						1	15	2
KpVA-6	CDGA01	49	3	7				3	3				6	2	2		1	2						1	17	2
KpVA-7	CDGB01	47	3	6				3	4				6	2	2		1	2						1	15	2
KpVA-8	CDGC01	44	2	5				3	2				6	2	2		1	4							15	2
KpVA-9	CDGD01	44	3	4				3	2				6	2	2		1	4						1	14	2
LAU-KP1	AYQE01	10	3	4				2			1															
LCT-KP182	ATRN01	7	2	2	1			2																		
LCT-KP214	AJHE02	7	2	2	1			2																		
LCT-KP289	ATRO01	6	1	2	1			2																		
LZ	AJVY01	33		2	1	1	1		12				5				5						4		1	1
MGH_17	AYJN01	10											10													
MGH_18	AYJM01	6		4	1																				1	
MGH_19	AYJL01	0																								
MGH_20	AYJK01	0																								
MGH_21	AYJJ01	5		1	1				1				1												1	
MGH_29	JCNY01	29	2	2				2	1				6	2	2			3							7	2
MGH_30	AYJC01	11		1					3				6												1	
MGH_31	JCNX01	38	2	6	3			3	2				6					2							9	5
MGH_32	AYJB01	3											3													

MGH_35	JCNW01	12			4			2	2		2	2												
MGH_36	AYIZ01	11		2	2							7												
MGH_39	JCNV01	16		2	1							4	2	2						2	1		1	1
MGH_40	AYIX01	0																						
MGH_43	JCNT01	19		2	5			2	2			8												
MGH_44	AYIV01	0																						
MGH_45	JCNS01	31		2	3				1	2		6	2	2		1	2						8	2
MGH_46	AYIU01	10		2	2					1										1			3	1
MGH_47	JCNR01	22		2	3							1											16	
MGH_48	AYIT01	10		2	1					1		1								3	1			1
MGH_51	JMYV01	37		2	6				1	2		6	2	2		1	2					1	10	2
MGH_52	JMYW01	5		1		1				1						1							1	
MGH_59	JMYX01	23			1	2	1	1	1	2	1	6				1	2						5	
MGH_60	JMYY01	7		3	1											1							1	1
MGH_63	JJNB01	26			6	2	1	1	1	8	2								1	1	1		2	
MGH_64	JMYZ01	7		1	1		3					2												
MGH_65	JMZA01	15				2		1	1	6	2								1	1			1	
MGH_66	JMZB01	11				1				1						4							5	
MGH_67	JMZC01	36		2	5				1	4		6	2	2		1	3					1	8	1
MGH_68	JMZD01	0																						
MGH_69	JMZE01	16		3					2	1	1	2				1	1			3	1			1
MGH_70	JMZF01	15		1	5			2		5										1				1
MGH_71	JMZG01	29			3	2		1	2	2		6	2	2			2						6	1
MGH_72	JMZH01	10			3	3						4												
MGH_73	JMZI01	24			2					3	1	5	2	2			1						8	
MGH_74	JMZJ01	8			3					1					1								3	
MGH_75	JJNK01	24			5	2		1	1	10	2								1	1			1	
MGH_76	JMZK01	1																			1			
MGH_78578	NC_009648.1	10						1				5								2		1	1	
MGH_79	JMZL01	38		2	2				3	2		6	2	2		1	2					1	13	2
MGH_80	JMZM01	7			1	1		1								1				2				1
MGH101	LFAK01	10			3			2	2	1					1								1	
MGH102	LFAL01	14		3	1							2											6	2
MGH111	LFAM01	19			2	2		2	1	7	2								1	1			1	
MGH112	LFAN01	3										3												
MGH113	LFAO01	16		1	1		2	3	2	3									2				1	1
MGH114	LFAP01	13			1	1		2		2										3			1	3
MGH115	LFAQ01	15			1	2			1	6	2								1	1			1	
MGH116	LFAR01	4			1					2		1												
MGH117	LFAS01	16			1	2		1		6	3								1	1			1	
MGH118	LFAT01	11		2	1			1	2		1	3						1						
MGH123	LFAU01	14				2		2		1	1	5			1								1	1
MGH124	LFAV01	5		2	2																			1
MGH125	LFAW01	34	3	2	1				1	1		4	2	2			2						15	1
MGH126	LFAX01	2							2															
MGH81	LEZX01	15			1	5		4	1		1								1	1			1	
MGH82	LEZY01	7			1							5										1		
MGH83	LGJJ01	16			2	1						7	2	2									2	
MGH84	LEZZ01	3										2							1					
MGH89	LFAA01	12			4			2	2		3	1												
MGH90	LFAB01	11			1	2		1				2									3			2
MGH91	LFAC01	41	5	3	2	4				3		6	2	2			4						9	1
MGH92	LFAD01	0																						
MGH93	LFAE01	9		1	1							2			2	1				2				
MGH94	LFAF01	6								1		5												
MGH95	LFAG01	37		1					1	2		6	2	2			4						16	3
MGH96	LFAH01	4		1						2									1					
MGH97	LFAI01	18		2	4			2	2		3	1								2				2
MGH98	LFAJ01	5										5												

MP14	ATAK01	14		2	1				1	2					2	2		1							1	1	1
MRSN_1319	JSVB01	15			3	1		1	2	4		2												1			1
MRSN_2404	LBIK01	14		3	5			2		2		1														1	
MRSN_3562	LBIE01	11		2	2					1		2						1								3	
MRSN_3852	LBID01	5			2					1				2													
MRSN_6902	LBLU01	36		1	5				1	2				6	2	2		1	1							13	2
MRSN6920	JPGS01	9		1					1		1				2	2		1							1		
MRSN8157	JPGT01	10		3					1		1				2	2		1									
MS6671	LN824133.1	0																									
NB60	AZAP01	24		3	6		1		1	1		1		1			2	1		3						2	2
NJST258_1	NZ_CP006923.1	20	3					1		2				4					2							7	1
NJST258_2	NZ_CP006918.1	21	2							2				5					2							10	
NTUH-K2044	NC_012731.1	5												5													
PittNDM01	NZ_CP006798.1	10			1	3		1																		3	2
PMK1	NZ_CP008929.1	6										1						2								1	2
PR04	AOPN02	11			2			2	2	1				3						1							
RYC492	APGM01000001.1	9												9													
SA1	CBTW01	5								1				2													2
SB2390	CCBO01	3								1																2	
SB3193	CCCQ02	0																									
SB3432	NC_021232.1	0																									
ST147	JXBF01	12			3	1		1	2			1		2						1			1				
ST15_NDM-1	CDQG01	21		3	2	1		1	2	2		1					1	3								2	3
ST258	LGAB01	17		2	2				1	1					2	2		2	2						1		2
ST258_FL	LAKK01	44	2	2	3	2		2	1	3				6	2	2			5							13	1
ST258-490	ALIS01	16		2	2				1	3					2	2			1						1		2
ST258-K26BO	CANR01	35		3	4				1	3		1		3	2	2		1	1						2	11	1
ST258-K28BO	CANS01	32		2	3				1	2				3	2	2		1	1						2	11	2
ST-437	LART01	33		2	3	2	1	1	2	3		4		5				1	1	2			1	1	1	2	1
ST485	JXCH01	6		1	1			1				1		1			1										
ST512-K30BO	CAJM02	22			2				1	2				4		2			3							7	1
SY-A	LIPM01	49	12		5	2		2	2	2				6	2	2			1							12	1
SY-B	LIPL01	52	12		5	3		2	2	2				6	2	2			1							14	1
SY-B2	LIPK01	50	11		5	3		2	2	2				5	2	2			1							14	1
T2-1-1	JAQL01	8								1				5													2
T2-1-2	JAQM01	8								1				5													2
T69	CBTV01	2												1													1
Top52_#1721	JNFE01	9			2					4				1												2	
UCI_17	JNVF01	43		3	8				2			1		4	2	2		1	2				3		1	14	
UCI_18	JCML01	6			1			2		1								1								1	
UCI_19	JCMK01	34			4					2				6	2	2		1	2						1	13	1
UCI_20	JCMJ01	17			1	4				2				7							2					1	
UCI_21	JCMI01	28			4					2				5	2	2			2							9	2
UCI_22	JCMH01	27		2	3				1	2				5					2							10	2
UCI_25	JCMG01	11			4				2			2		1						1				1			
UCI_26	JCMF01	8		1										7													
UCI_33	JCME01	39	3	2	2	3		2	2	2				6	2	2			2							9	2
UCI_34	JCMD01	20				3	1	2		1								1					2			7	3
UCI_37	JCMC01	45	14		3			2	2	2				6	2	2			2					2		8	
UCI_38	JCMB01	45	9		7	2		3	2	2				6					2					2		10	
UCI_41	JCMA01	33			5	2		1	2	2				6	2	2			1					2		8	
UCI_42	JCLZ01	7								1				5													1
UCI_43	JCLY01	49	14		8	2		2	2	2				6					1							12	
UCI_44	JCLX01	38			6	2		2	2	2				6					1					2		15	
UCI_55	JMZN01	40	6		4	1		3	3	2				6	2	2			1					2		8	
UCI_56	JMZO01	13			1	1				1				7												2	1
UCI_59	JMZP01	25			4					2				5	2	2			2							7	1
UCI_60	JJNJ01	0																									
UCI_61	JMZQ01	32			6				1	3				3	2	2		1	2						1	11	

UCI_62	JMZR01	11			1	2	1	2										1			3	1	
UCI_63	JMZS01	30		1	5			1		2	1	6	2	2		2					7	1	
UCI_64	JJNI01	20				2	2	2		6								2	1		4	1	
UCI_67	JMZT01	40	10	2	1	3				4		6	2	2		2					7	1	
UCI_68	JMZU01	12		2	2					2		5								1			
UCI69	LGJI01	39	5	2	3	3				2		6	2	2		2					11	1	
UCI70	LFBG01	4		2						2													
UCI75	LFBH01	36		2	4				3	2		6				2					16	1	
UCI76	LFBI01	13		2	2							7			2								
UCI81	LFBJ01	44	4	2	4	2		4	3	2		6	2	2		2					10	1	
UCI82	LFBK01	14		2	2							5					1				2	2	
UCI91	LFBL01	52	3	2	8	6		2	4	3		6	2	2		2					11	1	
UCI92	LFBM01	5		2								3											
UCI93	LFBN01	34	3		3	1		2	2	2		6	2	2		2					9		
UCI94	LFBO01	9		1								2						2	2		1	1	
UCI95	LFBP01	33	2	2	1	5				2		6	2	2		3					7	1	
UCI96	LFBQ01	2			1					1													
UCICRE_1	JCNL01	38	6	2	2	4				2		6	2	2		2					9	1	
UCICRE_10	JCOA01	0																					
UCICRE_13	AYIC01	29			4					2		5	2	2		2					11	1	
UCICRE_14	AYIN01	9			4			2	2										1				
UCICRE_2	AYIL01	14			1	3		2				7									1		
UCICRE_4	AYIJ01	9		3	2																3	1	
UCICRE_6	AYII01	9								1		6										2	
UCICRE_7	AYIH01	11				4	1											1			5		
UCICRE_8	JMSY01	6			1					2		2									1		
UHKPC_52	ARVN01	3							1	1					1								
UHKPC01	APVP01	8		1					1				2	2	1					1			
UHKPC02	ARSK01	12		2		2			1	1			2	2						1		1	
UHKPC04	APVV01	9	1						1	1			2	2						1			1
UHKPC05	JNBM01	35			4				1	2		6	2	2	1	2				1	12	2	
UHKPC06	ARSJ01	7											2	2	1					1		1	
UHKPC07	NZ_CP011985.1	23								2		6				3					11	1	
UHKPC09	APVO01	7							1				2	2	1					1			
UHKPC17	ARSG01	11		3		1			1	1			2	2								1	
UHKPC179	ARSM01	14		8				2	1		1									1		1	
UHKPC18	ARSH01	10		1		2			1				2	2						1		1	
UHKPC22	APVU01	11		1		2			1	1			2	2						1		1	
UHKPC23	AQQA01	9	1						1	1			2	2	1					1			
UHKPC24	APVS01	7											2	2	1					1		1	
UHKPC26	APVT01	7							1				2	2	1					1			
UHKPC27	APVR01	7		1					1				2	2	1								
UHKPC28	ARRU01	6											2	2	1					1			
UHKPC29	ARVQ01	8	1										2	2	1					1		1	
UHKPC31	ARSI01	7											2	2	1					1		1	
UHKPC32	ARPQ01	11		3		2			1				2	2								1	
UHKPC33	NZ_CP011989.1	21								2		6				2					10	1	
UHKPC40	AQOT01	5								1			2	2									
UHKPC45	JMSX01	42	6	2	5				1	3		6	2	2		3					11	1	
UHKPC47	ARRV01	8							1	1			2	2	1					1			
UHKPC48	ARPP01	8				1			1				2	2						1		1	
UHKPC57	ARPR01	11	1					2	1		1		2	2						1		1	
UHKPC59	ARSE01	9				2			1				2	2						1		1	
UHKPC61	ARSC01	8	1										2	2	1					1		1	
UHKPC67	ARSL01	4											2	2									
UHKPC69	ARRW01	11		3		2				1			2	2								1	
UHKPC77	ARRX01	10		1		1			1	1			2	2						1		1	
UHKPC81	APVQ01	7		1									2	2	1							1	
UHKPC96	ARRY01	10		1		1			1	1			2	2						1		1	

UKKV901664	ATEZ01	19		2	4			1	1	3					2	2			2								2
VA360	ANGI02	35			3				1	3				5	2	2		1	2						1	13	2
VAKPC252	APVW01	4													2	2											
VAKPC254	APVX01	4													2	2											
VAKPC269	APVY01	6							1						2	2									1		
VAKPC270	APWA01	6	1						1						2	2											
VAKPC276	APWB01	7	1						1						2	2		1									
VAKPC278	ARVP01	36			2				1	2				6	2	2			2						1	16	2
VAKPC280	APVZ01	7	1						1						2	2									1		
VAKPC297	APWC01	9		3		1			1						2	2											
VAKPC309	APWD01	7	1						1						2	2		1									
WGLW1	AMLL01	5		2						1																	2
WGLW2	AMLM01	9		3	3					1																1	1
WGLW3	AMLN01	4		2																							2
WGLW5	AMLO01	21		2	7	1			1	1				7												1	1
XDR	JRGE01	16		3	2	1		2	2	2		3								1							
XH209	NZ_CP009461.1	0																									
755.rep1_KPNE	JUUI01000000	2			1																					1	
KP_ST11_OXA48	JNHB01	5		2	1				1	1																	
ST101_93999682	LJEU00000000	22		5	5		1	1	1			1		3				1		1				1		2	
ST101_94515423	LJEM00000000	23		5	6			3	1			1		3				2						1		1	
ST101_94516583	LJEC00000000	25		4	6	1		3	1	1		1		3				1						2		2	
ST101_94516965	LJEE01000001	24		3	7	1		2	1	1		1		3				1						2		2	
ST101_95017178	LJDK01000001	22		2	4			2	2			2		2			1	2						2	1	2	
ST101_95017300	LJDN01000001	23		2	4			1	2	3		2		1			1	2						2	1	2	
ST101_95136398	LJFE01000001	32		4	8			3	2	1		2		3				2						2		4	1
ST101_95137395	LJEA01000001	30		3	7	1		3	2			3		3				2						2		3	1
ST101_95138435	LJEJ01000001	25		3	5		1	3	2	1		2		3				1							1	3	
ST101_95708332	LJEL01000001	22		4	7			1	1			1		3				1						1		3	
ST101_95708389	LJEI01000001	4						1						3													
ST101_95708916	LJEK01000001	22		3	6			1	1	1		1		3				1						1	1	3	
ST101_96018673	LJDT01000001	19		2	2	1		2	2	1		2						2						2		3	
ST101_U44822	LJEN01000001	23		3	8			2	1			1		3				1						2		2	
ST14_944535499	LJDW01000001	32		3	6	3		2	1			2						1					1	2		8	3
ST1478_9399971	LJEQ01000001	8			4	2			2																		
ST2016_9513626	LJDZ01000001	33		4	9			3	3	1		2		3				1						2		4	1
ST2017_9501175	LJEF01000001	33		3	7	5	3	1	2	1		1		3				4								2	1
ST2017_9501184	LJEH01000001	25		2	7	3	1	1	2			2		3				1								2	1
ST2017_9501423	LJEG01000001	29		2	7	4	3	2	2			1		3				2								2	1
ST323_94153037	LJDR0000000	8		1	2			1	2	1		1															
UHKPC52	ARVN00000000	27			2				1	2				6				1	2							11	2
Yangling_I2	CP013338.1	5		1			2																2				
yzusk-4	CP011421.1	2																								2	

*GenBank records are available at http://www.ncbi.nlm.nih.gov/nuccore/[Accession]

Supplemental Table 5. *A. baumannii* genes with multiple different IS elements inserted

In Gene	#IS elemen	# Genomes	Description
M3Q_1781	11	30	competence/damage-inducible protein CinA
M3Q_926	10	75	ATPase
M3Q_1891	9	297	integrase
M3Q_2805	9	48	protein MorA
M3Q_2954	9	59	subtilisin-like serine protease
M3Q_3077	9	28	OmpA protein
M3Q_3141	9	50	surface adhesion protein
M3Q_1390	8	295	tape measure domain-containing protein
M3Q_1901	8	14	P pilus assembly protein, porin PapC
M3Q_1975	8	30	hypothetical protein
M3Q_2182	8	18	Signal transduction histidine kinase AdeS
M3Q_2832	8	8	hypothetical protein
M3Q_3266	8	73	hypothetical protein
M3Q_41	8	15	mutator family transposase
M3Q_798	8	23	diguanylate cyclase
M3Q_947	8	31	Signal transduction histidine kinase regulating C4-dicarboxylate transport system
M3Q_1692	7	6	shikimate transporter
M3Q_2158	7	32	hypothetical protein
M3Q_2379	7	9	ferrichrome-iron receptor
M3Q_2529	7	13	hypothetical protein
M3Q_2685	7	36	type 1 pili subunit CsuA/B protein
M3Q_2688	7	63	TetR family transcriptional regulator
M3Q_2895	7	13	hypothetical protein
M3Q_3078	7	53	hypothetical protein
M3Q_3420	7	338	hypothetical protein
M3Q_531	7	59	thioesterase
M3Q_589	7	4	Type II secretory pathway, ATPase PulE/Tfp pilus assembly pathway, ATPase PilB
M3Q_946	7	48	hypothetical protein
M3Q_1185	6	3	hypothetical protein
M3Q_1365	6	40	hypothetical protein
M3Q_1869	6	49	hypothetical protein
M3Q_1895	6	9	inner membrane protein, putative permease for cytosine/purines, uracil, thiamine, allantoin
M3Q_1976	6	18	flavoprotein
M3Q_1987	6	3	dipeptide ABC transporter substrate-binding protein
M3Q_2005	6	9	adenylate/guanylate cyclase
M3Q_2071	6	17	ribonuclease D
M3Q_2156	6	42	phosphatidylserine/phosphatidylglycerophosphate/cardiolipin synthase
M3Q_2242	6	13	RTX toxin
M3Q_2358	6	2	metal-dependent hydrolase
M3Q_2448	6	186	transcriptional regulator
M3Q_2527	6	15	hypothetical protein
M3Q_2560	6	5	hypothetical protein
M3Q_2694	6	4	AraC-type DNA-binding domain-containing protein
M3Q_2787	6	9	branched-chain amino acid permease (azaleucine resistance)
M3Q_2993	6	80	D-alanyl-D-alanine carboxypeptidase
M3Q_3167	6	11	poly-gamma-glutamate biosynthesis protein
M3Q_3476	6	7	TRAP-type C4-dicarboxylate transport system, large permease component
M3Q_352	6	17	hypothetical protein
M3Q_463	6	219	IS4 family transposase ORF 1
M3Q_730	6	18	Outer membrane receptor for monomeric catechols

M3Q_927	6	12	hypothetical protein
M3Q_935	6	25	hypothetical protein
M3Q_940	6	8	transposase
M3Q_101	5	279	PGAP1-like protein
M3Q_111	5	17	hypothetical protein
M3Q_1329	5	13	hemagglutinin-like protein
M3Q_1399	5	11	Lipid A phosphoethanolamine transferase, associated with polymyxin resistance
M3Q_1495	5	278	Rhs family protein
M3Q_1591	5	24	pyrroline-5-carboxylate reductase
M3Q_1620	5	23	drug/metabolite transporter permease
M3Q_1671	5	25	hypothetical protein
M3Q_1682	5	4	transcriptional regulator
M3Q_1711	5	20	SecC motif-containing protein
M3Q_1774	5	3	hypothetical protein
M3Q_1896	5	6	hypothetical protein
M3Q_1933	5	10	hypothetical protein
M3Q_2018	5	8	terminal alkane-1-monooxygenase
M3Q_203	5	9	transcriptional regulator
M3Q_2048	5	54	TonB-dependent siderophore receptor
M3Q_2082	5	56	diguanylate cyclase
M3Q_2112	5	99	TonB-dependent siderophore receptor
M3Q_2118	5	33	acetyl-CoA acetyltransferase
M3Q_214	5	9	low temperature requirement A protein (LtrA) family protein
M3Q_2160	5	6	hypothetical protein
M3Q_2241	5	37	multidrug resistance efflux pump
M3Q_2291	5	36	transcriptional regulator
M3Q_2396	5	2	hypothetical protein
M3Q_2419	5	8	sulfate permease
M3Q_2488	5	6	iron-regulated membrane protein
M3Q_2612	5	50	hemin-binding outer membrane transmembrane protein
M3Q_2680	5	5	major facilitator superfamily permease
M3Q_2695	5	7	threonine efflux protein
M3Q_2697	5	13	hypothetical protein
M3Q_2820	5	34	aminopeptidase N
M3Q_2885	5	120	regulator of polyketide synthase expression
M3Q_2998	5	6	hypothetical protein
M3Q_3047	5	9	porin protein associated with imipenem resistance
M3Q_3235	5	11	DcaP-like protein
M3Q_3251	5	2	DNA-binding domain-containing protein
M3Q_3271	5	4	long-chain fatty acid ABC transporter permease/ATPase
M3Q_3288	5	34	chemotaxis protein histidine kinase
M3Q_334	5	5	hypothetical protein
M3Q_3343	5	38	SAM-dependent methyltransferase
M3Q_3471	5	7	phosphoserine phosphatase
M3Q_435	5	12	outer membrane porin, OprD family
M3Q_627	5	17	hypothetical protein
M3Q_9	5	266	NAD/FAD-binding protein
M3Q_954	5	17	diguanylate cyclase
M3Q_1042	4	5	multimeric flavodoxin WrbA
M3Q_1073	4	4	Tyrosine--tRNA ligase, putative
M3Q_1164	4	1	hypothetical protein
M3Q_1187	4	4	type III restriction enzyme, res subunit family
M3Q_1200	4	27	ABC oligo/dipeptide transport, ATP-binding protein
M3Q_1202	4	15	hypothetical protein

M3Q_1279	4	15	TonB-dependent siderophore receptor
M3Q_1285	4	2	ABC transporter
M3Q_1325	4	1	heat shock protein
M3Q_1330	4	4	outer membrane protein/peptidoglycan-associated (lipo)protein
M3Q_1405	4	1	hypothetical protein
M3Q_1490	4	67	hypothetical protein
M3Q_1494	4	148	Rhs element Vgr protein
M3Q_1520	4	9	metal-dependent hydrolase
M3Q_1523	4	6	hypothetical protein
M3Q_1540	4	281	cAMP-binding protein
M3Q_1578	4	238	hypothetical protein
M3Q_1593	4	16	hypothetical protein
M3Q_1594	4	9	hypothetical protein
M3Q_1610	4	13	HlyD family type I secretion membrane fusion protein
M3Q_1632	4	19	beta-lactamase class A
M3Q_1633	4	3	Rhs element Vgr protein
M3Q_1686	4	3	hypothetical protein
M3Q_1691	4	2	hypothetical protein
M3Q_1712	4	25	retron-type reverse transcriptase
M3Q_1773	4	3	transcriptional regulator
M3Q_1777	4	3	hypothetical protein
M3Q_1785	4	8	hypothetical protein
M3Q_1890	4	6	hypothetical protein
M3Q_1897	4	2	DMT family permease
M3Q_1900	4	1	P pilus assembly protein, pilin FimA
M3Q_1921	4	115	hypothetical protein
M3Q_2051	4	2	membrane-fusion protein
M3Q_2054	4	5	multidrug ABC transporter permease
M3Q_2061	4	3	hypothetical protein
M3Q_2068	4	6	hypothetical protein
M3Q_2193	4	11	hypothetical protein
M3Q_2206	4	7	response regulator containing a CheY-like receiver domain and an HTH DNA-binding domain
M3Q_2288	4	24	symporter
M3Q_2357	4	1	hypothetical protein
M3Q_2395	4	1	hypothetical protein
M3Q_2418	4	11	hypothetical protein
M3Q_2421	4	7	esterase/lipase
M3Q_2422	4	6	gamma-aminobutyrate permease
M3Q_2487	4	19	transcriptional regulator
M3Q_2520	4	23	hypothetical protein
M3Q_2523	4	6	TonB-dependent siderophore receptor
M3Q_2526	4	5	acetyltransferase
M3Q_2576	4	5	hypothetical protein
M3Q_2670	4	9	hypothetical protein
M3Q_2683	4	21	P pilus assembly protein, porin PapC
M3Q_2686	4	26	protein CsuA
M3Q_2687	4	21	protein CsuA/B, secreted protein related to type I pili
M3Q_2702	4	6	acyl-CoA dehydrogenase
M3Q_2774	4	1	hypothetical protein
M3Q_2830	4	246	IS4 family transposase ORF 1
M3Q_2879	4	25	choline-glycine betaine transporter
M3Q_2880	4	3	transcriptional regulator
M3Q_2881	4	6	gamma-aminobutyrate permease
M3Q_2997	4	6	hypothetical protein

M3Q_3000	4	5	hypothetical protein
M3Q_3125	4	106	hypothetical protein
M3Q_3128	4	1	transcription elongation factor and transcript cleavage
M3Q_3151	4	5	phosphodiesterase/alkaline phosphatase D
M3Q_32	4	40	choline-glycine betaine transporter
M3Q_3234	4	3	Lipid A phosphoethanolamine transferase
M3Q_3394	4	31	Signal transduction histidine kinase
M3Q_3419	4	9	hypothetical protein
M3Q_3421	4	13	outer membrane protein
M3Q_3470	4	8	hypothetical protein
M3Q_3608	4	5	O-antigen ligase
M3Q_3609	4	8	prepilin-type N-terminal cleavage/methylation domain-containing protein
M3Q_3687	4	7	hypothetical protein
M3Q_385	4	3	glutathione peroxidase
M3Q_387	4	1	MFS family transporter
M3Q_532	4	9	DNA binding protein
M3Q_660	4	10	transcriptional regulator
M3Q_690	4	3	zinc-binding alcohol dehydrogenase family protein
M3Q_790	4	25	membrane-fusion protein
M3Q_809	4	5	transcriptional regulator
M3Q_818	4	1	Dehydrogenase/reductase
M3Q_925	4	7	transcriptional regulator
M3Q_100	3	127	esterase
M3Q_1007	3	3	trehalose-6-phosphate synthase
M3Q_1026	3	2	phosphatase
M3Q_1056	3	2	hypothetical protein
M3Q_1083	3	17	phosphomannomutase
M3Q_112	3	9	hypothetical protein
M3Q_1142	3	9	hypothetical protein
M3Q_1169	3	2	hypothetical protein
M3Q_119	3	2	hypothetical protein
M3Q_1229	3	2	BCCT family high-affinity choline transporter
M3Q_1234	3	6	Na+/H+ dicarboxylate symporter
M3Q_1271	3	3	phosphate transporter
M3Q_1281	3	7	lipase
M3Q_1334	3	4	signal peptide-containing protein
M3Q_1361	3	4	Lipoprotein, putative
M3Q_140	3	2	hypothetical protein
M3Q_1475	3	3	hypothetical protein
M3Q_1486	3	27	diguanylate cyclase
M3Q_1488	3	5	NADH:flavin oxidoreductase
M3Q_150	3	5	acyltransferase
M3Q_1503	3	28	gamma-aminobutyrate permease
M3Q_1504	3	3	glycine/D-amino acid oxidase, deaminating
M3Q_1507	3	3	transcriptional regulator
M3Q_1518	3	11	transcriptional regulator
M3Q_1519	3	9	pirin-like protein
M3Q_1527	3	15	signal peptide protein
M3Q_1539	3	148	IS4 family transposase ORF 1
M3Q_1543	3	6	metalloendopeptidase-like membrane protein
M3Q_1588	3	6	threonine efflux protein
M3Q_1608	3	6	RND efflux transporter
M3Q_1609	3	7	type I secretion system ATPase, LssB family
M3Q_161	3	5	transporter, sodium/bile acid transporter family protein
M3Q_1619	3	20	transcriptional regulator

M3Q_1630	3	2	acetyltransferase
M3Q_1648	3	4	transcriptional regulator
M3Q_1654	3	4	NADPH-quinone reductase
M3Q_1669	3	3	hypothetical protein
M3Q_1672	3	19	hypothetical protein
M3Q_1673	3	2	hypothetical protein
M3Q_1688	3	2	hypothetical protein
M3Q_1716	3	9	catalase
M3Q_1772	3	15	lysophospholipase L1
M3Q_1830	3	22	hypothetical protein
M3Q_1834	3	256	signal peptide protein
M3Q_1859	3	29	amino acid efflux protein, threonine efflux protein
M3Q_1872	3	4	D-and L-methionine ABC transporter ATP-binding protein
M3Q_1889	3	13	transcriptional regulator
M3Q_1912	3	362	Beta-lactamase class D
M3Q_1998	3	2	nucleoside-diphosphate-sugar epimerase
M3Q_2017	3	7	AraC-type DNA-binding domain-containing protein
M3Q_2057	3	6	hypothetical protein
M3Q_2066	3	3	Poly(3-hydroxyalkanoate) synthetase
M3Q_2078	3	36	hypothetical protein
M3Q_2085	3	4	lipoyl synthase
M3Q_2107	3	1	nitrate/sulfonate/bicarbonate ABC transporter permease
M3Q_2129	3	4	major facilitator superfamily permease
M3Q_2145	3	3	Fe2+/Zn2+ uptake regulation protein
M3Q_2157	3	3	hypothetical protein
M3Q_2162	3	3	hypothetical protein
M3Q_2169	3	5	hypothetical protein
M3Q_2181	3	5	response regulator AdeR
M3Q_2204	3	6	RND family drug transporter
M3Q_2236	3	2	fumarylacetoacetate (FAA) hydrolase family protein
M3Q_2260	3	16	hemagglutinin protein
M3Q_2264	3	3	hypothetical protein
M3Q_2277	3	4	hypothetical protein
M3Q_2311	3	3	Penicillin G amidase
M3Q_2359	3	4	alpha/beta hydrolase
M3Q_2371	3	3	hypothetical protein
M3Q_2388	3	2	histone acetyltransferase HPA2
M3Q_2406	3	7	alpha/beta hydrolase
M3Q_2412	3	2	hypothetical protein
M3Q_2416	3	8	stress protein
M3Q_2417	3	3	glucose/sorbosone dehydrogenase
M3Q_2420	3	2	subtilisin-like serine protease
M3Q_2428	3	4	Fels-1 Prophage Protein
M3Q_2433	3	4	hypothetical protein
M3Q_2455	3	4	hypothetical protein
M3Q_2513	3	15	magnesium-translocating P-type ATPase
M3Q_2516	3	3	signal peptide protein
M3Q_2519	3	134	ABC transporter ATPase
M3Q_2528	3	20	hypothetical protein
M3Q_2534	3	5	hypothetical protein
M3Q_2558	3	2	hypothetical protein
M3Q_2595	3	2	acetyl-CoA synthetase/AMP-(fatty) acid ligase
M3Q_2599	3	3	nitrate/sulfonate/bicarbonate ABC transporter substrate-binding protein
M3Q_2603	3	6	chaperone
M3Q_2611	3	4	xylanase/chitin deacetylase

M3Q_2615	3	18	hypothetical protein
M3Q_2634	3	4	hypothetical protein
M3Q_2667	3	164	histone acetyltransferase HPA2
M3Q_2678	3	15	DMT family permease
M3Q_2684	3	7	P pilus assembly protein, chaperone PapD
M3Q_2690	3	3	transcriptional regulator
M3Q_2803	3	5	methylenetetrahydrofolate reductase
M3Q_2817	3	9	amino acid/peptide transporter (peptide:H symporter)
M3Q_2831	3	3	Class C Beta-lactamase AmpC
M3Q_2836	3	4	hypothetical protein
M3Q_2890	3	5	hypothetical protein
M3Q_2978	3	4	hypothetical protein
M3Q_3011	3	4	hypothetical protein
M3Q_3012	3	7	hypothetical protein
M3Q_3086	3	4	DNA internalization-related competence protein ComEC/Rec2
M3Q_3099	3	3	glycosyltransferase
M3Q_3126	3	6	non-ribosomal peptide synthetase module
M3Q_3143	3	3	sphingosine kinase
M3Q_3169	3	5	hemolysin-type calcium-binding domain-containing protein
M3Q_3226	3	2	SAM-dependent methyltransferase
M3Q_3231	3	16	Pap2
M3Q_3245	3	8	chaperone protein HchA
M3Q_333	3	4	amino acid APC transporter
M3Q_3378	3	2	phospholipase D
M3Q_3386	3	21	hypothetical protein
M3Q_3417	3	3	Ig-like, group 1 precursor
M3Q_3447	3	5	hypothetical protein
M3Q_351	3	43	hypothetical protein
M3Q_3545	3	140	DNA metabolism protein
M3Q_3556	3	2	subtilisin-like serine protease
M3Q_3595	3	28	DNA binding and uptake competence factor ComC
M3Q_3619	3	5	Type II secretory pathway, component HofQ
M3Q_3626	3	2	hypothetical protein
M3Q_3691	3	2	hypothetical protein
M3Q_395	3	2	hypothetical protein
M3Q_4	3	5	transcriptional regulator
M3Q_444	3	5	TniB protein
M3Q_462	3	8	IS4 family transposase ORF 2
M3Q_470	3	7	transposition helper
M3Q_484	3	7	ATPase
M3Q_500	3	3	Type 4 fimbriae expression regulatory protein
M3Q_501	3	3	signal transduction histidine kinase
M3Q_562	3	3	alpha/beta hydrolase
M3Q_576	3	4	hypothetical protein
M3Q_60	3	141	hypothetical protein
M3Q_799	3	6	transcriptional regulator
M3Q_800	3	4	transcriptional regulator
M3Q_85	3	3	hypothetical protein
M3Q_883	3	20	long-chain fatty acid ABC transporter
M3Q_885	3	4	protein FilF
M3Q_891	3	21	Peptidase C13 family
M3Q_991	3	12	hypothetical protein

Supplemental Table 46 *A. baumannii* intergenic regions with multiple different IS elements inserted.

After Gene	Before Gene	#IS Elemen	#Genomes	After Gene Description	Before Gene Description
M3Q_2382	M3Q_2383	12	389	hypothetical protein	hypothetical protein
M3Q_2687	M3Q_2688	11	184	protein CsuA/B, secreted protein related to type I pili	TetR family transcriptional regulator
M3Q_313	M3Q_314	9	27	hypothetical protein	hypothetical protein
M3Q_1363	M3Q_1364	8	15	hypothetical protein	hypothetical protein
M3Q_1834	M3Q_1835	8	48	signal peptide protein	hypothetical protein
M3Q_190	M3Q_191	8	11	membrane-fusion protein	transcriptional regulator
M3Q_1903	M3Q_1904	8	31	fimbrial protein (pilin)	hypothetical protein
M3Q_3168	M3Q_3169	8	7	hypothetical protein	hemolysin-type calcium-binding domain-containing protein
M3Q_1490	M3Q_1491	7	11	hypothetical protein	metal ion ABC transporter substrate-binding protein/surface antigen
M3Q_1689	M3Q_1690	7	222	hypothetical protein	hemolysin
M3Q_2212	M3Q_2214	7	7	hypothetical protein	hypothetical protein
M3Q_2487	M3Q_2488	7	21	transcriptional regulator	iron-regulated membrane protein
M3Q_2679	M3Q_2680	7	22	TetR family transcriptional regulator	major facilitator superfamily permease
M3Q_880	M3Q_881	7	272	pilus subunit (FilA)	double-glycine peptidase
M3Q_1328	M3Q_1329	6	26	DNA-binding ATP-dependent protease La	hemagglutinin-like protein
M3Q_1632	M3Q_1633	6	10	beta-lactamase class A	Rhs element Vgr protein
M3Q_1663	M3Q_1664	6	156	hypothetical protein	signal peptide protein
M3Q_2067	M3Q_2068	6	10	O-succinylhomoserine sulfhydrylase	hypothetical protein
M3Q_2153	M3Q_2154	6	74	hypothetical protein	hypothetical protein
M3Q_2282	M3Q_2283	6	44	hypothetical protein	catalase
M3Q_2420	M3Q_2421	6	32	subtilisin-like serine protease	esterase/lipase
M3Q_2527	M3Q_2528	6	7	hypothetical protein	hypothetical protein
M3Q_2576	M3Q_2577	6	16	hypothetical protein	bifunctional aconitate hydratase 2/2-methylisocitrate dehydratase
M3Q_2695	M3Q_2696	6	94	threonine efflux protein	hypothetical protein
M3Q_312	M3Q_313	6	10	hypothetical protein	hypothetical protein
M3Q_3141	M3Q_3142	6	151	surface adhesion protein	phosphohydrolase
M3Q_41	M3Q_42	6	300	mutator family transposase	Lrp family transcriptional regulator
M3Q_1073	M3Q_1074	5	263	Tyrosine--tRNA ligase, putative	hypothetical protein
M3Q_1359	M3Q_1360	5	153	hypothetical protein	hypothetical protein
M3Q_1620	M3Q_1621	5	6	drug/metabolite transporter permease	EAL domain-containing protein
M3Q_2057	M3Q_2058	5	10	hypothetical protein	outer membrane protein (porin)
M3Q_209	M3Q_210	5	5	membrane-bound lytic murein transglycosylase B	phosphoribosylaminoimidazole carboxylase

M3Q_2123	M3Q_2124	5	11	hypothetical protein	H+/gluconate symporter family protein
M3Q_2159	M3Q_2160	5	9	catalase	hypothetical protein
M3Q_2378	M3Q_2379	5	13	Zn-dependent peptidase	ferrichrome-iron receptor
M3Q_2449	M3Q_2450	5	5	ornithine carbamoyltransferase	hypothetical protein
M3Q_2454	M3Q_2455	5	9	hypothetical protein	hypothetical protein
M3Q_2529	M3Q_2530	5	6	hypothetical protein	hypothetical protein
M3Q_2531	M3Q_2532	5	12	small-conductance mechanosensitive channel	TonB-dependent siderophore receptor
M3Q_2533	M3Q_2534	5	11	TetR family transcriptional regulator	hypothetical protein
M3Q_2669	M3Q_2670	5	33	glucose/sorbosone dehydrogenase	hypothetical protein
M3Q_2680	M3Q_2681	5	33	major facilitator superfamily permease	hypothetical protein
M3Q_2686	M3Q_2687	5	17	protein CsuA	protein CsuA/B, secreted protein related to type I pili
M3Q_2828	M3Q_2829	5	737	GTP cyclohydrolase I	IS4 family transposase
M3Q_2881	M3Q_2882	5	22	gamma-aminobutyrate permease	methylmalonic acid semialdehyde dehydrogenase
M3Q_3126	M3Q_3127	5	31	non-ribosomal peptide synthetase module	hypothetical protein
M3Q_3179	M3Q_3180	5	36	hypothetical protein	NhaP-type Na+/H+ and K+/H+ antiporter
M3Q_3469	M3Q_3470	5	12	nitroreductase-like oxidoreductase	hypothetical protein
M3Q_5	M3Q_6	5	13	arabinose efflux permease family protein	hypothetical protein
M3Q_531	M3Q_532	5	27	thioesterase	DNA binding protein
M3Q_566	M3Q_567	5	8	multifunctional fatty acid oxidation complex subunit alpha	beta-hydroxylase
M3Q_660	M3Q_661	5	10	transcriptional regulator	Zn-dependent hydrolase
M3Q_101	M3Q_102	4	8	PGAP1-like protein	methionine aminopeptidase
M3Q_1331	M3Q_1332	4	13	5,10-methenyltetrahydrofolate synthetase	signal peptide-containing protein
M3Q_1398	M3Q_1399	4	12	outer membrane receptor protein	Lipid A phosphoethanolamine transferase, associated with polymyxin resistance
M3Q_1493	M3Q_1494	4	11	TetR family transcriptional regulator	Rhs element Vgr protein
M3Q_1522	M3Q_1523	4	27	K+ transport protein	hypothetical protein
M3Q_1590	M3Q_1591	4	14	hypothetical protein	pyrroline-5-carboxylate reductase
M3Q_1600	M3Q_1601	4	7	hypothetical protein	surface antigen
M3Q_1625	M3Q_1626	4	8	hypothetical protein	transcriptional regulator
M3Q_1685	M3Q_1686	4	4	cyanate permease	hypothetical protein
M3Q_1759	M3Q_1760	4	9	acyl-CoA dehydrogenase	hypothetical protein
M3Q_1792	M3Q_1793	4	7	response regulator	acetyltransferase
M3Q_1802	M3Q_1803	4	14	major membrane protein I (MMP-I)	serine acetyltransferase
M3Q_1832	M3Q_1833	4	8	coenzyme F420-dependent N5,N10-methylene tetrahydromethanopterin reductase	transporter
M3Q_1853	M3Q_1854	4	11	L-asparaginase type II family protein	Na+/H+ dicarboxylate symporter
M3Q_1854	M3Q_1855	4	8	Na+/H+ dicarboxylate symporter	aspartate/tyrosine/aromatic aminotransferase

M3Q_1885	M3Q_1886	4	17	DcaP-like protein	transcriptional regulator
M3Q_1888	M3Q_1889	4	10	1-acyl-sn-glycerol-3-phosphate acyltransferase	transcriptional regulator
M3Q_1975	M3Q_1976	4	4	hypothetical protein	flavoprotein
M3Q_2061	M3Q_2062	4	11	hypothetical protein	hypothetical protein
M3Q_2071	M3Q_2072	4	21	ribonuclease D	hypothetical protein
M3Q_2157	M3Q_2158	4	20	hypothetical protein	hypothetical protein
M3Q_2168	M3Q_2169	4	10	P pilus assembly protein, pilin FimA	hypothetical protein
M3Q_2169	M3Q_2170	4	5	hypothetical protein	long-chain fatty acid transport protein
M3Q_2184	M3Q_2185	4	1	AraC-type DNA-binding domain-containing protein	alpha/beta fold family hydrolase
M3Q_2217	M3Q_2218	4	3	hypothetical protein	methylenetetrahydrofolate reductase
M3Q_2447	M3Q_2448	4	9	response regulator	transcriptional regulator
M3Q_2465	M3Q_2466	4	5	transcriptional regulator	molybdopterin biosynthesis protein
M3Q_2520	M3Q_2521	4	14	hypothetical protein	hypothetical protein
M3Q_2522	M3Q_2523	4	16	outer membrane protein W	TonB-dependent siderophore receptor
M3Q_2534	M3Q_2535	4	13	hypothetical protein	transcriptional regulator
M3Q_2675	M3Q_2676	4	33	paraquat-inducible protein A	permease
M3Q_314	M3Q_315	4	6	hypothetical protein	hypothetical protein
M3Q_3183	M3Q_3184	4	86	tryptophanyl-tRNA synthetase II	succinyl-CoA synthetase subunit alpha
M3Q_3219	M3Q_3220	4	6	outer membrane protein	hypothetical protein
M3Q_343	M3Q_344	4	21	hypothetical protein	transcriptional regulator
M3Q_344	M3Q_345	4	13	transcriptional regulator	acyl-CoA synthetase
M3Q_3475	M3Q_3476	4	3	hypothetical protein	TRAP-type C4-dicarboxylate transport system, large permease component
M3Q_3690	M3Q_3691	4	10	transcriptional regulator YdzF	hypothetical protein
M3Q_435	M3Q_436	4	41	outer membrane porin, OprD family	hypothetical protein
M3Q_462	M3Q_463	4	23	IS4 family transposase ORF 2	IS4 family transposase ORF 1
M3Q_601	M3Q_602	4	5	phaAB	ATP-binding protein
M3Q_614	M3Q_615	4	2	exodeoxyribonuclease V subunit alpha	penicillin-binding protein, beta-lactamase class C
M3Q_798	M3Q_799	4	69	diguanylate cyclase	transcriptional regulator
M3Q_800	M3Q_801	4	12	transcriptional regulator	hypothetical protein
M3Q_838	M3Q_839	4	1	acyl-CoA synthetase	threonyl-tRNA synthetase
M3Q_871	M3Q_872	4	16	lipoprotein precursor (VacJ) transmembrane	DNA-directed RNA polymerase specialized sigma subunit
M3Q_987	M3Q_988	4	6	hypothetical protein	threonine efflux protein
M3Q_1008	M3Q_1009	3	38	trehalose-6-phosphatase	alpha/beta superfamily hydrolase/acyltransferase
M3Q_1139	M3Q_1140	3	13	hypothetical protein	hypothetical protein
M3Q_1175	M3Q_1176	3	2	cytotoxic translational repressor of toxin-antitoxin stability system	hypothetical protein

M3Q_1201	M3Q_1202	3	7	site-specific DNA methylase	hypothetical protein
M3Q_1234	M3Q_1235	3	7	Na+/H+ dicarboxylate symporter	YaeQ protein
M3Q_1293	M3Q_1294	3	3	rubredoxin	hypothetical protein
M3Q_1303	M3Q_1304	3	13	citrate transporter, CitMHS family	hemolysin-like protein
M3Q_1324	M3Q_1325	3	2	glutamate dehydrogenase/leucine dehydrogenase	heat shock protein
M3Q_1345	M3Q_1346	3	2	hypothetical protein	hypothetical protein
M3Q_1364	M3Q_1365	3	12	hypothetical protein	hypothetical protein
M3Q_149	M3Q_150	3	3	histidine utilization repressor	acyltransferase
M3Q_1491	M3Q_1492	3	3	metal ion ABC transporter substrate-binding protein/surface antigen	hypothetical protein
M3Q_1492	M3Q_1493	3	4	hypothetical protein	TetR family transcriptional regulator
M3Q_1496	M3Q_1497	3	3	TPR repeat-containing SEL1 subfamily protein	TPR repeat-containing SEL1 subfamily protein
M3Q_1502	M3Q_1503	3	3	hypothetical protein	gamma-aminobutyrate permease
M3Q_1529	M3Q_1530	3	7	carbon storage regulator CsrA	aspartate kinase
M3Q_1537	M3Q_1538	3	284	oxidoreductase	IS4 family transposase ORF 2
M3Q_1553	M3Q_1554	3	4	glutathione S-transferase	trmB
M3Q_1566	M3Q_1567	3	4	Benzoate transport protein	benzoate transporter
M3Q_1592	M3Q_1593	3	19	hypothetical protein	hypothetical protein
M3Q_1593	M3Q_1594	3	2	hypothetical protein	hypothetical protein
M3Q_1599	M3Q_1600	3	15	hypothetical protein	hypothetical protein
M3Q_1601	M3Q_1602	3	12	surface antigen	cysteinyl-tRNA synthetase
M3Q_1643	M3Q_1644	3	2	chloride channel protein EriC	TPR repeat-containing SEL1 subfamily protein
M3Q_1688	M3Q_1689	3	4	hypothetical protein	hypothetical protein
M3Q_1780	M3Q_1781	3	6	hypothetical protein	competence/damage-inducible protein CinA
M3Q_1785	M3Q_1786	3	19	hypothetical protein	hypothetical protein
M3Q_1804	M3Q_1805	3	98	rhodanese-related sulfurtransferase	dehydrogenase
M3Q_1829	M3Q_1830	3	107	cytochrome bd ubiquinol oxidase, subunit I	hypothetical protein
M3Q_1879	M3Q_1880	3	7	acyl-CoA dehydrogenase	glutathione S-transferase
M3Q_1891	M3Q_1892	3	31	integrase	hypothetical protein
M3Q_1899	M3Q_1900	3	7	hypothetical protein	P pilus assembly protein, pilin FimA
M3Q_1912	M3Q_1913	3	305	Beta-lactamase class D	suppressor of F exclusion of phage T7 (FxsA)
M3Q_1920	M3Q_1921	3	124	hypothetical protein	hypothetical protein
M3Q_1986	M3Q_1987	3	53	hypothetical protein	dipeptide ABC transporter substrate-binding protein
M3Q_200	M3Q_201	3	3	lipoprotein	lipoprotein
M3Q_2022	M3Q_2023	3	5	hypothetical protein	ybaK/ebsC protein
M3Q_2060	M3Q_2061	3	12	hypothetical protein	hypothetical protein
M3Q_2062	M3Q_2063	3	8	hypothetical protein	hypothetical protein
M3Q_2063	M3Q_2064	3	6	hypothetical protein	hypothetical protein

M3Q_2085	M3Q_2086	3	20	lipoyl synthase	pyruvate/2-oxoglutarate dehydrogenase complex, dehydrogenase component subunit alpha
M3Q_2122	M3Q_2123	3	12	transcriptional regulator	hypothetical protein
M3Q_2152	M3Q_2153	3	272	Zn-dependent dipeptidase, microsomal dipeptidase	hypothetical protein
M3Q_2156	M3Q_2157	3	7	phosphatidylserine/phosphatidylglycerophosphate/cardiolipin synthase	hypothetical protein
M3Q_2181	M3Q_2182	3	4	response regulator AdeR	Signal transduction histidine kinase AdeS
M3Q_2204	M3Q_2205	3	7	RND family drug transporter	amidase
M3Q_2257	M3Q_2258	3	44	major facilitator superfamily permease	hypothetical protein
M3Q_2269	M3Q_2270	3	268	antibiotic biosynthesis monooxygenase	TetR family transcriptional regulator
M3Q_2278	M3Q_2279	3	18	NADH:flavin oxidoreductase	marR family transcriptional regulator
M3Q_2350	M3Q_2351	3	10	catI	2-C-methyl-D-erythritol 4-phosphate cytidylyltransferase
M3Q_240	M3Q_241	3	1	ABC transporter ATPase	RND type efflux pump
M3Q_2414	M3Q_2415	3	27	hypothetical protein	diguanylate cyclase
M3Q_2417	M3Q_2418	3	4	glucose/sorbosone dehydrogenase	hypothetical protein
M3Q_2418	M3Q_2419	3	4	hypothetical protein	sulfate permease
M3Q_2426	M3Q_2428	3	10	hypothetical protein	Fels-1 Prophage Protein
M3Q_2486	M3Q_2487	3	10	hypothetical protein	transcriptional regulator
M3Q_2530	M3Q_2531	3	274	hypothetical protein	small-conductance mechanosensitive channel
M3Q_2572	M3Q_2573	3	2	hypothetical protein	hypothetical protein
M3Q_2612	M3Q_2613	3	5	hemin-binding outer membrane transmembrane protein	hypothetical protein
M3Q_2641	M3Q_2643	3	36	Zn-dependent hydrolase	Na+/H+-dicarboxylate symporter
M3Q_2702	M3Q_2703	3	4	acyl-CoA dehydrogenase	hypothetical protein
M3Q_2792	M3Q_2793	3	5	methionine aminopeptidase	outer membrane protein W
M3Q_2793	M3Q_2794	3	6	outer membrane protein W	nitrate transporter transmembrane protein
M3Q_280	M3Q_281	3	6	FKBP-type peptidyl-prolyl cis-trans isomerase	tyrosine-protein kinase
M3Q_2869	M3Q_2870	3	114	drug/metabolite transporter permease	hypothetical protein
M3Q_2880	M3Q_2881	3	5	transcriptional regulator	gamma-aminobutyrate permease
M3Q_2954	M3Q_2955	3	6	subtilisin-like serine protease	periplasmic protease
M3Q_30	M3Q_31	3	15	diguanylate cyclase	34 kDa Outer Membrane Protein
M3Q_3063	M3Q_3064	3	7	Holliday junction DNA helicase subunit RuvB	amidohydrolase
M3Q_3093	M3Q_3094	3	7	phosphoserine aminotransferase	outer membrane protein
M3Q_3097	M3Q_3098	3	2	multidrug ABC transporter permease	hypothetical protein
M3Q_3125	M3Q_3126	3	4	hypothetical protein	non-ribosomal peptide synthetase module
M3Q_321	M3Q_322	3	6	hypothetical protein	hypothetical protein
M3Q_3235	M3Q_3236	3	15	DcaP-like protein	major facilitator superfamily permease

M3Q_3244	M3Q_3245	3	2	gamma-aminobutyrate permease	chaperone protein HchA
M3Q_3248	M3Q_3249	3	5	undecaprenyl-diphosphatase UppP	phenylacetic acid degradation operon negative regulatory protein
M3Q_3266	M3Q_3267	3	39	hypothetical protein	intracellular protease/amidase
M3Q_3292	M3Q_3293	3	17	response regulator with CheY-like receiver domain and winged-helix DNA-binding domain	hypothetical protein
M3Q_3394	M3Q_3395	3	3	Signal transduction histidine kinase	queA
M3Q_3633	M3Q_3634	3	18	sulfate/thiosulfate-binding protein	hypothetical protein
M3Q_643	M3Q_644	3	7	HopJ type III effector protein	50S ribosomal protein L31
M3Q_738	M3Q_739	3	62	acetate kinase	phosphogluconate dehydratase
M3Q_75	M3Q_76	3	166	hypothetical protein	TonB-dependent receptor protein
M3Q_799	M3Q_800	3	3	transcriptional regulator	transcriptional regulator
M3Q_879	M3Q_880	3	9	p-aminobenzoate synthetase	pilus subunit (FilA)
M3Q_927	M3Q_928	3	2	hypothetical protein	transcriptional regulator
